# Systematic analysis of somatic mutations driving cancer: uncovering functional protein regions in disease development

**DOI:** 10.1186/s13062-016-0125-6

**Published:** 2016-05-05

**Authors:** Bálint Mészáros, András Zeke, Attila Reményi, István Simon, Zsuzsanna Dosztányi

**Affiliations:** Institute of Enzymology, Research Centre for Natural Sciences, Hungarian Academy of Sciences, 2 Magyar Tudósok krt, Budapest, H-1117 Hungary; Lendület Protein Interaction Group, Institute of Enzymology, Research Centre for Natural Sciences, Hungarian Academy of Sciences, 2 Magyar Tudósok krt, Budapest, H-1117 Hungary; MTA-ELTE Lendület Bioinformatics Research Group, Department of Biochemistry, Eötvös Loránd University, 11/c Pázmány Péter stny, Budapest, H-1117 Hungary

**Keywords:** Cancer, Driver gene, Somatic mutation, Protein functional modules, Missense mutation, Insertion, Deletion

## Abstract

**Background:**

Recent advances in sequencing technologies enable the large-scale identification of genes that are affected by various genetic alterations in cancer. However, understanding tumor development requires insights into how these changes cause altered protein function and impaired network regulation in general and/or in specific cancer types.

**Results:**

In this work we present a novel method called iSiMPRe that identifies regions that are significantly enriched in somatic mutations and short in-frame insertions or deletions (indels). Applying this unbiased method to the complete human proteome, by using data enriched through various cancer genome projects, we identified around 500 protein regions which could be linked to one or more of 27 distinct cancer types. These regions covered the majority of known cancer genes, surprisingly even tumor suppressors. Additionally, iSiMPRe also identified novel genes and regions that have not yet been associated with cancer.

**Conclusions:**

While local somatic mutations correspond to only a subset of genetic variations that can lead to cancer, our systematic analyses revealed that they represent an accompanying feature of most cancer driver genes regardless of the primary mechanism by which they are perturbed during tumorigenesis. These results indicate that the accumulation of local somatic mutations can be used to pinpoint genes responsible for cancer formation and can also help to understand the effect of cancer mutations at the level of functional modules in a broad range of cancer driver genes.

**Reviewers:**

This article was reviewed by Sándor Pongor, Michael Gromiha and Zoltán Gáspári.

**Electronic supplementary material:**

The online version of this article (doi:10.1186/s13062-016-0125-6) contains supplementary material, which is available to authorized users.

## Background

Cancer genome projects use next generation sequencing technologies to identify somatic mutations – most often in exonic regions – that discriminate tumor cells from normal cells with the aim to understand the basis of the most common genetic disease [1–5]. The observed genetic alterations showed that the genetic landscape of cancer is complex, affecting a much larger number and varied types of genes than previously expected [1, 6]. There is also heterogeneity at the level of the underlying genetic mechanisms that lead to the variations. With advanced technologies, cancer genome projects are able to produce a more complete catalog of the variations. These include single point mutations and short insertions or deletions that can have a localized effect on a single gene and larger structural aberrations such as copy number alterations and genomic rearrangements that generally affect multiple genes. These data are cataloged in various databases, such as the COSMIC database, which now contains over millions of variations that are dominated by simple mutations [7, 8]. Most of the observed variations, however, correspond to randomly occurring passenger mutations. One of the key challenges in the interpretation of cancer genomics data is the identification of driver mutations that provide direct growth advantages in tumorigenesis, and distinguishing them from passenger mutations [3, 4].

Various computational approaches have been developed to identify driver mutations and driver genes in which they reside. Most commonly, individual genes are identified based on the predicted functional impact or the reoccurrence of mutations [3, 9]. In these later approaches, the number of mutations observed for each gene is compared to a background mutation rate. Originally, a uniform background mutation rate was used for the whole genome leading to many falsely identified driver genes. According to recent studies, however, the mutational background rate, which dictates the occurrence of stochastic mutations can also vary at the level of individual genes and depends on genomic location, expression level and replication time [10, 11]. The more recent version of the MutSig algorithm incorporates this mutational heterogeneity by using a context dependent estimation of the background mutation rate [10]. This estimation is based on the observed synonymous and intronic mutations for each gene that can be effectively used in tumors with high mutation rates only.

While most efforts traditionally aim at the identification of cancer drivers at the level of genes, often more insight can be gained by taking into account that the position and nature of observed mutations can often be translated to changes of protein function and structure. A fundamental property of proteins is that they can be composed of multiple functional modules that can include a combination of ordered domains and highly flexible intrinsically disordered regions [12, 13]. These specific regions can have independent functions and produce different phenotypic responses if disrupted. In accordance, it has already been observed that disease causing mutations are often clustered into specific regions of proteins, highlighting the role of specific functional modules in various diseases [14, 15].

The concept of identifying specific protein regions which contain a significant number of mutations has been already applied to identify cancer drivers. One method, OncoDriveClust [16] uses coding-silent mutations as background, and detects regions where mutations are clustered compared to this background. However, because of its suboptimal background model, this method often misses even frequently mutated regions, like the DNA binding domain of p53. Using an alternative approach, the e-Driver [17] method relies on predefined functional regions such as known domains or predicted disordered segments. From these segments, the method selects those that show a bias in their mutational rate compared to other functional regions within the same protein. Another type of approach incorporates information about the three-dimensional structure of proteins to evaluate the effect of mutations [18, 19]. Studying the effect of mutations at the level of specific regions gives us a better resolution and might serve as a better tool to understand the molecular principles of various diseases, including cancer [15, 18, 20]. A clear advantage of this approach is that a specific background of mutation frequencies can be calculated for each protein. However, current methods have limitations either because of biases towards known structure or domain assignments or due to an inaccurate background model.

Here we present a novel method called iSiMPRe (identification of significantly mutated protein regions) that is able to pinpoint proteins and protein regions that harbor a significant amount of cancer-related mutations in an unbiased manner. We consider mutations that carry precise and localized information of the affected region of proteins, thus include only missense mutations and in-frame insertions and deletions. Significantly mutated regions are identified using a unified statistical model for all three mutation types. Using the annotations of mutations it is also possible to tie significantly mutated protein regions to specific cancer types. We systematically analyzed the performance of iSiMPRe in identifying known cancer genes. We found that a surprisingly complete set of previously established cancer genes can be obtained by using the above mentioned limited set of genetic variations that directly affect protein regions. The main advantage of the new method is that it can pinpoint not only genes but also specific protein regions that are targeted by cancer mutations, even below the level of domains. The analysis of these regions helps to interpret the effect of cancer mutations at the level of functional regions.

## Results

### Significantly mutated protein regions are identified based on mutation pattern

**Fig. 1:**
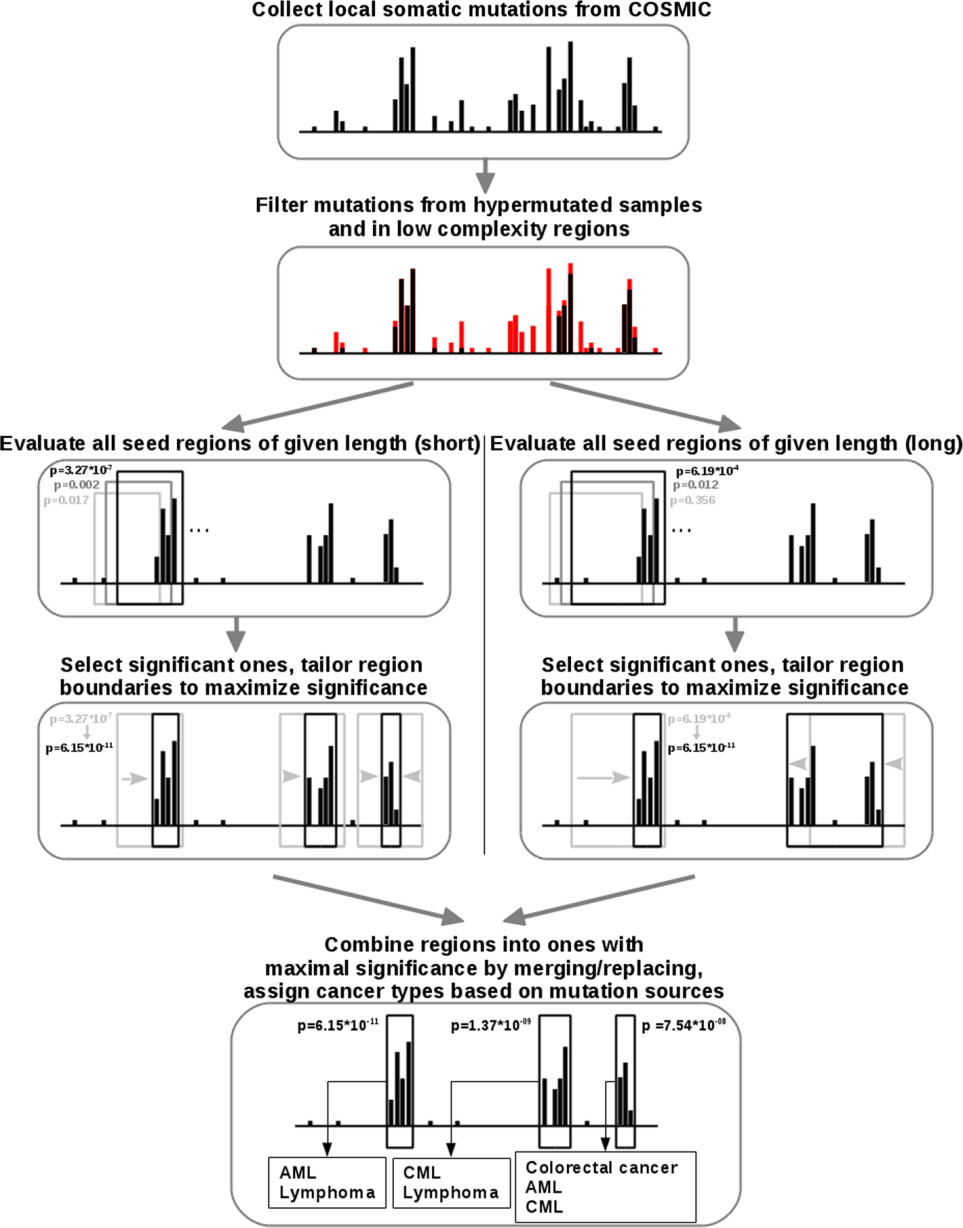
Outline of the method. All local somatic mutations are collected from the COSMIC database for a given gene, discarding mutations coming from hypermutated samples (see Methods) and mutations overlapping with low complexity regions. Next, a seed region in the corresponding protein sequence is selected and is assessed for significant enrichment of mutations compared to the expected random distribution using a one-sided Fisher’s exact test. Next, if the selected region is significant (*p*-value <0.01) its boundaries are moved to either side to locally maximize significance. This is repeated for all possible seed regions of 7, 10 and 30 residues in length. After the evaluation of all seed regions, the resulting optimized regions are merged if overlap occurs between them. For an exhaustive description of the algorithm see Additional file 1

In this work we collected cancer associated non-synonymous mutations from the COSMIC database. We developed a novel method called iSiMPRe that is able to identify cancer genes at the level of protein regions based on these mutations. The underlying assumption of iSiMPRe is that cancer mutations affecting protein coding regions are not distributed evenly, rather they are accumulated in specific regions (Significantly Mutated Protein Regions - SiMPRes) that play an active role in tumorigenesis. Regions that harbor a significantly enriched amount of somatic mutations compared to neutral local mutations can highlight not only cancer genes but also specific functional regions within them that actively contribute to the development of cancer. Since the mutation frequency is calculated for each gene separately from the observed number of mutations, different background mutation rates apply for each gene, that can take into account that the background mutation rate is different for individual genes [10]. In our analysis, we only considered exonic mutations that have a local effect, therefore only missense mutations and in-frame insertions and deletions are considered. The presented method is the first that is able to consider in-frame insertions and deletions as well as missense point mutations in a unified statistical framework. This sets it apart from two conceptually similar methods, eDriver [17] and OncoDriveClust [16], which do not consider in-frame insertions and deletions. The input of iSiMPRe is a set of cancer-related missense mutations and in-frame insertions and deletions. The background mutation rate is calculated simply from these using only a few empirical parameters. This is in contrast to OncoDriveClust which estimates the background mutation rate from a set of silent substitutions that has to be supplied as a separate set of input data. iSiMPRe is described in detail in Additional file 1 (iSiMPRe protocol) and in short in Fig. 1. In order to enable potential users to apply the method to updated versions of COSMIC datasets or other sources of cancer mutation data, the source code of iSiMPRe is available for download. In our experience, the identified significantly mutated regions change very little with updates of COSMIC datasets.

The large scale scanning of the COSMIC database yielded a total of 534 SiMPRes in 382 genes in the human proteome. A complete list of identified regions together with their significance levels, the number and type of mutations contained and respective cancer types is shown in Additional file 2. Highly significant mutated regions are usually found in well-established cancer genes such as BRAF and TP53. There are, however, only a limited number of high significance regions as most regions show a more moderate accumulation of mutations with medium to low significance (see Table 1). These results are in agreement with earlier observations that the mutational landscape of cancer is dominated by mutational hills with a few mutational mountains [1]. The length of the identified regions spans from 1 to 280 residues with an average of 20 residues. The identified regions on average cover 3.1 % of a protein’s sequence, with only a handful of regions exceeding 35 %. These data, indeed, indicate that cancer mutations are not distributed evenly within proteins, but are clustered within certain localized regions.

**Table 1:** Summary of identified SiMPRes. Regions are grouped according to their significance level (see Methods) and their dominant mutation type

		Dominant mutation type			
		Missense mutations	Insertions	Deletions	Total
Significance level	High significance	68	5	10	83 (15.5 %)
	Medium significance	77	0	13	90 (16.9 %)
	Low significance	285	9	67	361 (67.6 %)
	TOTAL	430 (80.5 %)	14 (2.6 %)	90 (16.9 %)	534 (100 %)

### Known cancer genes harbor significantly mutated regions

**Fig. 2:**
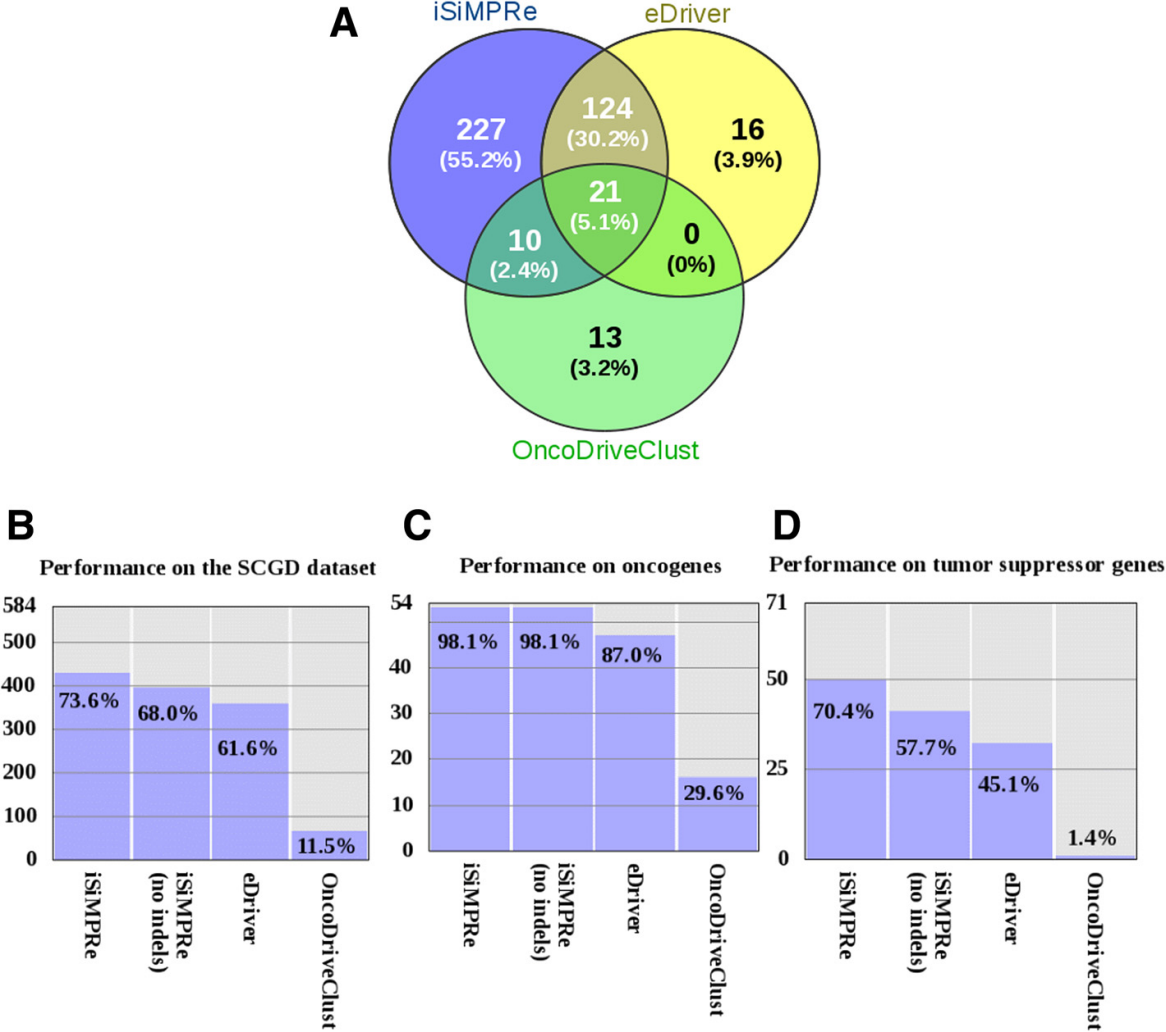
Comparison of the effectiveness and overlap between methods. Number of genes (**a**) identified by the different methods together with respective overlaps. The recovery rates of all three methods tested on the SCGD (**b**), oncogenes (**c**) and tumor suppressors (**d**)

We have tested the overlap between the identified SiMPRes and the cancer-related genes in the SCGD (see Methods), and separately on annotated tumor suppressors and oncogenes. iSiMPRe can identify at least one SiMPRe in 74 % of the genes in SCGD (Fig. 2). The performance of the method is nearly perfect for oncogenes with at least one identified region (53 out of 54, accounting for 98 %) with only one exception, DNMT1. In this case, the available mutational data is insufficient to yield high significance. Unlike its relative, DNMT3A, where mutations are highly enriched at a C-terminal region near the catalytic site, studies indicate that mutations of DNMT1 appear to be a rare event [21].

It is typically assumed that oncogenes harbor mainly missense mutations, while tumor suppressor genes are mainly affected by inactivating mutations, most often truncating nonsense or frameshift mutations [22]. As iSiMPRe considers only mutations that are traditionally linked to oncogenes and not to tumor suppressors, the recovery rate calculated on the latter group of genes is expected to be lower compared to oncogenes. In accordance with the hypothesis, a more moderate performance was observed in this case. Nevertheless, the majority of tumor suppressor genes (50 out of 71, accounting for 70 %) were still identified as containing at least one region with a significant accumulation of local somatic mutations. This is quite a striking result given that the contribution of missense mutations to tumor suppressor gene alterations is considered to be secondary to inactivating and truncating mutations [22].

### iSiMPRe outperforms already existing methods with the same scope

Additionally to iSiMPRe, two recently developed methods OncoDriveClust [16] and eDriver [17] were also tested. These methods - similarly to our approach - aim to identify regions where mutations are clustered in the amino acid sequence. The major difference between eDriver and iSiMPRe is that eDriver relies on regions with predefined boundaries based on domain annotations and disorder predictions, while iSiMPRe identifies regions that maximize mutational significance without relying on predefined regions. While the background model of eDriver is similar to the one used in our model, OncoDriveClust uses different input data that includes frameshift and nonsense mutations together with missense point mutations and compares it to the distribution of silence mutations.

Figure 2a shows the number of genes identified by the three methods together with the overlap between them. The overlap between iSiMPRe and eDriver is high with most eDriver genes and regions being identified by iSiMPRe as well. However, the overlap between genes identified by iSiMPRe and OncoDriveClust was significantly lower. In terms of performance a similar trend was followed for the three methods (Fig. 2b–d). Oncogenes were recognized best, while tumor suppressors are more challenging, especially for OncoDriveClust. In all three datasets, iSiMPRe achieves the best performance, followed by eDriver. The lower performance of concurrent methods can be traced back to various factors. In the case of eDriver, averaging mutation numbers in larger predefined regions can sometimes mask significant regions, which can have a negative effect on correctly identifying cancer driving genes. Trivially, no significantly mutated regions could appear in proteins composed of a single domain or one disordered region alone. The performance of OncoDriveClust was significantly lower, with only one tumor suppressor and only less than third of oncogenes identified. This lower performance of OncoDriveClust suggests that its expected mutation distribution statistical model should be revisited and that the larger scale effect of frameshift/nonsense mutations cannot be compared to the local effect of missense mutations and short indels.

### In-frame indels are important for finding cancer genes

The used version of the COSMIC database contains 404,903 missense mutations and 9977 in-frame insertions and deletions. While the number of short insertions and deletions is only less than 2.5 % of that of all local somatic mutations, they still make significant contribution to the identification of significantly mutated regions and contribute to the increased performance of iSiMPRe. These types of genetic alterations are dominant in about 20 % of the found regions, meaning indels in these cases contribute more to the significance of the region than missense mutations. In accordance, one-fifth of all regions are undetectable without taking indels into account. Omitting indels from the calculations would result in a decreased performance. Nevertheless, iSiMPRe would still outperform the other two methods (Fig. 2b–d). The incorporation of indel type mutations is especially important for the identification of tumor suppressors. Omitting indels does not have an impact on the recognition of oncogenes, but it does affect the results on both SCGD and tumor suppressors.

Some of the significantly mutated indel regions occur within genes with a well-established connection with cancer development, such as KIT, BRAF or PTEN. The proteins encoded by these genes harbor multiple SiMPRes, many of which are dominated by missense mutations, making the genes themselves identifiable using missense mutations alone. However, in certain cancer genes the primary mechanism of genetic alteration is the accumulation of short insertions or deletions. Discarding indels in the region identification process would make it impossible to reliably identify such known cancer genes. Notable examples include the interleukin-7 receptor subunit alpha (IL7R), which is known to be involved in the occurrence and development of various forms of acute leukemias and solid tumors [23]. Other examples include TSC2, where mutations have been linked to the development of hamartomas in multiple organs [24]; and ATAD5, the somatic mutations of which were identified in endometrial tumors [25].

### Other types of genetic alterations are also often associated with local somatic mutations

**Fig. 3:**
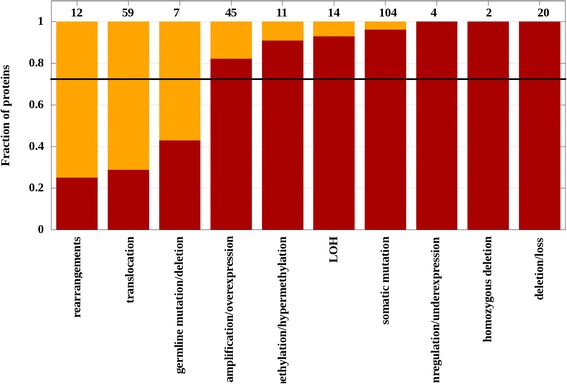
The ratio of proteins with significantly mutated regions in various classes of typical genetic alterations. Based on the KEGG annotation, genes are grouped according to the dominant genetic aberrations for a given gene. Red bars show the fraction of proteins with found regions, the horizontal black line shows the average ratio of 0.752. Numbers above bars show the number of known genes in the category. (LOH = Loss of heterozygosity)

Known cancer genes are defined based on various dominant type of genetic alterations and do not necessarily contain an accumulation of local somatic mutations. In order to gain insights into how the dominant type of genetic alteration for a given gene influences the chance of iSiMPRe to find a significantly mutated region at the protein level, the performance was further analyzed on specific datasets. For this analysis the KEGG database was used that provides information about a broad set of genetic alterations associated with specific genes. Figure 3 shows the ratio of genes for each type of dominant genetic alteration where SiMPRes were identified in the protein counterparts. Ten categories were analyzed in which somatic mutations represent only one of the categories. The results indicate that chromosomal translocations and rearrangements represent a roughly independent modulation of the genome and genes primarily subject to such changes are largely devoid of significantly mutated regions. Also, relatively modest recovery rate was observed for genes altered dominantly via germline mutations. In general, more than 80 % of the proteins dominantly altered by other mechanisms also contain at least one significant region identified by iSiMPRe in seven out of ten categories.

### Significantly mutated regions can be specific to one cancer type or can be associated with a broad set of cancer types

**Fig. 4:**
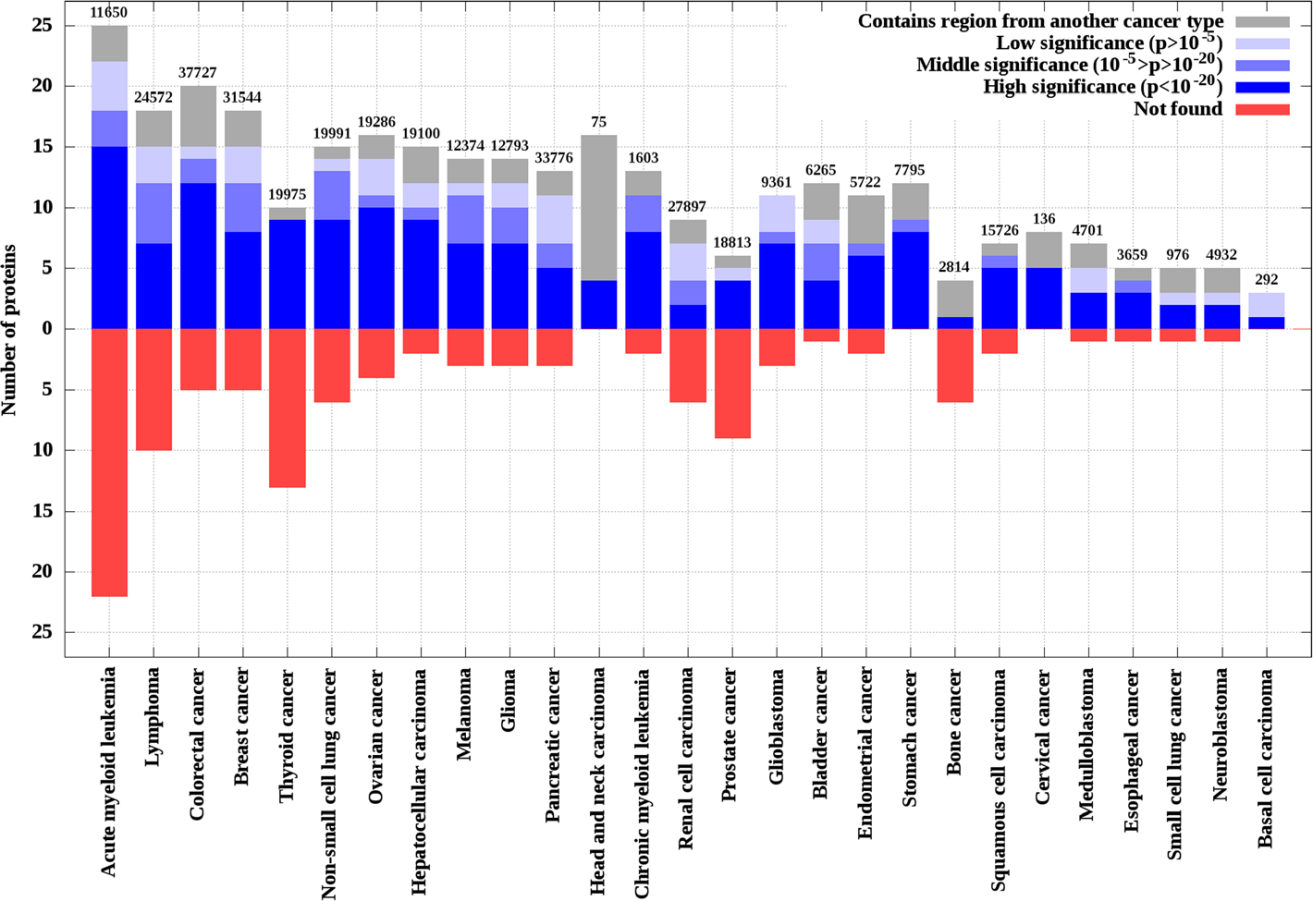
Overlap between cancer-related genes and significantly mutated regions. Blue bars show proteins that harbor at least one region annotated to the correct cancer-type. Shades of blue show the significance level of the most significant region found. Grey bars show the number of proteins that harbor at least one region but where the region is annotated to a different cancer type. Red bars show the number of proteins without significant regions. Numbers above the bars show the number of mutations in the COSMIC database annotated to the cancer type. Order of cancer types reflects the decreasing number of known genes from left to right

Based on the supplied annotations in the respective datasets, genes were linked to one or more of the 27 standardized cancer types (see Methods and Table 2). Taking advantage of this annotation, the performance of iSiMPRe could be evaluated for individual cancer types. Figure 4 indicates that the overlap between cancer genes and significantly mutated regions found by iSiMPRe is highly uneven across various cancer types. In certain cancer types, such as head and neck carcinomas, iSiMPRe is able to identify all known cancer genes, exhibiting a perfect performance. In other cases the recovery rate is lower (such as the 40 % in the case of prostate and bone cancer). However, iSiMPRe is able to recover 74 % of cancer genes on average with recovering at least one third of genes even in the poorest case. Although perfect performance can be seen in some cancer types with low numbers of COSMIC mutations (such as stomach cancer, head and neck carcinoma, basal cell carcinoma and cervical cancer); in general there is no obvious relationship between the recovery rates and cancer types, sample number or the number of mutations.

**Table 2:** Twenty-seven main cancer types. Cancer types are shown with the corresponding tissue/organ of occurrence and number of local somatic mutations and originating samples in COSMIC

Tissue/organ	Cancer types	Number of local somatic mutations in COSMIC	Number of samples	Average number of mutations per sample
Bladder	Bladder cancer	6 265	3 027	2.07
Blood	Acute myeloid leukemia	11 650	5 857	1.99
	Chronic myeloid leukemia	1 603	1 041	1.54
	Lymphoma	24 572	6 944	3.54
Bone	Bone cancer	2 814	706	3.99
Brain	Glioblastoma	9 361	2 909	3.22
	Neuroblastoma	4 932	619	7.97
	Glioma	12 793	2 947	4.34
	Medulloblastoma	4 701	728	6.46
Breast	Breast cancer	31 544	4 404	7.16
Cervix	Cervical cancer	136	132	1.03
Colorectal	Colorectal cancer	37 727	25 806	1.46
Esophagus	Esophageal cancer	3 659	433	8.45
Head and neck	Thyroid cancer	19 975	13 908	1.44
	Head and neck carcinoma	75	67	1.12
Kidney	Renal cell carcinoma	27 897	1 750	15.94
Liver	Hepatocellular carcinoma	19 100	2 091	9.13
Lung	Small cell lung cancer	976	208	4.69
	Non-small cell lung cancer	19 991	11 236	1.78
Ovary	Ovarian cancer	19 286	2 936	6.57
Pancreas	Pancreatic cancer	33 776	5 609	6.02
Prostate	Prostate cancer	18 813	967	19.46
Skin	Melanoma	12 374	7 923	1.56
	Squamous cell carcinoma	15 726	3 459	4.55
	Basal cell carcinoma	292	251	1.16
Stomach	Stomach cancer	7 795	1 430	5.45
Uterus	Endometrial cancer	5 722	1 627	3.52
Total		353 555	109 015	3.24

**Fig. 5:**
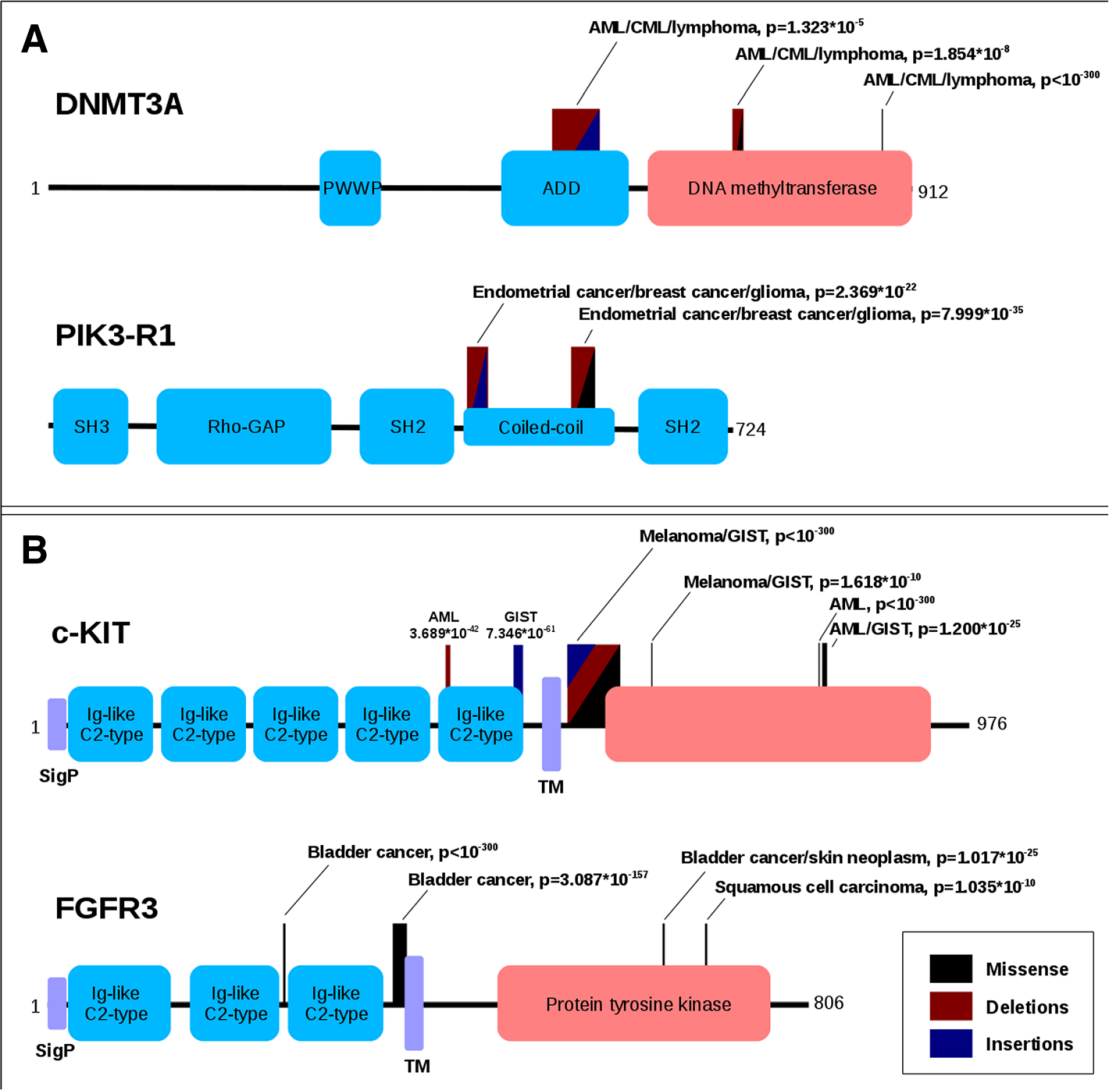
Proteins with multiple regions covering multiple cancer types. Symmetrically positioned boxes represent structural/functional protein units: *grey* – signal sequence, *black* – transmembrane region, various colors – domains (*red* = catalytic domains, *blue* = all other domains, with abbreviated names written in the box). Boxes above the line represent significantly mutated regions. Colors denote dominant mutation types: *black* – missense, *red* – deletions, *blue* – insertions. Regions are flagged with dominant cancer types together with the *p*-value of the region. GIST – Gastrointestinal stromal tumor, AML – Acute Myeloid Leukemia, CML – Chronic Myeloid Leukemia. All examples feature multiple regions involved in the same cancer types (**a**) or multiple cancer-specific regions (**b**)

At the level of individual genes, cancer genes can be specific to one or a very limited number of (usually related) forms of cancer or can be involved in multiple cancer types. In a number of cases, multiple regions showed a significant enrichment of local mutations within a single gene. In these cases two basic scenarios could emerge. In the first scenario, mutations from various tissue samples are typically distributed in roughly the same way and their accumulation along the sequence outline the same functional regions. This general trend is demonstrated in Fig. 5a in the case of DNA methyltransferase 3A (DNMT3A) and for phosphatidylinositol 3-kinase regulatory subunit alpha (PIK3-R1). In the case of DNMT3A, the dominant part of mutations come from various hematopoietic cancers, such as acute myeloid leukemia (AML), chronic myeloid leukemia (CML) or lymphoma. These mutations cluster in three distinct regions: one region falls into the middle of the ADD (ATRX-DNMT3-DNMT3L) domain, responsible for the interaction with the polycomb repressive complex 2 (PRC2), and it roughly covers the PHD-type zinc finger; the second and the third regions both fall into the catalytic (DNA cytosine methyltransferase) domain. In all three regions, the source of these mutations is fairly homogeneous with respect to the originating cancer types, even though the dominant mutation types differ for the three regions (deletions + insertions, deletions + missense and missense only, respectively). Similarly, the mutations in PI3K-R1 outline two distinct regions, covering the two terminal parts of the inter-SH2 coiled-coil region, which is responsible for the interaction with the catalytic subunit. Similarly to the DNMT3A case, the regions differ slightly in their dominant mutation types; however, in all regions the majority of the mutations come from the same, endometrial and breast cancer, samples.

Less frequently, a different scenario can also be observed, where a single gene is associated with different types of cancer depending on the location of the mutations. The most interesting examples involve certain tyrosine kinase receptors. Figure 5b shows the domain structure and the significantly mutated regions encountered in c-KIT and FGFR3. Both are single-pass transmembrane proteins: in their extracellular region they contain multiple Ig-like domains, while their intracellular regions harbor a tyrosine kinase domain. The latter element is one of the most frequently mutated domains in various cancers (Table 3) and it also contains significantly mutated regions. However, other regions in these two proteins also exhibit an increased number of genetic variations that can involve mostly missense mutations, deletions, insertions or mixed types of variations. Importantly, these specific regions are associated with different cancer types. In the case of KIT, the majority of known somatic mutations were sequenced in AML, melanoma and gastrointestinal stromal tumor (GIST) samples. The two regions found in the last Ig-like domain of the extracellular receptor part is linked to AML and GIST, respectively. The intracellular regions are either GIST, AML or melanoma specific, or a combination of two of the three cancer types, but interestingly KIT does not seem to harbor a ubiquitously mutated region. Another uncommon cancer-type specific partition of mutations can be seen in the case of FGFR3, where both extracellular regions are linked exclusively to bladder cancer while the intracellular regions are present in various forms of skin tumors as well.

**Table 3:**
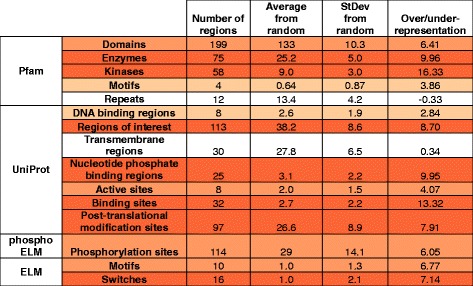
The occurrence of SiMPRes in known structural/functional protein sites/regions. Color codes represent over- and under-representation compared to random. Shades of red show increasing over-representation. The amount of over- and under-representation is given in standard deviation units calculated from 1000 randomly assigned regions. ‘Regions of interest’ marks experimentally characterized protein regions that can be of interest concerning protein function (such as interaction sites, different regions of multifunctional enzymes or regions crucial for biological processes/sub-cellular localizations)

### Significantly mutated protein regions correspond to a diverse set of functional modules

The identified SiMPRes enable us to take a deeper look into which functional modules of proteins are involved in cancer development. For this, we collected biological annotations of the identified regions/genes. Information was collected from UniProt database [26] which is a rich source of information about active sites of enzymes and additional functional sites including nucleotide phosphate-, DNA- and other binding regions. Another annotation category corresponds to ‘regions of interest’. This category used by UniProt can refer to basically any experimentally characterized protein region that can be of interest concerning protein function (such as interaction sites, different regions of multifunctional enzymes or regions crucial for biological processes/sub-cellular localizations). UniProt annotations were complemented with annotations from the Pfam database [27] collecting evolutionarily conserved protein family information with associated functions and from the ELM database which provides information about linear motifs operating largely outside of globular domains [28]. The occurrence of SiMPRes in various types of annotations were tested for over and under-representation using a basic statistical model (see Methods).

In most cases the identified SiMPRes could be associated with some structural or functional annotations. The mean overlaps and standard errors of randomized regions is shown in Table 3 together with the overlaps of real SiMPRes. The data in Table 3 shows that local genetic alterations are significantly concentrated on the structured parts of proteins. This is in accordance with earlier findings [29]. Within structured domains, there is a clear tendency for enzymes, especially for kinases, confirming earlier observations [30, 31]. The most commonly mutated domain is the Tyrosine kinase domain observed in 20 different proteins, including such well-known cancer driver genes as ALK, EGFR or BRAF (see Additional file 3). Other domains mutated in multiple cases include the Serine/threonine-protein kinase domain (e.g. in Activin receptor type-1 or in Serine/threonine-protein kinase STK11) and the SH2 domains (e.g. in PIK3-R1 and STAT3). In theory, the easiest way to disrupt enzyme function would be the modulation of the active site via the introduction of mutations. However, data show that the number of active sites overlapping with SiMPRes is much lower than the number of targeted enzyme/kinase domains. Instead, iSiMPRe showed a highly significant overlap with other types of functional regions, including nucleotide phosphate-, DNA- and other binding regions, as well as ‘regions of interest’. Protein binding regions were suggested to be the primary hot-spots for tumor suppressor proteins [15]. The high accumulation of mutations was not restricted to structured protein modules. The non-globular parts of proteins – especially motifs, motif switches and post-translational modification sites – tended to coincide with a high accumulation of mutations as well [32, 33].

## Discussion

### The search for cancer driver genes continues

Cancer emerges due to genetic and epigenetic changes. With the dramatic expansion of catalogued mutations detected in diverse tumor samples and the advent of exome sequencing screens, we have an overwhelming amount of data on our hands. Although this – in theory – should enable easier insights into tumorigenesis, it is still not a simple task to adequately distinguish between relevant, cancer-associated mutations and background genetic alterations without clinical significance. Therefore, one of the major focuses of cancer research is still the identification of genes that are responsible for cancer formation, tumor progression and metastasis. However, the definition of these cancer genes is far from trivial. Usually a prerequisite of a cancer driver gene is that its genetic alterations should have a positive contribution to the development of cancer. In practice, the decision of whether a gene fulfills these criteria or not can depend on the cancer type and the number of samples already analyzed, as well as the type of genetic alterations analyzed. It was also suggested that significantly larger sample size is needed to obtain a complete set of cancer driver genes [34]. The subjectivity of cancer drivers is transferred to the publicly available databases in which many such genes are collected. These datasets use different levels of evidence for the inclusion of a gene or gene product and they can also contain erroneously identified cancer genes, for example based on mouse models or paralog sequences. Since currently there is no consensus on the list of cancer genes, we used four different sources to collect and combine manually assembled and curated sets of cancer genes (see Methods and the figure in Additional file 4).

The lack of consensus regarding cancer genes is evident from the limited overlap between the four included datasets. The fact that only around 27 % of OMIM genes have support in other databases can be attributed to the fact that OMIM is primarily aimed at collecting germline affected genes that only partially overlap with somatically altered genes. Unexpectedly, even more limited overlap was observed in the case of COSMIC census database. For this dataset over half of the listed genes were absent from all other databases, as opposed to the 20 % and 4 % of the genes listed in KEGG and Driver genes, respectively. It could mean that the COSMIC census list includes novel cancer genes that are not present in other datasets as of yet, or it could also indicate lack of supportive evidence and possible biases of the inclusion system.

In addition to manually curated databases, various computational methods have also been developed to identify cancer driver genes [35, 36]. Most commonly used approaches seek to identify cancer driver genes either in the context of pathways and protein interaction networks [37] or by detecting signs of positive selection at the level of genes. Such methods can be based on the increased number of observed mutations compared to the background mutation rate [10]; a high rate of non-silent mutations compared to silent mutations [2]; or on the bias towards the accumulation of mutations with high functional impact [38]. However, the accumulation of mutations can highlight not only genes but also specific functional regions at the protein level that are involved in disease development. Recent methods, such as OncoDriveClust [16] or e-Driver [17] identify putative cancer driver genes based on this concept, similarly to the iSiMPre method presented in this work. Clustering of mutations can also be observed in three-dimensional protein structures that often correspond to perturbed protein-protein interaction sites [18, 19]. Given the complexity and heterogeneity of the molecular basis of cancer, the combination of different signals of positive selection can more reliably indicate mutational drivers [39]. Nevertheless, in our experience, the iSiMPre method is able to identify the majority of cancer driver genes based on the clustering of mutations and outperforms methods with similar scope. The increased performance of iSiMPre can be attributed to several factors, including the cleaning of mutational data (e.g. eliminating likely neutral polymorphisms and mutations occurring within tandem repeats that are more likely to accommodate neutral mutations as well as sequencing errors). Additional factor is the incorporation of all genetic variations with positional information, which include short in-frame insertions and deletions while excluding frameshift and non-sense mutations. iSiMPre is based on an unbiased approach that does not rely on previous knowledge of structure or domain, which could be especially important to detect cancer driver mutations located in intrinsically disordered proteins for example (manuscript in preparation).

Based on the presented analysis, the local accumulation of somatic mutations detected by iSiMPRe can also be used to pinpoint novel genes not yet (fully) represented in the available databases. We found 7 genes that contained high significance mutated regions that were absent from SCGD. In these cases, the high significance of the enrichment of somatic mutations lends a very strong support that these proteins indeed correspond to true cancer drivers. These include the lymphoma gene IL7R, the K^+^ channel KCNJ5, a hyperaldosteronism-linked gene that is also known to be mutated in adrenal cancers [40], CD79B known to be involved in B-cell lymphomas [41], the breast cancer gene ESR1 [42], the cytokine receptor IL6ST known to be involved in a range of cancers, the known cancer gene RAC1 regulating cell motility and RHOA also involved in various cancers, eg. gastric carcinomas [43]. For these 7 genes that are unique to the COSMIC census dataset, iSiMPRe confirms their cancer driver statuses.

Moving towards lower significance levels, the list of novel cancer genes becomes more populated. Altogether, 23 novel genes are identified by iSiMPRe with medium significance level, 18 of which are included in only one dataset. In these cases, the presence of a reliable region and the inclusion in one cancer gene list is a very strong indication of being a true cancer gene. This notion is supported by genes such as the checkpoint kinase 2 (CHEK2) and tuberin, which have been described as tumor suppressors; or CSF3R which has been described as an oncogene [44]. The other 15 genes harboring medium significance regions are even less represented in cancer gene datasets as they are absent from all four studied databases. These genes are shown in Table 4. Although missing from the studied databases, there is at least some indication of the genes’ involvement in cancer in the majority of cases. For RP1L1, recent results might offer a link between mutations in this gene and the development of gastric and colorectal cancers [45]. Similarly, mucin 6 is known to be linked to various forms of cancer [46, 47]. For other genes, their involvement in cancer is only hinted at in very preliminary studies (FRG1B) [25, 48, 49]. In addition, a link between WASH3P and cancer is supported by our findings; since mutations in a region indicate involvement in NSCLC and renal cell carcinoma.

**Table 4:** Medium significance region genes that are absent from all somatic cancer gene databases

Gene	Region	*p*-value	Dominant cancer type(s)	Protein name	Protein annotations	Region annotations	Indication of involvement in cancer
WASH3P	368–410	1.050*10^−14^	Renal cell carcinoma	Putative WAS protein family homolog 3	Pseudogene homolog of WASP, nucleation-promoting factor of endosomes	Missense mutations affect mainly one position. Region is part of Pfam-B conserved accross wide range of eukaryotes and probably disordered	Some indication of possible involvement in tumors (PMID: 21208217)
FRG1B/C20orf80	40–101	7.492*10^−14^	Prostate cancer, Glioma	Protein FRG1B	Unknown	Well distributed missense mutations in structured FRG1 domain, no known function, but conserved across eukar + some bact.	Only based on mutation pattern, no cancer specific annotations
ANKRD36C/ENSG00000174501	626–634	2.620*10^−12^	Prostate cancer, Glioma	Ankyrin repeat domain- containing protein 36C	Unknown	Well clustered missense mutation peaks in an unannotated, possibly disordered region of the protein	Very pleriminaty indication of possible role in various cancer types
ZNF814	337-337	6.451*10^−11^	Pancreatic cancer, Squamous cell carcinoma	Putative uncharacterized zinc finger protein 814	Acts as a trascription factor with specific DNA binding	Sharp peak of missense mutations N-terminal of the zinc binding domains	Very pleriminaty indication of possible role in some cancer types
RP1L1	1305–1361	1.654*10^−9^	Various	Retinitis pigmentosa 1-like 1 protein	Involved in axoneme assembly, photoreceptor cell development and retina development in camera-type eye	Broad peak of missense mutations and indels in the central, possibly disordered region of the protein	Indication of involvement in gastric and colorectar cancers (PMID: 23237666)
RRN3P2/ENSG00000103472	368–375	1.676*10^−9^	Prostate cancer	RRN3 homolog, RNA polymerase I transcription factor pseudogene 2	Unknown	Sharp peak of missense mutations in the RRN3 domain	Unknown
MUC6	1873–1995	3.663*10^−7^	Prostate cancer	Mucin 6	Modulates the composition of the protective mucus layer. Important in the cytoprotection of pithelial surfaces, used as tumor markers in a variety of cancers. May play a role in epithelial organogenesis.	Broad peak of missense mutations in a possibly disordered region of the protein	Known to be linked various forms of cancer (PMID: 21851820, PMID: 9650551)
EEF1B2	43-43	3.739*10^−7^	Prostate cancer	Elongation factor 1-beta	Translation elongation factor, guanine nucleotide exchange factor involved in the transfer of aminoacylated tRNAs to the ribosome	Sharp peak of missense mutations in the N-terminal region of the protein	Unknown
POTEC	477–511	4.504*10^−7^	Prostate cancer	POTE ankyrin domain family member C	Unknown	Multiple peaks of missense mutations in the C-terminal disordered part of the protein, encompassing a possible DNA binding motif	Unknown
EIF1AX	2–15	1.457*10^−6^	Thyroid cancer, Melanoma	Eukaryotic translation initiation factor 1A, X-chromosomal	Required for maximal rate of protein biosynthesis, enhances ribosome dissociation	N-terminal disordered region, harboring many missense mutations	Indication of involvement in melanoma (PMID: 24423917)
CS	183–187	1.670*10^−6^	Bile duct/gallbladder cancer	Mitochondrial citrate synthase	Involved in step 1 of the subpathway that synthesizes isocitrate from oxaloacetate	Well localized peaks of missense mutations in the citrate synthase domain	Indication of involvement in some cancers (PMID: 19647716)
RGPD8	1760-1760	2.200*10^−6^	Prostate cancer, Glioma	RANBP2-like and GRIP domain-containing protein 8	Unknown	Single peak of missense mutations at the C-terminal, possibly disordered region	Very pleriminaty indication of possible marker role in some cancer types
KRTAP4-9	57-57	3.407*10^−6^	Breast cancer	Keratin-associated protein 4–9	Part of an interfilamentous matrix, in which hair keratin intermediate filaments are embedded	Peak of missense mutations	Located in a potential breakpoint initiating ERBB2 amplification, which is known to be involved in breast cancer (PMID: 23181561)
KRTAP4-8	95-95	5.261*10^−6^	Glioma	Keratin-associated protein 4–8		Peak of missense mutations	
KRTAP9-9	18–30	9.921*10^−6^	Pancreatic cancer, Breast cancer	Keratin-associated protein 9-9		Short region dominated by indels	

iSiMPRe also identified 211 low significance regions that reside in genes not present in any of the cancer gene datasets (see table in Additional file 2). Although some of these genes have recently been linked to tumorigenesis (eg. FRK was shown to be involved in hepatocellular adenomas, which matches our annotations [50]), most genes have no direct indication of being cancer genes. Some of these candidate genes might be linked causatively to tumorigenesis, but they can only be expected to exhibit a weak phenotype (small growth advantage versus wild-type cells) in agreement with their lower mutation hit rate. However, they can also correspond to genes with a locally increased passenger mutation rate, without true biological significance. In these cases more data is needed to be able to discriminate low significance regions that might correspond to false positives from those regions that are genuinely involved in various cancers even though just with a weak phenotype.

Our results indicate that iSiMPRe is able not only to identify known and novel cancer genes, but the assigned confidence levels of the identified regions correlate well with our current knowledge of cancer driver genes. Furtermore, iSiMPRe can also effectively target the main issue of interpreting genomic sequencing data: the discrimination of driver and passenger mutations. Overall, the regions marked by iSiMPRe only contain about one third of the mutations listed in COSMIC, which is a drastic reduction of data. As there is a significant association between identified regions and functional protein regions, iSiMPRe may be useful to help in classifying mutations as drivers or passengers; simply based on their location inside or outside of a SiMPRe.

The fundamental differences between passenger and driver mutations can influence the set of preferential amino acid substitutions. In recent analyses, COSMIC mutations in the case of EGFR [51] and also for all genes [52] showed an uneven distribution of missense substitutions among cancer driver mutations defined as reoccurring mutation compared to likely passenger mutations recorded only once in COSMIC. Partitioning driver and passenger mutations based on iSiMPRe showed good agreement with preferential distributions observed earlier (see Additional file 2), with the seven and 16 most frequent substitutions appearing in both lists for driver and passenger mutations respectively. These results also hint at the partitioning power of iSiMPRe between driver and passenger missense mutations.

### The knowledge of significantly mutated regions involved in cancer can guide treatment and drug development choices

One of the main advantages of the method proposed in this work is that it is able to identify not only cancer driver genes but also specific regions that are involved in the disease. The importance of this more detailed view is apparent in cases where a single protein has multiple regions that are specifically mutated in different cancer types. An example for this behavior is exhibited in the case of cytokine receptors (Fig. 5b). In these cases various forms of cancer target different protein regions, which implies a tissue-specific biological selection for particular mutations. Unfortunately, little is known about the mechanistic differences of the same receptors in different tissues that would explain the selection for non-overlapping mutational hotspots. One possible explanation could be the homo- or heterodimerization of the same receptor with different partners in different tissues. Facultative receptor tyrosine kinase heterodimerization is a common phenomenon and already documented for several related proteins [53–55].

The current collections of genes that have been causatively linked to tumor formation are heterogeneous concerning their dominant genetic alterations by which their structure/function may be modulated. An important question is how well genes containing SiMPRes (calculated from somatic missense mutations and short in-frame indels) represent all cancer genes. A surprising outcome of our analyses was that significantly mutated regions can be found in the majority of cancer genes, regardless of their dominant genetic alterations (Figs. 3 and 4). The two main exceptions are chromosomal translocations and rearrangements, which represent roughly independent modulations of the genome (Fig. 3). In many other cases, however, somatic mutations can have similar effects to other types of genetic alterations. (Note that the detailed analysis of non-coding intergenic, promoter or intronic mutations was not done, yet the data published by other groups hints that the targets of these alterations might also be somewhat separate from those targeted by somatic mutations [56–58].)

**Fig. 6:**
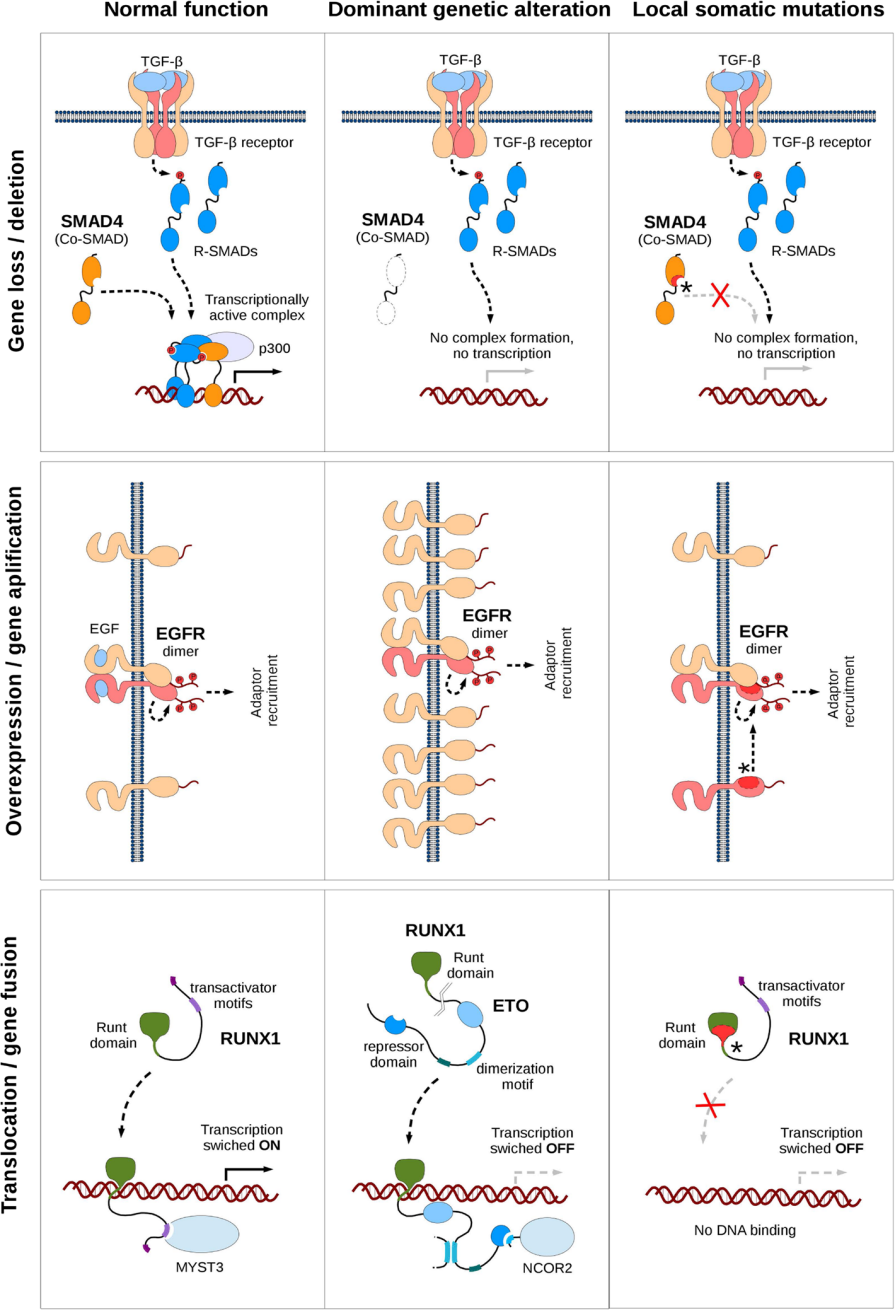
Connection between various genetic alterations and significantly mutated regions. Rows correspond to various types of genetic alterations. Columns from left to right show normal protein function, protein function modulated by the given genetic alteration and protein function modulated by the occurrence of significantly mutated regions

The observed interchangeability of various genetic alterations and the accumulation of somatic mutations is relatively straight-forward in the case where the dominant alterations diminish or abolish protein function (eg. deletion/loss, downregulation/underexpression and promoter hypermethylation). An example is provided by the SMAD4 gene (Fig. 6, top row). In this case the clustering of somatic mutations affects the C-terminal MH2 domain that is essential for both homo- and hetero-oligomerization. Normally SMAD4 transmits signals in the TGF-beta pathway, which is a negative regulator of epithelial growth. The deletion of the SMAD4 gene or the abolishment of the protein function via somatic mutations leads to cancer through the breaking of a negative regulatory pathway.

In the case of genetic alterations that enhance protein function, the interchangeability with missense mutations is slightly less evident. Overexpression/amplification typically can happen in receptors containing intracellular kinase domains that are able to modulate various pathways (such as ERK, JNK, Akt, etc.) via their downstream targets. Both amplification and their accumulated local somatic mutations force these proteins to be constitutively active independently from the binding of their extracellular ligands. For example in the case of EGFR (Fig. 6, middle row) the active form of the receptor is brought about by homo- or hetero-dimerization, which is normally achieved by the binding of EGF/TGFα (or other ligands). This dimerization can occur without ligand binding when the protein is overexpressed; however, the same effect can also be brought about by the accumulation of local somatic mutations in the N-terminal part of the intracellular kinase domain.

As opposed to the previously discussed genetic alterations, in general the effects of translocations and chromosomal rearrangements cannot be understood through the function of a single gene/protein. The resulting fusion protein can carry functional parts from both original proteins and can fulfill novel biological roles. In most cases, the effect of translocations cannot be effectively mimicked by the accumulation of local mutations. However, in certain cases, genes undergoing translocations as their primary genetic alteration still show a significant accumulation of somatic mutations. A prime example for this behavior is presented by RUNX1 (Fig. 6, bottom row). This gene encodes a transcription factor that consists of the runt domain that is responsible for the interaction with the DNA and a long, presumably disordered transactivation region that binds to partner proteins. A common genetic alteration of RUNX1 is the fusion of the runt domain coding segment (first 5 exons) of the gene to the almost complete ETO gene. The resulting chimeric protein termed AML-ETO retains the ability to bind RUNX1 target genes while exerting the transcription repression function of ETO, silencing RUNX1 genes contributing to the development of various hematopoietic cancers, most notably AML. Apart from translocation, the RUNX1 gene also harbors significant accumulation of somatic mutations in the runt domain. The majority of these mutations were derived from AML samples indicating the possibility of these mutations mimicking the effect of the translocation. The mutation hotspots in the runt domain coincide with three regions that are directly involved in DNA binding mostly affecting arginine/lysine residues that are necessary for the charge complementarity. This diminishes the DNA binding capability of RUNX1 and inhibits the activation of its target genes, achieving the gene silencing effect of the gene fusion.

## Conclusions

The observed somatic mutations collected across diverse tumor samples tend to cluster in functional protein regions that play a key role in tumorigenesis. This was exploited to construct an algorithm – iSiMPRe – which takes the list of the observed non-synomymous mutations and automatically identifies not only potential cancer driver genes, but also specific regions that are involved in the disease development. iSiMPRe not only outperforms other available methods, but its success also shows that the limited range of mutations considered (somatic exonic mutations that have a local effect) is enough to identify the majority of known cancer genes with a wide variety of typical genetic alterations (somatic gene deletions, gene amplifications, over- or under-expression, etc.). This way, the accumulation of somatic mutations can also offer potential sites for drug development efforts even in cases which are generally altered by more complex genetic mechanisms. Furthermore, most tumor suppressors also show local enrichments of missense mutations, just as proto-oncogens do. Nevertheless, a subset of cancer driver genes cannot be identified solely based on their enrichment of local somatic mutations. These typically involve genes dominantly altered by chromosomal translocations or rearrangements, or a subset of tumor suppressor genes that are altered mostly by truncating mutations. The complete recovery of cancer driver genes therefore requires a combined approach [39].

In the future, iSiMPRe could be a useful tool to identify genes and proteins where local biological selection affects their function in a selected way. This could not only be utilized to find (causatively) cancer-associated genes, but also to study their domains and other sub-molecular functional elements directly affected by mutations, even when no structural or functional annotation is available. Furthermore, apart from data supplied by cancer sequencing efforts, mutation data from any other illness targeted by systematic studies (Crohn’s disease [59], autoimmunogenic disorders [60], autisms [61] and others [62, 63]) can be used as input, thus iSiMPRe can be used to analyze the genetic background of rare hereditary diseases by linking phenotypic observations with protein function directly. The widely used approach of considering whole genes/proteins is problematic as these are often pleitropic, while for regions pathological involvement is more direct. This way iSiMPRe can be directly used to gain invaluable insight into the patomechanisms of diseases as well.

## Methods

### COSMIC somatic mutations

Cancer related somatic mutations were collected from version 73 of the COSMIC database (http://cancer.sanger.ac.uk/cosmic/) [8]. The complete dataset contained 20,337 protein sequences with at least one annotated mutation in the 73 version of the COSMIC database. All missense point mutations together with in-frame insertions and deletions were collected and checked against the protein sequences given in COSMIC. Mutations that were sequenced in samples having over 100 mutations were discarded as hypermutated samples, as these likely include a large fraction of passenger mutations. Furthermore, point mutations coinciding with positions of common single nucleotide polymorphisms were discarded using SNP data collected from the UCSC Genome Browser [64]. In order to filter out clonal variants, samples having at least five mutations and sharing at least 50 % of the local variations (missense mutations and insertions and deletions) were filtered and only mutations coming from the most mutation-rich sample were considered to avoid the inclusion of duplicated entries. Altogether, this resulted in the inclusion of 404,913 missense mutations and 9977 in-frame insertions and deletions. Correctly defined mutations were linked to one of the cancer types based on their given sites of occurrence and histology according to the rules given in Additional file 2.

In the COSMIC database mutational data can be mapped to multiple isoforms. The isoform with the largest number of mutations often does not necessarily correspond to the canonic or the main isoform used in other databases. In this work, the isoforms of the same gene were clustered together and the isoform with the the highest number of mutations was selected. For functional and structural annotations and crosslinks to other databases, COSMIC gene ID’s were also mapped to UniProt protein accession codes from the 2016/01 version of UniProt. The entries in each database and the identified regions were mapped to this main isoform, where possible.

### Databases of known cancer related genes

In our study we surveyed four source databases that contained known cancer genes based on expert curation.

#### COSMIC census genes

Apart from mutations and larger scale genetic alterations, the COSMIC database also includes a list of census genes connected to cancer [65]. Somatically altered genes were collected and linked to one or more cancer types. This search yielded 824 gene-cancer type pairs.

#### Driver genes

A set of manually assembled cancer driver genes affected by somatic mutations was taken from [3]. This dataset also grouped genes as either tumor suppressors or oncogenes. The list included 54 oncogenes and 71 tumor suppressor genes.

#### KEGG disease genes

Genes connected to specific types of cancer were collected from the appropriate KEGG Disease pages [66]. For a wide variety of cancers, several genes are quoted together with their dominant type of genetic alterations. Genes are identified with HSA identifiers that are cross-linked with UniProt IDs. Based on this, genes were mapped to COSMIC sequences and the KEGG cancer types were assigned to one of the standardized cancer types providing a list of COSMIC genes with involvement in specific types of cancer. This search yielded 374 gene-cancer type pairs.

#### OMIM cancer genes

*The Online Mendelian Inheritance in Man* provides information about genes with disease association – including cancer [67]. These genes were accessed via UniProt (http://www.uniprot.org/docs/mimtosp) and mapped to COSMIC gene IDs. The referenced OMIM pages were parsed and manually curated. Those pages that described one or more types of cancer were annotated with one or more of the 27 standardized cancer types (see Table 2). This yielded a set of 979 gene-cancer type pairs. In this case, genes affected by somatic or germline mutations could not be generally discriminated.

#### Somatic cancer gene dataset

The number of genes contained in the four datasets containing somatic cancer genes (OMIM, COSMIC census, Drivers and KEGG), together with the amount of overlap between various datasets is shown in the figure in Additional file 4. The fairly limited overlap among the datasets clearly indicates the lack of consensus on cancer genes. The total number of genes that are mentioned in all four datasets is only 32, whereas there are 457 genes annotated as cancer-related that are present only in the OMIM database. In order to reduce the number of falsely annotated genes, only those were considered in the evaluation which occurred in at least two databases of the four. This resulted in 260 cancer genes (referred to as the SCGD (Somatic Cancer Gene Dataset); for an extensive list see table in Additional file 2).

### Identification of significantly mutated protein regions

A novel method was developed to identify significantly mutated protein regions. We refer this method as iSiMPRe (identification of significantly mutated protein regions) and the identified regions as ‘SiMPRe’s. iSiMPRe seeks to find regions that harbor a significantly enriched amount of somatic mutations compared to neutral local mutations which are assumed to be distributed evenly throughout the sequence. To discard a large portion of possible sequencing artefacts, iSiMPRe first filters out mutations that fall into genomic regions with low sequence complexity measured by TRF [68]. Next, the method follows a hierarchical, stepwise algorithm at the determination of possible significant regions, described in detail in Additional file 1 and in short in Fig. 1. Each identified region has a *p*-value assigned that characterizes the significance of the given region based on all three types of mutations. Based on this total *p*-value, regions are classified as high significance (if *p* < 10^−20^), medium significance (10^−20^ < *p* < 10^−05^) or low significance (10^−05^ < *p* < 10^−02^). Within that, however, the dominant mutation type (missense, insertion or deletion) is established for a region based on the relative contribution of each mutation type to the total significance of the region. Here the *p*-values of the found regions were re-calculated with only considering one class of the three possible mutations types. The mutation type yielding the lowest *p*-value on its own was considered to be the dominant one. Furthermore, the resulting regions were assigned to one or more of the 27 cancer types based on the histology and site annotations of the mutations present in the region. These cancer types are shown in Table 2.

### Comparison of iSiMPRe with oncodriveclust and edriver

The performance of our method was compared to two methods with similar scope, OncoDriveClust [16] and e-Driver [17]. For both methods the programs were installed and run locally with the default parameters. For the sake of direct comparison, all three methods were tested on the same set of mutations assembled from COSMIC.

### Association of functional regions with SiMPRes

For each protein in the COSMIC dataset, functional and structural annotations were collected from the corresponding UniProt entry from the UniProt version 2016/01. Additionally, Pfam families, and known instances of linear motif sites collected in the ELM database were also mapped to the studied protein sequences. Altogether 18 categories were assigned to analyze the annotation of the significantly mutated regions (SiMPRe) including domains, motifs, repeats, kinases and enzymes (from Pfam [27]), DNA binding regions, regions of interest, transmembrane regions, nucleotide phosphate binding regions, active sites, binding sites and post-translational modification sites (from UniProt [26]), motifs, switches and phosphorylation sites (from ELM [28] and phosphoELM [69]). The possible over- and under-representation of these functional protein regions in SiMPRes was also determined and tested for statistical significance. For this, a random baseline was established. Each of the 534 found SiMPRe was moved to a randomly selected protein in a way that the length and disorder content of the original and the new, random protein had to be the same within 10 % to avoid structural bias. Next, both real and randomly selected SiMPRes were assessed regarding their overlap with various functional protein units. The random region selection procedure was repeated 1000 times and the average and the standard deviation of the overlap between randomized regions and functional protein regions was calculated. The over-representation of SiMPRe in functional regions compared to randomized regions is given in standard error (StdErr) units in Table 3. As values calculated on random regions closely follow Gaussian distributions, the threshold for *p* < 0.01 statistical significance in StdErr units is 2.326, therefore all color-coded over-representations are statistically significant.

### Assessment of protein disorder

In the process of generating random regions, the disorder content of proteins were assessed using IUPred [70, 71], ANCHOR [72, 73] and Pfam [27]. The output of IUPred was smoothed in a window of 31 residues. Positions with a smoothed IUPred score over 0.5 or with an ANCHOR score over 0.5 were considered disordered, except for positions included in specific Pfam families which were annotated as domains.

### Substitution rates

Substitution rates were calculated from missense mutations from the COSMIC database. Each type of missense mutation was considered only once for each position. First, all types of occurring missense mutations were collected separately for all positions. The number of substitutions changing the original amino acid of type i to type j is denoted by *n*_*i-j*_ and substitution percentages are calculated as follows: *R*_*i-j*_ *= (n*_*i-j*_/N)*100, where N is the total number of missense mutations. These substitution percentages were calculated separately for mutations inside and outside of SiMPRes and are shown in Additional file 2.

## Availability

iSiMPRe is available for download from https://github.com/BalintMeszaros/iSiMPRe.

## Reviewers’ comments

The authors would like to thank the reviewers for the careful reading of our manuscript and their constructive comments. The manuscript was revised in light of their statements. After each of the reviewers’ comments, the authors address each of the comments in detail.

### Reviewer’s report 1: Sándor Pongor, International Centre for Genetic Engineering and Biotechnology (ICGEB), Italy

#### Reviewer comments

##### Reviewer summary

One of the key problems in cancer genomics data is the identification of driver mutations distinction from from passengers mutations. There are a number of known algorithms for the purpose but current approaches are often limited by biases in structural assignments as well as by the inaccuracy of the statistical background models. To stear clear of these problems the authors offer statistically-based method that considers statistically significant missense mutations, in-frame insertions and deletions in a unified statistical framework.

##### Reviewer recommendations to authors

This is a piece of careful work and the results are convincing. One important issue is how an method can be maintained as the databases change and new raw data become available. the authors may want to add a few sentences how their method differs in this respect from other methods.

***Author’s response:*** iSiMPRe is constructed based on a unified statistical model and only a few empirical parameters are present in the method. Furthermore, these parameters (eg. the lengths of the seed regions used, which represent the typical sizes of functional protein regions) are independent of the databases used (eg. COSMIC) and thus the future variations of these databases are not expected to have a significant impact of the performance of iSiMPRe. However, the results, such as the list of potential cancer-related genes are heavily dependent on the input database, and accordingly we developed all our testing protocols to be easily updatable. This makes our workflow extremely capable of conducting follow-up analysis that show e.g. how new cancer genes emerge as a result of the accumulation of sequencing data. The opening paragraph of the Results section was updated to reflect this with the following sentences: *“The input of iSiMPRe is a set of cancer-related missense mutations and in-frame insertions and deletions. The background mutation rate is calculated simply from these two files using only a few empirical parameters. This is in contrast to OncoDriveClust which estimates the background mutation rate from a set of silent substitutions that has to be supplied as a separate set of input data. iSiMPRe is described in detail in Additional file**1**(iSiMPRe protocol) and in short in Fig.*1*. In order to enable potential users to apply the method to updated versions of COSMIC datasets or other sources of cancer mutation data, the source code of iSiMPRe is available for download. In our experience, the identified significantly mutated regions change very little with updates of COSMIC datasets.”*

#### Minor issues

The authors may want to go through the English of text which is however clear and understandable in its present form.

***Author’s response:*** We have made extra care to correct the English of the text.

### Reviewer’s report 2: Michael Gromiha, Indian Institute of Technology Madras, India

#### Reviewer comments

##### Reviewer summary

In this work, the authors presented a novel method for identifying regions that are significantly enriched in somatic mutations and indels. The analysis on human proteome showed the presence of about around 500 protein regions linked with 27 distinct cancer types. It also identified novel genes and regions that have not yet been associated with cancer. The area of research is interesting and potential applications to cancer research. The manuscript is well written and analysis has been made in details.

##### Reviewer recommendations to authors

Following points may be considered for improvements and discussions.

1. Recently, preferred amino acid mutations in cancer genes have been reported using COSMIC database with the location of mutations. A comparison of those results with the present work could be useful.

***Author’s response:*** The substitution rates calculated for the missense mutations inside and outside SiMPRes have been contrasted with the mutation rates detailed in a recent study by Gomiha et al. (Exploring prefered amino acid mutations in cancer genes, applications to identify potential drug targets). Although the two studies use different definitions for driver and passenger mutations (being present in COSMIC multiple times vs being inside a significantly mutated region), the two distributions of preferred amino acid changes show a striking similarity for both drivers and passengers mutations. The cited analysis found 26 driver amino acid substitutions (out of the possible 380) that represent over 1 % of total observed substitutions each. Out of these, the top 7 ones are present in our analysis as well, with 9 more appearing with over 1 % frequency. For the passenger mutations, Gromiha et al. described 29 mutations with over 1 % occurrence, out of which the top 16 (and an additional 10) are present in our list of frequent substitutions as well. The results of the analysis have been included in the supplementary data and have been referred in the discussion section: *“The fundamental differences between passenger and driver mutations can influence the set of preferential amino acid substitutions. In recent analyses, COSMIC mutations in the case of EGFR and also for all genes showed an uneven distribution of missense substitutions among cancer driver mutations defined as reoccurring mutation compared to likely passenger mutations recorded only once in COSMIC. Partitioning driver and passenger mutations based on iSiMPRe showed good agreement with preferential distributions observed earlier (see Additional file**2**), with the 7 and 16 most frequent substitutions appearing in both lists for driver and passenger mutations respectively. These results also hint at the partitioning power of iSiMPRe between driver and passenger missense mutations.”*

2. The preferred localized regions may be discussed in terms of secondary structures and solvent accessibility (either experimental or predicted).

***Author’s response:*** While secondary structure and solvent accessibility properties can be informative for globular proteins, they cannot be directly applied to intrinsically disordered proteins (IDPs). Since a significant portion of the mutated regions can correspond to IDPs, we believe that without partitioning our results based on available structural data and analyzing IDPs and structured protein regions separately, the structural bias would make results difficult to interpret properly. We believe that this structure-based analysis would be really interesting but we also believe that this work is outside the scope of the present paper and will be presented in future publications.

3. COSMIC database provides the counts for the mutants. The number of counts used to define somatic mutations may be mentioned.

***Author’s response:*** These data were added to both the ‘In-frame indels are important for finding cancer genes’ section of Results and the Methods section.

### Reviewer’s report 3: Zoltán Gáspári, Pázmány University, Budapest

#### Reviewer comments

##### Reviewer summary

The manuscript addresses an important problem within a timely topic, namely, the identification of mutational hotspots with biological significance within proteins involved in various forms of cancer. The study is carefully designed and the presentation of the main points of the methodoology and results is generally clear. The iSiMPRE program developed is freely available and the source code is clearly readable. The results provide important novel insights into the role of missense mutations and indels in various proteins and their regions. They can form the basis of further studies on the possible roles of the proteins and functional regions identified in the study.

##### Reviewer recommendations to authors

The field of identifying driver mutations is highly complex. Despite some commonly accepted paradigms there are divergent approaches based on different considerations for the identification of driver genes and/or mutations with biological/therapeutic significance. Therefore, I think that the manuscript could benefit from a theoretical introduction about driver genes and their identification. In particular, it should be important to detail the premises on which the iSiMPRe method is expected to identify driver mutations more efficiently than other methods. This then can be referred in the subsection “The search for cancer driver genes continues” and provide the reader a firm background for the concept of the study. The conclusion section contains some hints, but I suggest that this should be described in a more detailed way in an earlier, suitable part of the paper.

***Author’s response:*** A separate paragraph has been included in the Discussion section to better shed light on the various approaches are used to identify cancer drives genes and the advantages iSiMPRe approach of this work might have over other methods: *“In addition to manually curated databases, various computational methods have also been developed to identify cancer driver genes. Most commonly used approaches seek to identify cancer driver genes either in the context of pathways and protein interaction networks or by detecting signs of positive selection at the level of genes. Such methods can be based on the increased number of observed mutations compared to the background mutation rate; a high rate of non-silent mutations compared to silent mutations; or on the bias towards the accumulation of mutations with high functional impact. However, the accumulation of mutations can highlight not only genes but also specific functional regions at the protein level that are involved in disease development. Recent methods, such as OncoDriveClust or e-Driver identify putative cancer driver genes based on this concept, similarly to the iSiMPre method presented in this work. Clustering of mutations can also be observed in three-dimensional protein structures that often correspond to perturbed protein-protein interaction sites. Given the complexity and heterogeneity of the molecular basis of cancer, the combination of different signals of positive selection can more reliably indicate mutational drivers. Nevertheless, in our experience, the iSiMPre method is able to identify the majority of cancer driver genes based on the clustering of mutations and outperforms methods with similar scope. The increased performance of iSiMPre can be attributed to several factors, including the cleaning of mutational data (e.g. eliminating likely neutral polymorphisms and mutations occurring within tandem repeats that are more likely to accommodate neutral mutations as well as sequencing errors). Additional factor is the incorporation of all genetic variations with positional information, which include short in-frame insertions and deletions while excluding frameshift and non-sense mutations. iSiMPre is based on an unbiased approach that does not rely on previous knowledge of structure or domain, which could be especially important to detect cancer driver mutations located in intrinsically disordered proteins for example (manuscript in preparation).”*

Major recomenndations: In general, the study is well-documented, although there are some details in the methodology that, in my opinion, need to be clarified.

- How were disordered regions identified and how was the extent of disorder assessed for use in the randomization process?

***Author’s response:*** This has been added to the Methods chapter in a separate section.

- Were the categories ‘Kinase’, ‘Enzyme’, ‘Domain’ used in a mutually exclusive way?

***Author’s response:*** No, they are used in accordance with standard Pfam definitions, meaning that all kinases are a subset of enzymes which are in turn a subset of domains.

How exactly were the ‘regions of interest’ defined (which UniProt keywords were included here)?

***Author’s response:*** UniProt contains a ‘regions of interest’ keyword, covering a broad range of functional protein regions. The appropriate section of Results was updated to reflect this.

Which version of UniProt was used?

***Author’s response:*** 2016/01 version, this information has been added to the Methods section as well.

- In all studies using “artificial” thresholds, it could be important to justify the choice of these. Do the authors have data on how inclusion of hypermutated sequences containing more than 100 mutations affect their conclusions? How can the significance regions be justified?

***Author’s response:*** Although no systematic study was done, we calculated the main results with various cutoffs regarding allowed mutations counts per sample. While cutoffs lower than 100 produced apparently poorer results (several known cancer genes were lost from identification), cutoffs between 100 and 150 all yielded approximately the same results. The inclusion of samples with over 150 mutations slowly started to increase the number of identified low significance SiMPRes, however in a cancer type specific way (new regions appeared mainly conjunction with colorectal and lung cancers and melanoma). This indicated that data represented by samples with over 150 mutations contain significantly larger noise. As the region between 100 and 150 seemed to make no difference, we opted to exclude those data as well and used the cutoff of 100 mutations/sample in all final analyses.

Questions/recommendations with respect to the results: -The authors used the isoform with the most mutations. Could the validity of this approach checked at least for the top SiMPRes identified? Do the results obtained for the isoform chosen conform to those that could be obtained for the other isoforms, taking into account their specific role/localization etc.? -

***Author’s response:*** We fully agree that it would be very interesting to study significantly mutated regions in an isoform specific way. However, the current policy of the COSMIC database does not make is possible, as mutations in COSMIC are usually mapped to only one of the isoforms which is often as not the same as the primary isoform.

There is only limited information provided on affected domain types (“Within structured domains, there is a clear tendency for enzymes, especially for kinases, confirming earlier observations”). As the data is at hand, could a more detailed analysis provide some further insights?

***Author’s response:*** We now provide a more detailed table about various structural and functional annotations corresponding to SiMPRes and also added a sentence about the most commonly mutated domains:

*“The most commonly mutated domain is the Tyrosine kinase domain observed in 20 different proteins, including such well-known cancer driver genes as ALK, EGFR or BRAF (see Additional file**3**). Other domains mutated in multiple cases include the Serine/threonine-protein kinase domain (e.g. in Activin receptor type-1 or in Serine/threonine-protein kinase STK11) and the SH2 domains (e.g. in PIK3-R1 and STAT3).”*

Minor questions/recommendations: -

Are there any selected cases where the authors can, with a detailed analysis of the literature, justify that most of the mutations themselves in a SiMPRe indeed significantly affect protein function? - It would be really interesting to analyze in detail one or two cases where different regions of the same protein are associated with different cancer types, but I understand if this is outside of the scope of the present study.

***Author’s response:*** We discuss several examples in the manuscript in some detail, including DNMT3A, PIK3-R1, c-KIT and FGFR3, as well as SMAD4, EGFR and RUNX1 in the discussion. We would also like to emphasize that while most of highly significant mutated regions are well studied examples, for which we have relatively good understanding about the mechanism of the mutation, our understanding for many other cases is still limited. Therefore, we agree with the referee that analyses of further examples are outside of the scope of the present study.

#### Minor issues

Kindly be more specific in statements like “Nevertheless the majority of tumor suppressor genes (50 out of 71,70 %)” as it is bit confusing to (seemingly?) compare numbers to percentages.

***Author’s response:*** These sentences were rephrased to avoid confusion.

I personally would refrain from using terms like “known oncogene” and would prefer “has been described as an oncogene” or similar.

***Author’s response:*** These sentences were re-written to exclude these expressions.

In the sentence “The current collections of genes that have been casually linked to tumor formation” I guess the authors meant “causatively”? Please corrent “genominc” here: “genominc regions with low sequence complexity measured by TRF [62]”

***Author’s response:*** These were corrected.

In Table 4, please add all relevant references weher applicable (e.g. I guess where “Some indication” has been described, there is some kind of reference).

***Author’s response:*** The reference has been added.

Please re-read Additional file 1 and correct typos/small grammatical errors.

***Author’s response:*** We made extra effort to correct typos and small grammatical errors in the Additional file 1.

I suggest to merge all additinal xls files into a single file with multiple tabs.

***Author’s response:*** All additional tables were merged into a single file.

## Additional files

Additional file 1: iSiMPRe protocol. (PDF 415 kb) Additional file 2: All supplementary tables merged. 1: List of all identified SiMPRes. 2: All novel genes that contain identified low significance SiMPRes. 3: Substitution percentages calculated for mutations in and outside of SiMPRes. 4: Criteria for the classification of somatic mutations. 5: The elements of the SCGD dataset with support information. (XLS 285 kb) Additional file 3: iSiMPRe mini-website. The mini-website shows all identified SiMPRes together with the found annotations from various source databases (Pfam, UniProt, ELM, etc.). (ZIP 37 kb) Additional file 4: Cancer driver gene datasets. (PNG 131 kb)

## Abbreviations

AML: acute myeloid leukemia
CHEK2: CHEckpoint kinase 2
CML: chronic myeloid leukemia
COSMIC: catalogue of somatic mutations in cancer
DNMT3A: DNA methyltransferase 3A
EGFR: epidermal growth factor receptor
FGFR3: fibroblast growth factor receptor 3
GIST: gastrointestinal stromal tumor
IL7R: Interleukin-7 receptor
iSiMPRe: identification of Significantly Mutated Protein Regions
KEGG: kyoto encyclopedia of genes and genomes
LOH: loss of heterozygosity
OMIM: online mendelian inheritance in man
PI3K-R1: phosphatidylinositol 3-kinase regulatory subunit alpha
PRC2: polycomb repressive complex 2
SCGD: somatic cancer gene dataset
SiMPRe: significantly mutated protein regions
SNV: single nucleotide variation
STAT3: signal transducer and activator of transcription 3
