# Supplementary material for: Systematic analysis of somatic mutations driving cancer: uncovering functional protein regions in disease development
Source: Biol Direct. 2016 May 5;11:23. doi: 10.1186/s13062-016-0125-6 (PMC4858844; doi:10.1186/s13062-016-0125-6)
Supplement: Additional file 1: — iSiMPRe protocol. (PDF 415 kb) [file 13062_2016_125_MOESM1_ESM.pdf]

iSiMPRe PROTOCOL

The aim of iSiMPRe is to identify regions of a given protein that show a significant over-mutation compared to the rest of the protein. The input parameters, the region optimizing method and the used statistical method are described in the following sections.

1. Input data for iSiMPRe:

The input data of iSiMPRe is a list of local mutations (missense mutations, insertions and deletions) of a protein. Each mutation can be annotated with one cancer type that corresponds to the sample the mutation was sequenced from. In this study, the 27 cancer types listed in Table 1 were used. For each mutation, its location in the sequence has to be specified in the input file (for deletions the location consists of ~~the~~ a start and an end position).
Furthermore, iSiMPRe also expects both the protein and the corresponding cDNA sequences in standard FASTA format as input. This is used to calculate the protein length and to find the low complexity regions of the protein.

2. Region selection and optimization

The algorithm first finds seed regions that contain a statistically significant enrichment of mutations compared to the rest of the protein. In the next step, the boundaries of the seed regions are optimized by extension and amalgamation.

2.1 Seed regions

For the initiation of region identification, first seed regions are defined. Each possible region of length w_1=7 is evaluated for over-representation of mutations (see section 3). Every region that has a p-value of 0.05 or lower is considered a seed region. When a seed region is found, it is checked if it is a sub-region of an already established extended seed region. If not, then the region is extended to maximize significance (minimize p-value), see section 2.2. These extended seed regions are calculated separately ~~for~~ from seed regions of lengths w_1=7, w_2=10 and w_3=30. These lengths were determined empirically and they correspond to typical lengths of functional regions of proteins that are known to be modulated by mutations in cancer. As a last step, these extended seed regions are amalgamated to give the final regions (see section 2.3). First, extended seed regions derived from w_1 and w_2 seed regions are joined, ~~then~~and then the resulting joint regions are further combined with w_3 regions. The p-values of these final regions are calculated and each region is assigned one or more cancer types that reflect the cancer type annotations of the mutations in them. Lastly, for each final region the relative contributions of the three types of mutations (missense, insertion and deletion) to the significance are calculated. The dominant mutation type is the one with the greatest contribution.
The seed region lengths, together with other parameters involved in region amalgamation were determined heuristically checking the algorithm's performance of various biological examples.

2.2 Extending regions

The objective of modifying a region's boundaries is to maximize its significance. This is done in multiple steps. First, the region's right (C terminal) boundary is ~~increased~~ extended as long as ~~while~~ this modification lowers the p-value of the region or until the region's right boundary reaches the end of the protein. Next, this extension process is repeated on the left (N terminal) side as well. If the extension of either side (or both sides) was unsuccessful, then the algorithm~~s~~ checks if the significance improves upon shrinking the region. The shrinking is done in a similar fashion as the extension – moving the boundary is accepted ~~while~~ as long as it lowers the p-value and until the region length doesn't drop to 0. This shrinking is done first on the right side, then on the left.

2.3 Region amalgamation

The joining of extended seed regions coming from two different starting lengths begins by identifying region pairs from the two lists that overlap. Next, iSiMPRe goes through all such pairs and decides on how to optimally combine them. The combination is a function of the amount of overlap and the p-values of the two regions (denoted by A and B). Their common, overlapping parts are always kept in the final joint region~~s~~. However, their unique parts (that do not overlap with the other region) are decided upon based on various factors:

- if the boundaries of the two regions (A and B) coming from different starting lengths are the same after extension (meaning there are no unique parts), then only one is kept.
- if region A contains region B (meaning only A has unique parts) then only one of those is kept. If region A corresponds to a significant extension of B (A is at least 10 residues and at least 25% longer than B), then A is kept if its p-value is at most one order of magnitude higher than that of B (otherwise A is discarded and B is kept). This procedure favors longer regions even at the cost of a slight drop in significance as the resulting region length are more in accordance with biologically relevant lengths. If the two regions have the same p-value, the one with the higher mutation frequency is kept. If neither A or B means a significant extension of the other, then simply the one with the lower p-value is kept.
- if regions A and B only partially overlap, then the amount of overlap is checked (all further descriptions assume that region A comes first; if B comes first all arguments are valid if A and B are interchanged):
    - if the common part is large (more than twice the size of the unique part of both A and B, and both unique parts are less ~~then~~than 10 residues) then A and B are simply merged
    - if the unique part of region B is large (at least 10 residues and larger than half of the common part of A and B), then this unique part is joint to region A (the end point of region A is extended) if for the p-value of the resulting extended region A ($p_{A\text{-extended}}$) the following is true:

$$p_{A-extended} > \frac{1}{2} p_A \frac{L_{B-unique}}{L_{common}}$$

where $L_{B\text{-}unique}$ and L_{common} are the lengths of the unique part of region B and the common part of A and B, respectively. This step allows a small decrease in the significance in favor of region extension, thus favoring longer regions

- if the unique part of region B is small (less than or equal to 10 residues, or smaller than or equal to the half of the common part of A and B), the unique part of B is amalgamated to region A if the p-value of the resulting extended region A is lower than that of the original
- the unique parts of A are subjected to the same criteria as that of B in the decision whether to include them in the final amalgamated region

3. Significance calculations for regions:

For a given region, iSiMPRe quantifies the significance of the over-representation of mutations via calculating p-values (p_{region}). ~~A s~~Separate p-values are calculated for missense mutations (p_{miss}), insertions (p_{ins}) and deletions (p_{del}). These three p-values are obtained by comparing the observed number of mutations in the region to the expected number of mutations calculated based on an underlying statistical model:

$$p_{region}=p_{miss}*p_{ins}*p_{del}$$

The individual terms are calculated as described in the following sections:

3.1 Missense mutations

The underlying assumption behind iSiMPRe is that neutral missense mutations occur randomly along the protein sequence. Therefore, first an expected mutation rate (R_{miss}) is calculated from the $N_{miss,\ total}$ total number of missense mutations in the protein and the L protein length:

$$R_{miss}=N_{miss,total}/L$$

Based on this, for a region with length l the expected number of missense mutations is:

$$N_{miss,exp\_in\_region}=\mathrm{round}(l*R_{miss}).$$

Let N_{miss,obs_in_region} denote the actual number of missense mutations in the selected region. Furthermore, let $N_{miss,obs\_out\_region}=N_{miss,\ total}-N_{miss,obs\_in\_region}$ and $N_{miss,exp\_out\_region}=N_{miss,\ total}-N_{miss,exp\_in\_region}$ be the actual and expected numbers of missense mutations outside of the region, respectively. The quantifiers 'expected' and 'observed'; and 'in region' and 'out region' constitute the two categorical variables and the numbers N_{miss,exp_in_region}, N_{miss,obs_in_region}, N_{miss,exp_out_region} and N_{miss,obs_out_region} are used as input to a one-sider Fisher's exact test to assess the significance of the deviation of the actual distribution of missense mutations (over the binary 'in-region' and 'out-region' classification) compared to the expected evenly random distribution. The output p-value of the Fisher test is termed p_{miss}.

3.2 Insertions

The underlying assumption for insertions is the same as in the case of missense mutations. Furthermore, as insertions can be linked to one position as well, the calculation of the significance of the accumulation of insertions in the region in question in perfectly analogous to the calculations described for missense mutations in 3.1. This calculation yields p_{ins}.

3.3 Deletions

While missense mutations and insertions can be assigned to one definite position in the sequence, this is not true for deletions as they can affect a stretch of residues. Although through a slightly more complicated statistical model, the basic concept is identical to the one used in the calculations for missense mutations and ~~deletions~~insertions;~~:~~ neutral deletions should show an evenly random distribution along the sequence. This, on one hand, means that deletions have no preference along the sequence~~,~~. On the other hand this~~but~~ also means that each deletion~~s~~ is an independent event from the others. Based on this, the total p-value of deletion-enrichment in the given region (p_{del}) can be expressed as:

$$p_{del} = \prod_{i=1}^{k} p_{del,i}$$

where the summation is done for all k deletions in the protein.
For the calculation of the p-values for individual deletions, the expected and ~~observer~~ observed overlaps are calculated. The observed overlap $N_{del,obs\_in\_region,i}$ is simply the number of residues from the region that are covered by the ith deletion and: $N_{del,obs\_out\_region,i}=L_i- N_{del,obs\_in\_region,i}$ where L_i is the length of the deletion. $N_{del,exp\_in\_region,i}$, which is the expected number of overlapping residues between the region and the ith deletion can be calculated by complete enumeration of the possible positions of the deletion. This is given by the following expression:

$$N_{del,exp\_in\_region} = \frac{(|R-L_i|+1)*\min(R,L_i)+\min(R,L_i)^2}{N} + \frac{\min(R,L_i)*(\min(R,L_i)+1)}{N} - corr$$

where N is the length of the protein, R is the length of the region. The first term accounts for cases where the region and the ith deletion overlap completely, that is where one (the longer of the two) contains the other. The second term accounts for cases when there is partial overlap between the region and the deletion. The third term is a correction that should be used in cases when the region is close enough to either the N- or the C-terminal of the protein so that not all possible overlaps between the region and the deletion can occur. For example, if the region boundaries are 5-10 and the length of the ith deletion is 6 residues, the case when the overlap between the region and the deletion is one ~~residues~~residue cannot occur on both sides of the

region, only on the C-terminal side. This correction therefore is only applicable if the region is closer to one of the terminals than the length of the deletion. Using this:

$$corr = \frac{1}{2N}(L_i - x)(L_i - x + 1) \quad \text{if} \quad x < L_i \text{, or:}$$

$$corr = \frac{1}{2N}(L_i - N + x + R - 2)(L_i - N + x + R - 1) \quad \text{if} \quad (N\text{-}x\text{-}R\text{+}2) < L_i$$

where x is the starting position of the region. Similarly to the observed number of deleted positions outside the region, the corresponding expected number can be calculated as: $N_{del,exp\_out\_region,i}$=L_i- $N_{del,exp\_in\_region,i}$. Similarly to the calculations in the case of missense mutations, N_{del,exp_in_region}, N_{del,obs_in_region}, N_{del,exp_out_region} and N_{del,obs_out_region} are used as input to a one-~~sider~~ sided Fisher's exact test, which quantifies the significance of the overlap between the region and the ith deletion, $p^*_{del,i}$.

The above calculated $p^*_{del,i}$ is a function of the protein length, the length of the region, the length of the ith deletion and the overlap between the region and the deletion. Taking biological considerations into account it is clear that when there is only one deletion in the studied protein, p_{del} should not be significant even in the case of the maximal overlap. However, the above described calculations do not guarantee this. As $N_{del,exp\_in\_region,i}$ is proportional to $\frac{1}{N}$, if N>>L_i and N>>R then $N_{del,exp\_in\_region} \cong 0$, independently of all other factors. This means that if the protein ~~in~~ is long enough, the expected overlap between the region and any deletion will be 0. If the region is chosen so that it maximally overlaps with the deletion, $N_{del,obs\_in\_region,i}$ will be high and the overlap will appear as significant ($p^*_{del,i} < 0.01$). To avoid this, all $p^*_{del,i}$ values are re-scaled:

$$p_{del,i} = 0.05\frac{p^*_{del,i}}{p^*_{del,i,max}}$$

where $p^*_{del,i,max}$ is the most significant p-value attainable through the maximal overlap between the region and the ith deletion. This effectively means that irrespective of region, deletion or protein length, the lowest p-value a single deletion can yield is 0.05, keeping it above the 0.01 significance level.

Using this correction, the final combined p-value taking all deletions into consideration is: $p_{del} = \prod_{i=1}^{k} p_{del,i}$.

Formatted: Superscript

Formatted: Font: Italic
