# Supplementary material for: Systematic analysis of somatic mutations driving cancer: uncovering functional protein regions in disease development
Source: Biol Direct. 2016 May 5;11:23. doi: 10.1186/s13062-016-0125-6 (PMC4858844; doi:10.1186/s13062-016-0125-6)
Supplement: Additional file 3: — iSiMPRe mini-website. The mini-website shows all identified SiMPRes together with the found annotations from various source databases (Pfam, UniProt, ELM, etc.). (ZIP 37 kb) [file 13062_2016_125_MOESM3_ESM.zip › indexR1.html]

| Protein Name | Other names | Region | Isoforms | P value | Mutations in region | Total mutations | Pfam domain name | Pfam domain type | Number of annotations | Types of annotations | Heterogeineity index | Dominant cancer tissue type |
| --- | --- | --- | --- | --- | --- | --- | --- | --- | --- | --- | --- | --- |
|  |  |  |  |  |  |  |  |  |  |  |  |  |
| --- | --- | --- | --- | --- | --- | --- | --- | --- | --- | --- | --- | --- |
| ABCC6 | O95255, ABCC6, Multidrug resistance-associated protein 6 | 1139-1171 | - | 0.005682 | 3/0/3/ | 17/0/3/ | ABC\_membrane | Family | 2 | OMIM, | 0.3441 | Pancreatic cancer |
| ABL1 | P00519, ABL1, Tyrosine-protein kinase ABL1 | 242-396 | - | 3.825e-76 | 255/7/66/ | 303/7/182/ | Pkinase\_Tyr | Domain | 12 | Binding\_site, Active\_site, NPB\_site, Uniprot\_motif, PDB, Uniprot\_modification, PhosphoELM\_modification, | 0.1073 | Chronic myeloid leukemia |
| AC097374\_3 | -, -, - | 49-49 | - | 1.861e-05 | 12/0/0/ | 19/0/0/ | Ank\_2 | Repeat | 0 | - | 0.2847 | Prostate cancer, Glioma |
| ACVR1 | Q04771, ACVR1, Activin receptor type-1 | 206-207 | - | 0.0006292 | 10/0/0/ | 56/0/0/ | TGF\_beta\_GS | Family | 3 | PDB, OMIM, | 0.192 | Glioma, Glioblastoma |
| ACVR1 | Q04771, ACVR1, Activin receptor type-1 | 328-328 | - | 3.289e-10 | 26/0/0/ | 56/0/0/ | Pkinase | Domain | 2 | PDB, OMIM, | 0.1845 | Glioma, Glioblastoma |
| ADAMTS2\_ENST00000274609 | O95450-2, ADAMTS2, Isoform SpNPI of A disintegrin and metalloproteinase with thrombospondin motifs 2 | 554-554 | - | 0.007971 | 6/0/0/ | 14/0/0/ |  |  | 0 | - | 0 | Prostate cancer |
| ADAMTS7 | Q9UKP4, ADAMTS7, A disintegrin and metalloproteinase with thrombospondin motifs 7 | 1071-1071 | - | 0.005822 | 7/0/0/ | 39/0/1/ |  |  | 0 | - | 0.3537 | Renal cell carcinoma, Glioma |
| ADAMTSL3 | P82987, ADAMTSL3, ADAMTS-like protein 3 | 938-979 | - | 0.008252 | 9/0/0/ | 59/0/0/ |  |  | 0 | - | 0.2595 | Pancreatic cancer, Ovarian cancer |
| AGAP10 | Q5VTM2, AGAP9, Arf-GAP with GTPase, ANK repeat and PH domain-containing protein 9 | 223-228 | AGAP10\_ENST00000413193|223-228 | 1.01e-05 | 11/0/0/ | 13/0/0/ |  |  | 0 | - | 0.3585 | Prostate cancer |
| AKAP13 | Q12802-2, AKAP13, Isoform 2 of A-kinase anchor protein 13 | 855-859 | - | 0.0025 | 0/0/10/ | 39/0/10/ |  |  | 0 | - | 0 | Renal cell carcinoma |
| AKT1 | P31749, AKT1, RAC-alpha serine/threonine-protein kinase | 17-17 | - | 1.346e-141 | 269/0/0/ | 286/0/0/ | PH | Domain | 4 | OMIM, PDB, Scansite\_motif, Region\_of\_interest, | 0.6091 | Breast cancer |
| ALK | Q9UM73, ALK, ALK tyrosine kinase receptor | 1151-1286 | - | 1.951e-111 | 317/1/0/ | 371/1/0/ | Pkinase\_Tyr | Domain | 30 | PhosphoELM\_modification, Active\_site, Uniprot\_modification, Region\_of\_interest, NPB\_site, OMIM, PDB, Binding\_site, | 0.1505 | Neuroblastoma |
| ALPK2\_ENST00000361673 | Q86TB3, ALPK2, Alpha-protein kinase 2 | 1006-1010 | - | 0.0025 | 0/0/10/ | 41/0/12/ |  |  | 1 | Scansite\_motif, | 0.192 | Pancreatic cancer, Acute myeloid leukemia |
| ALPP | P05187, ALPP, Alkaline phosphatase, placental type | 446-451 | - | 0.007453 | 6/0/0/ | 13/0/0/ | Alk\_phosphatase | Domain | 1 | PDB, | 0.4322 | Pancreatic cancer |
| ANKRD36\_ENST00000420699 | A6QL64, ANKRD36, Ankyrin repeat domain-containing protein 36A | 998-1014 | - | 0.0001802 | 11/0/0/ | 33/0/0/ |  |  | 0 | - | 0.3436 | Renal cell carcinoma, Endometrial cancer |
| APC | P25054, APC, Adenomatous polyposis coli protein | 1278-1537 | - | 1.292e-30 | 157/2/0/ | 323/4/10/ | APC\_crr | Motif | 18 | Scansite\_motif, OMIM, Uniprot\_modification, Region\_of\_interest, PDB, | 0.6355 | Colorectal cancer |
| ARG1 | P05089, ARG1, Arginase-1 | 11-25 | - | 0.004525 | 6/0/0/ | 9/0/0/ | Arginase | Domain | 3 | OMIM, Uniprot\_modification, PDB, | 0.3682 | Lymphoma, Pancreatic cancer |
| ARHGEF17 | Q96PE2, ARHGEF17, Rho guanine nucleotide exchange factor 17 | 232-235 | - | 0.001984 | 1/5/0/ | 24/5/0/ |  |  | 0 | - | 0.4322 | Breast cancer |
| ARID1A | O14497, ARID1A, AT-rich interactive domain-containing protein 1A | 2087-2093 | - | 0.001618 | 9/0/0/ | 100/0/10/ | DUF3518 | Family | 1 | Uniprot\_motif, | 0.4218 | Renal cell carcinoma |
| ARID1B | Q8NFD5-3, ARID1B, Isoform 3 of AT-rich interactive domain-containing protein 1B | 1786-1815 | - | 0.005962 | 3/0/12/ | 33/0/12/ |  |  | 0 | - | 0.1994 | Breast cancer |
| AR | A0A0B4J1T2, AR, Androgen receptor | 871-881 | AR\_ENST00000544984|689-699 | 0.0004125 | 10/0/0/ | 31/0/0/ | Hormone\_recep | Domain | 9 | Region\_of\_interest, OMIM, PDB, Binding\_site, | 0.177 | Prostate cancer |
| ASXL1 | Q8IXJ9, ASXL1, Putative Polycomb group protein ASXL1 | 1102-1107 | - | 8.29e-07 | 19/0/0/ | 112/0/62/ |  |  | 1 | Region\_of\_interest, | 0.2798 | Acute myeloid leukemia |
| ATAD5 | Q96QE3, ATAD5, ATPase family AAA domain-containing protein 5 | 287-292 | - | 6.43e-05 | 1/0/10/ | 10/0/10/ |  |  | 0 | - | 0.4198 | Renal cell carcinoma |
| ATF6B | Q99941, ATF6B, Cyclic AMP-dependent transcription factor ATF-6 beta | 391-394 | - | 0.0025 | 0/0/8/ | 2/0/9/ |  |  | 0 | - | 0 | Pancreatic cancer |
| ATM | Q13315, ATM, Serine-protein kinase ATM | 2014-2023 | - | 0.005601 | 10/0/0/ | 411/3/83/ |  |  | 1 | OMIM, | 0.177 | Lymphoma |
| ATM | Q13315, ATM, Serine-protein kinase ATM | 2437-2453 | ATM\_ENST00000278616|2442-2452 | 2.874e-07 | 28/0/0/ | 411/3/83/ | FAT | Family | 0 | - | 0.4696 | Lymphoma |
| ATM | Q13315, ATM, Serine-protein kinase ATM | 2544-2549 | - | 0.001876 | 1/0/6/ | 411/3/83/ |  |  | 0 | - | 0 | Lymphoma |
| ATM | Q13315, ATM, Serine-protein kinase ATM | 2713-2732 | - | 0.001147 | 17/0/0/ | 411/3/83/ | PI3\_PI4\_kinase | Family | 1 | OMIM, | 0.2188 | Lymphoma |
| ATM | Q13315, ATM, Serine-protein kinase ATM | 2842-2911 | - | 2.089e-05 | 31/0/3/ | 411/3/83/ | PI3\_PI4\_kinase | Family | 5 | OMIM, | 0.4602 | Lymphoma |
| ATM | Q13315, ATM, Serine-protein kinase ATM | 3003-3011 | ATM\_ENST00000278616|3003-3010 | 2.213e-09 | 31/0/1/ | 411/3/83/ |  |  | 1 | OMIM, | 0.2517 | Lymphoma |
| ATM | Q13315, ATM, Serine-protein kinase ATM | 332-340 | - | 6.022e-06 | 16/0/5/ | 411/3/83/ |  |  | 0 | - | 0.4397 | Lymphoma |
| ATM | Q13315, ATM, Serine-protein kinase ATM | 852-858 | - | 0.005601 | 10/0/0/ | 411/3/83/ |  |  | 0 | - | 0.3235 | Other haematopoietic/lymphoid disorder, Lymphoma |
| ATP1A1 | P05023, ATP1A1, Sodium/potassium-transporting ATPase subunit alpha-1 | 99-104 | - | 1.334e-19 | 31/0/22/ | 45/0/25/ | Cation\_ATPase\_N | Domain | 1 | PDB, | 0 | Adrenal tumor |
| ATP1A2 | P50993, ATP1A2, Sodium/potassium-transporting ATPase subunit alpha-2 | 1002-1008 | - | 0.002653 | 8/0/0/ | 40/0/0/ | Cation\_ATPase\_C | Family | 1 | OMIM, | 0.4138 | Colorectal cancer, Pancreatic cancer |
| ATP2B3 | Q16720-2, ATP2B3, Isoform XA of Plasma membrane calcium-transporting ATPase 3 | 422-429 | - | 3.19e-13 | 1/0/33/ | 24/0/33/ | E1-E2\_ATPase | Family | 0 | - | 0.0367 | Adrenal tumor |
| ATRX\_ENST00000373344 | P46100, ATRX, Transcriptional regulator ATRX | 220-224 | - | 0.003372 | 8/0/0/ | 99/0/8/ |  |  | 1 | PDB, | 0.3359 | Glioma, Lymphoma |
| ATRX | P46100, ATRX, Transcriptional regulator ATRX | 1747-1804 | - | 0.0007561 | 12/0/8/ | 133/0/10/ | SNF2\_N | Family | 0 | - | 0.1697 | Glioblastoma |
| ATRX | P46100, ATRX, Transcriptional regulator ATRX | 2136-2217 | ATRX\_ENST00000373344|2144-2217 | 4.004e-05 | 24/0/0/ | 133/0/10/ | Helicase\_C | Family | 2 | Region\_of\_interest, OMIM, | 0.4799 | Glioma, Glioblastoma |
| AXIN1 | O15169, AXIN1, Axin-1 | 72-79 | - | 0.0006819 | 13/0/0/ | 120/0/0/ | AXIN1\_TNKS\_BD | Domain | 4 | Uniprot\_modification, PDB, PhosphoELM\_modification, | 0.3632 | Thyroid cancer, Hepatocellular carcinoma (HCC) |
| B2M | P61769, B2M, Beta-2-microglobulin | 1-8 | - | 0.001947 | 10/0/0/ | 22/0/14/ |  |  | 0 | - | 0 | Lymphoma |
| BAHD1 | Q8TBE0, BAHD1, Bromo adjacent homology domain-containing 1 protein | 653-662 | - | 0.002899 | 7/0/0/ | 14/0/0/ | BAH | Domain | 0 | - | 0.1136 | Ovarian cancer |
| BAP1 | Q92560, BAP1, Ubiquitin carboxyl-terminal hydrolase BAP1 | 1-117 | - | 2.535e-10 | 55/0/20/ | 111/1/34/ | Peptidase\_C12 | Domain | 3 | Scansite\_motif, Active\_site, | 0.4288 | Melanoma, Renal cell carcinoma |
| BAP1 | Q92560, BAP1, Ubiquitin carboxyl-terminal hydrolase BAP1 | 168-173 | - | 8.206e-05 | 16/0/0/ | 111/1/34/ | Peptidase\_C12 | Domain | 1 | Active\_site, | 0.451 | Melanoma |
| BAP1 | Q92560, BAP1, Ubiquitin carboxyl-terminal hydrolase BAP1 | 631-686 | - | 0.0001628 | 7/1/10/ | 111/1/34/ |  |  | 1 | Region\_of\_interest, | 0.2696 | Melanoma |
| BBS1 | Q8NFJ9, BBS1, Bardet-Biedl syndrome 1 protein | 492-494 | ENSG00000256349|529-531 | 0.008837 | 6/0/0/ | 16/0/0/ |  |  | 0 | - | 0.1248 | Ovarian cancer |
| BCL11B | Q9C0K0, BCL11B, B-cell lymphoma/leukemia 11B | 442-479 | - | 0.006471 | 9/0/0/ | 33/0/0/ |  |  | 0 | - | 0.0966 | Lymphoma |
| BCL2\_ENST00000398117 | P10415, BCL2, Apoptosis regulator Bcl-2 | 131-136 | - | 0.004942 | 14/0/0/ | 121/0/0/ | Bcl-2 | Family | 3 | PDB, Scansite\_motif, Uniprot\_motif, | 0.0713 | Lymphoma |
| BCL2 | P10415, BCL2, Apoptosis regulator Bcl-2 | 2-80 | BCL2\_ENST00000398117|2-87 | 7.017e-08 | 92/0/0/ | 142/0/0/ | BH4 | Family | 9 | Uniprot\_motif, PDB, PhosphoELM\_modification, Uniprot\_modification, | 0 | Lymphoma |
| BCL6 | P41182, BCL6, B-cell lymphoma 6 protein | 586-588 | - | 0.0009938 | 9/0/0/ | 31/0/0/ | zf-met | Domain | 1 | PDB, | 0.0966 | Lymphoma |
| BEND5 | Q7L4P6-2, BEND5, Isoform 2 of BEN domain-containing protein 5 | 173-173 | BEND5\_ENST00000371833|342-342 | 0.005418 | 6/0/0/ | 10/0/0/ | BEN | Domain | 0 | - | 0 | Ovarian cancer |
| BIRC3 | Q13489, BIRC3, Baculoviral IAP repeat-containing protein 3 | 555-603 | - | 3.034e-06 | 10/0/25/ | 27/0/25/ | zf-C3HC4\_3 | Domain | 1 | PDB, | 0 | Lymphoma |
| BRAF | P15056, BRAF, Serine/threonine-protein kinase B-raf | 484-490 | - | 0.000125 | 2/0/14/ | 24447/38/112/ | Pkinase\_Tyr | Domain | 2 | OMIM, PDB, | 0.3465 | Ovarian cancer, Non-small cell lung cancer, Lymphoma |
| BRAF | P15056, BRAF, Serine/threonine-protein kinase B-raf | 600-600 | - | 0 | 23941/4/31/ | 24447/38/112/ | Pkinase\_Tyr | Domain | 2 | OMIM, PDB, | 0.3849 | Colorectal cancer, Thyroid cancer, Ovarian cancer, Melanoma, Skin neoplasm, Glioblastoma, Lymphoma |
| BTG1 | P62324, BTG1, Protein BTG1 | 35-38 | - | 0.00249 | 4/0/3/ | 15/0/3/ | BTG | Family | 0 | - | 0 | Lymphoma |
| C10orf18 | Q5VWN6, FAM208B, Protein FAM208B | 1530-1532 | - | 0.0025 | 0/0/6/ | 29/0/6/ |  |  | 0 | - | 0 | Pancreatic cancer |
| C10orf6 | Q8IX21, FAM178A, Protein FAM178A | 795-813 | - | 0.00249 | 4/0/4/ | 15/0/4/ | FAM178 | Family | 0 | - | 0.2879 | Non-small cell lung cancer, Breast cancer, Colorectal cancer |
| C19orf24\_ENST00000409293 | Q9BVV8, C19orf24, Uncharacterized membrane protein C19orf24 | 7-16 | - | 0.0025 | 3/0/5/ | 3/0/5/ |  |  | 0 | - | 0.2493 | Other/various, Non-small cell lung cancer |
| C1orf173 | Q5RHP9, ERICH3, Glutamate-rich protein 3 | 837-848 | - | 0.006402 | 7/0/0/ | 56/0/0/ |  |  | 0 | - | 0.4085 | Prostate cancer |
| C20orf80 | Q9BZ01, FRG1B, Protein FRG1B | 40-101 | FRG1B|40-101  FRG1B\_ENST00000439954|45-51  FRG1B\_ENST00000439954|77-94 | 7.492e-14 | 90/4/0/ | 112/4/0/ | FRG1 | Domain | 1 | PDB, | 0.4742 | Prostate cancer |
| CACNA1D | Q01668-2, CACNA1D, Isoform Beta-cell-type of Voltage-dependent L-type calcium channel subunit alpha-1D | 761-770 | - | 2.705e-06 | 17/0/0/ | 74/0/1/ | Ion\_trans | Family | 0 | - | 0 | Adrenal tumor |
| CACNA1E | Q15878-3, CACNA1E, Isoform 3 of Voltage-dependent R-type calcium channel subunit alpha-1E | 590-595 | - | 0.006848 | 7/0/0/ | 83/0/0/ | Ion\_trans | Family | 0 | - | 0.3744 | Acute myeloid leukemia, Breast cancer, Pancreatic cancer |
| CADM2\_ENST00000383699 | Q8N3J6-2, CADM2, Isoform 2 of Cell adhesion molecule 2 | 319-350 | - | 0.008883 | 8/0/0/ | 18/0/0/ | I-set | Domain | 0 | - | 0.5279 | Acute myeloid leukemia |
| CALCR | A0A0C4DG16, CALCR, Calcitonin receptor | 404-428 | - | 0.003615 | 9/0/0/ | 18/0/0/ |  |  | 0 | - | 0.6085 | - |
| CALR | P27797, CALR, Calreticulin | 365-381 | - | 1.225e-07 | 13/0/35/ | 22/0/35/ |  |  | 1 | Region\_of\_interest, | 0 | Myeloproliferative disease |
| CARD11 | Q9BXL7, CARD11, Caspase recruitment domain-containing protein 11 | 123-134 | - | 0.00132 | 12/0/0/ | 114/1/31/ |  |  | 2 | OMIM, | 0.0794 | Lymphoma |
| CARD11 | Q9BXL7, CARD11, Caspase recruitment domain-containing protein 11 | 191-272 | - | 8.416e-11 | 37/0/31/ | 114/1/31/ |  |  | 0 | - | 0.1208 | Lymphoma |
| CARD11 | Q9BXL7, CARD11, Caspase recruitment domain-containing protein 11 | 353-365 | - | 0.00132 | 12/0/0/ | 114/1/31/ |  |  | 0 | - | 0.1568 | Lymphoma |
| CBL | P22681, CBL, E3 ubiquitin-protein ligase CBL | 367-434 | - | 3.173e-63 | 171/4/723/ | 211/4/767/ | zf-C3HC4\_3 | Domain | 10 | Uniprot\_modification, Region\_of\_interest, OMIM, PhosphoELM\_modification, PDB, | 0.3338 | Other haematopoietic/lymphoid disorder, Acute myeloid leukemia, Chronic myeloid leukemia |
| CCDC136 | Q96JN2-4, CCDC136, Isoform 4 of Coiled-coil domain-containing protein 136 | 662-669 | CCDC136\_ENST00000297788|662-669  NAG6|778-785 | 0.00125 | 1/0/14/ | 16/0/14/ |  |  | 0 | - | 0.0678 | Ovarian cancer |
| CCDC7 | Q96M83, CCDC7, Coiled-coil domain-containing protein 7 | 83-99 | - | 0.006865 | 6/0/0/ | 12/0/0/ | BioT2 | Family | 1 | Scansite\_motif, | 0.1248 | Pancreatic cancer |
| CCND1 | P24385, CCND1, G1/S-specific cyclin-D1 | 36-54 | CCND1\_ENST00000227507|36-54 | 4.236e-12 | 35/0/0/ | 48/0/0/ | Cyclin\_N | Domain | 1 | PDB, | 0 | Lymphoma |
| CCND3 | P30281, CCND3, G1/S-specific cyclin-D3 | 278-290 | - | 1.153e-07 | 18/0/0/ | 22/0/0/ |  |  | 4 | Uniprot\_modification, PhosphoELM\_modification, Scansite\_motif, | 0.1179 | Lymphoma |
| CD209\_ENST00000315599 | Q9NNX6, CD209, CD209 antigen | 129-129 | CD209|129-129  CD209\_ENST00000601951|105-105 | 0.003993 | 7/0/0/ | 19/0/0/ |  |  | 2 | Scansite\_motif, Region\_of\_interest, | 0.2647 | Glioma, Prostate cancer |
| CD74 | P04233-3, CD74, Isoform 3 of HLA class II histocompatibility antigen gamma chain | 1-12 | - | 0.007576 | 5/0/0/ | 6/0/0/ |  |  | 3 | Scansite\_motif, ELM\_instance, | 0.3689 | Lymphoma |
| CD79B | P40259, CD79B, B-cell antigen receptor complex-associated protein beta chain | 191-199 | - | 6.189e-23 | 50/0/0/ | 55/0/0/ |  |  | 4 | PhosphoELM\_modification, ELM\_instance, Uniprot\_modification, | 0.0272 | Lymphoma |
| CDC27 | P30260, CDC27, Cell division cycle protein 27 homolog | 234-274 | CDC27\_ENST00000531206|234-274 | 0.0001325 | 19/0/0/ | 63/0/0/ |  |  | 1 | Uniprot\_modification, | 0.4921 | Prostate cancer |
| CDC27 | P30260, CDC27, Cell division cycle protein 27 homolog | 487-487 | CDC27\_ENST00000531206|493-493 | 0.0001371 | 12/0/0/ | 63/0/0/ |  |  | 1 | PDB, | 0.4521 | Breast cancer, Other soft tissue tumor |
| CDC73 | Q6P1J9, CDC73, Parafibromin | 2-10 | - | 0.005867 | 3/0/6/ | 25/0/12/ | CDC73\_N | Family | 1 | Uniprot\_modification, | 0 | Thyroid cancer |
| CDH1 | P12830, CDH1, Cadherin-1 | 155-309 | - | 4.112e-08 | 48/1/63/ | 139/2/258/ | Cadherin | Family | 3 | PDB, OMIM, Scansite\_motif, | 0.3027 | Stomach cancer, Breast cancer |
| CDH1 | P12830, CDH1, Cadherin-1 | 365-373 | - | 0.004828 | 9/1/9/ | 139/2/258/ | Cadherin | Family | 0 | - | 0.1134 | Stomach cancer |
| CDH1 | P12830, CDH1, Cadherin-1 | 395-408 | - | 0.008643 | 10/0/29/ | 139/2/258/ | Cadherin | Family | 0 | - | 0.2167 | Stomach cancer, Breast cancer |
| CDH7 | Q9ULB5, CDH7, Cadherin-7 | 528-535 | - | 0.006029 | 3/0/3/ | 43/0/3/ | Cadherin | Family | 0 | - | 0.2801 | Lymphoma, Ovarian cancer |
| CDK12 | Q9NYV4, CDK12, Cyclin-dependent kinase 12 | 964-1035 | - | 0.0006401 | 7/0/5/ | 39/0/8/ | Pkinase | Domain | 1 | PDB, | 0.3738 | Ovarian cancer |
| CDKN2A\_ENST00000361570 | Q8N726, CDKN2A, Tumor suppressor ARF | 113-166 | - | 5.766e-16 | 62/0/2/ | 65/0/2/ |  |  | 0 | - | 0.5769 | Pancreatic cancer |
| CDKN2A | P42771, CDKN2A, Cyclin-dependent kinase inhibitor 2A | 16-21 | - | 0.00109 | 13/5/6/ | 476/6/218/ |  |  | 1 | PDB, | 0.5499 | Pancreatic cancer |
| CDKN2A | P42771, CDKN2A, Cyclin-dependent kinase inhibitor 2A | 2-11 | - | 1.25e-05 | 8/1/23/ | 476/6/218/ |  |  | 5 | PhosphoELM\_modification, PDB, Uniprot\_modification, | 0.2949 | Melanoma |
| CDKN2A | P42771, CDKN2A, Cyclin-dependent kinase inhibitor 2A | 28-37 | - | 1.928e-12 | 17/0/64/ | 476/6/218/ |  |  | 3 | PDB, OMIM, | 0.3509 | Squamous cell carcinoma |
| CDKN2A | P42771, CDKN2A, Cyclin-dependent kinase inhibitor 2A | 48-108 | CDKN2A\_ENST00000446177|81-88  CDKN2A\_ENST00000498124|81-87 | 4.229e-18 | 312/0/110/ | 476/6/218/ | Ank\_5 | Domain | 24 | OMIM, PDB, | 0.685 | Squamous cell carcinoma |
| CDR1 | P51861, CDR1, Cerebellar degeneration-related antigen 1 | 210-224 | - | 0.001082 | 6/0/0/ | 6/0/0/ |  |  | 0 | - | 0.3682 | Hepatocellular carcinoma (HCC), Colorectal cancer |
| CDSN | G8JLG2, CDSN, Corneodesmosin | 260-260 | - | 0.004525 | 6/0/0/ | 9/0/3/ |  |  | 0 | - | 0 | Ovarian cancer |
| CEBPA | P49715, CEBPA, CCAAT/enhancer-binding protein alpha | 108-122 | - | 0.00125 | 3/0/38/ | 48/260/480/ |  |  | 0 | - | 0.2105 | Lymphoma, Acute myeloid leukemia |
| CEBPA | P49715, CEBPA, CCAAT/enhancer-binding protein alpha | 14-22 | - | 0.009041 | 4/0/14/ | 48/260/480/ |  |  | 3 | Region\_of\_interest, PhosphoELM\_modification, | 0.0594 | Acute myeloid leukemia |
| CEBPA | P49715, CEBPA, CCAAT/enhancer-binding protein alpha | 293-328 | - | 1.887e-130 | 18/253/118/ | 48/260/480/ | bZIP\_2 | Family | 4 | Region\_of\_interest, PDB, | 0.0139 | Acute myeloid leukemia |
| CHD4\_ENST00000309577 | Q14839-2, CHD4, Isoform 2 of Chromodomain-helicase-DNA-binding protein 4 | 1151-1162 | - | 0.006786 | 6/0/1/ | 56/0/1/ | Helicase\_C | Family | 0 | - | 0.1136 | Endometrial cancer |
| CHD4 | Q14839, CHD4, Chromodomain-helicase-DNA-binding protein 4 | 956-975 | - | 0.002733 | 8/0/0/ | 43/0/0/ | SNF2\_N | Family | 0 | - | 0.5279 | Renal cell carcinoma |
| CHEK2 | O96017, CHEK2, Serine/threonine-protein kinase Chk2 | 372-373 | - | 2.736e-11 | 30/0/0/ | 77/0/0/ | Pkinase | Domain | 2 | PDB, Region\_of\_interest, | 0.5353 | Glioma |
| CHEK2 | O96017, CHEK2, Serine/threonine-protein kinase Chk2 | 519-519 | - | 7.029e-05 | 13/0/0/ | 77/0/0/ |  |  | 0 | - | 0.0751 | Ovarian cancer |
| CHEK2 | O96017, CHEK2, Serine/threonine-protein kinase Chk2 | 535-536 | - | 0.003226 | 8/0/0/ | 77/0/0/ |  |  | 0 | - | 0 | Ovarian cancer |
| CHRNA6 | Q15825, CHRNA6, Neuronal acetylcholine receptor subunit alpha-6 | 8-29 | - | 0.003968 | 5/0/0/ | 5/0/0/ |  |  | 0 | - | 0.3689 | Lymphoma |
| CIC | Q96RK0, CIC, Protein capicua homolog | 1471-1517 | - | 1.042e-06 | 23/0/4/ | 112/0/11/ |  |  | 0 | - | 0.1162 | Glioma |
| CIC | Q96RK0, CIC, Protein capicua homolog | 201-269 | - | 1.292e-18 | 57/0/7/ | 112/0/11/ | HMG\_box | Domain | 0 | - | 0.12 | Glioma |
| CLSTN2 | Q9H4D0, CLSTN2, Calsyntenin-2 | 758-768 | - | 0.005347 | 7/0/0/ | 31/0/0/ |  |  | 0 | - | 0.2647 | Colorectal cancer, Pancreatic cancer |
| CNOT3 | O75175, CNOT3, CCR4-NOT transcription complex subunit 3 | 57-70 | - | 0.0004705 | 12/0/0/ | 28/0/0/ | Not3 | Family | 0 | - | 0.2318 | Lymphoma |
| COPB2 | P35606, COPB2, Coatomer subunit beta' | 825-836 | - | 0.009531 | 6/0/0/ | 18/0/0/ |  |  | 0 | - | 0.4322 | Breast cancer |
| CREBBP | Q92793, CREBBP, CREB-binding protein | 1411-1515 | - | 5.016e-23 | 86/0/1/ | 185/0/18/ | HAT\_KAT11 | Domain | 15 | OMIM, Scansite\_motif, Region\_of\_interest, Binding\_site, | 0.2151 | Lymphoma |
| CREBBP | Q92793, CREBBP, CREB-binding protein | 1680-1689 | - | 0.0002441 | 2/0/11/ | 185/0/18/ |  |  | 1 | Region\_of\_interest, | 0.2198 | Lymphoma |
| CRLF2 | -, -, - | 232-232 | - | 0.0002288 | 9/0/0/ | 13/0/0/ |  |  | 0 | - | 0 | Lymphoma |
| CSF1R | P07333, CSF1R, Macrophage colony-stimulating factor 1 receptor | 301-301 | - | 0.006608 | 7/0/0/ | 66/0/0/ |  |  | 1 | PDB, | 0.1891 | Acute myeloid leukemia, Hepatocellular carcinoma (HCC) |
| CSF1R | P07333, CSF1R, Macrophage colony-stimulating factor 1 receptor | 969-969 | - | 2.908e-08 | 22/0/0/ | 66/0/0/ |  |  | 1 | Uniprot\_modification, | 0.2538 | Acute myeloid leukemia, Chronic myeloid leukemia |
| CSF3R | Q99062-3, CSF3R, Isoform 3 of Granulocyte colony-stimulating factor receptor | 618-618 | - | 4.101e-07 | 17/0/0/ | 32/0/0/ |  |  | 0 | - | 0.2646 | Other haematopoietic/lymphoid disorder |
| CS | O75390, CS, Citrate synthase, mitochondrial | 183-187 | - | 1.67e-06 | 14/0/0/ | 20/0/0/ | Citrate\_synt | Domain | 1 | PDB, | 0.3982 | Bile duct/gallbladder cancer |
| CTNNB1 | P35222, CTNNB1, Catenin beta-1 | 32-45 | - | 0 | 3378/4/1754/ | 3578/4/10035/ |  |  | 24 | Ligand\_switch, ELM\_instance, PDB, PhosphoELM\_modification, Modification\_switch, OMIM, Uniprot\_modification, Scansite\_motif, | 0.5867 | Pancreatic cancer, Colorectal cancer, Stomach cancer, Hepatocellular carcinoma (HCC), Renal cell carcinoma, Endometrial cancer, Other soft tissue tumor, Medulloblastoma, Other/various |
| DBH | P09172, DBH, Dopamine beta-hydroxylase | 270-276 | - | 0.009828 | 6/0/0/ | 19/0/0/ | Cu2\_monooxygen | Domain | 0 | - | 0.3441 | Pancreatic cancer |
| DCAF12L2 | Q5VW00, DCAF12L2, DDB1- and CUL4-associated factor 12-like protein 2 | 334-337 | LOC340578|334-337 | 0.0001866 | 11/0/0/ | 34/0/0/ |  |  | 0 | - | 0.6292 | - |
| DCC | P43146, DCC, Netrin receptor DCC | 1298-1303 | - | 0.00125 | 1/0/6/ | 69/0/6/ | Neogenin\_C | Family | 0 | - | 0.1136 | Lymphoma |
| DCLK1 | O15075-2, DCLK1, Isoform 1 of Serine/threonine-protein kinase DCLK1 | 273-326 | - | 0.009879 | 12/0/0/ | 40/0/0/ |  |  | 0 | - | 0.4521 | Acute myeloid leukemia, Stomach cancer |
| DDX12 | Q96FC9-2, DDX11, Isoform 2 of Probable ATP-dependent RNA helicase DDX11 | 370-370 | - | 0.006865 | 6/0/0/ | 12/3/0/ | DEAD\_2 | Family | 0 | - | 0.4322 | Renal cell carcinoma |
| DDX23 | Q9BUQ8, DDX23, Probable ATP-dependent RNA helicase DDX23 | 345-351 | - | 0.004701 | 7/0/0/ | 24/0/0/ |  |  | 1 | PDB, | 0.1136 | Ovarian cancer |
| DDX3X | O00571, DDX3X, ATP-dependent RNA helicase DDX3X | 302-384 | - | 0.0001924 | 30/0/0/ | 76/0/0/ | DEAD | Domain | 7 | Region\_of\_interest, PhosphoELM\_modification, Scansite\_motif, Uniprot\_motif, PDB, | 0.2458 | Medulloblastoma |
| DDX3X | O00571, DDX3X, ATP-dependent RNA helicase DDX3X | 527-534 | - | 0.004522 | 10/0/0/ | 76/0/0/ | Helicase\_C | Family | 2 | Region\_of\_interest, PDB, | 0.1329 | Medulloblastoma |
| DGKG | P49619, DGKG, Diacylglycerol kinase gamma | 363-378 | - | 0.009531 | 6/0/0/ | 18/0/0/ |  |  | 0 | - | 0.3441 | Breast cancer |
| DHRS4 | Q9BTZ2, DHRS4, Dehydrogenase/reductase SDR family member 4 | 97-102 | - | 0.001935 | 7/0/0/ | 11/0/0/ | adh\_short | Domain | 1 | PDB, | 0.4841 | Medulloblastoma |
| DICER1 | Q9UPY3, DICER1, Endoribonuclease Dicer | 1703-1713 | - | 2.446e-17 | 54/0/0/ | 194/0/1/ | Ribonuclease\_3 | Family | 1 | PDB, | 0.3577 | Ovarian cancer, Other/various |
| DICER1 | Q9UPY3, DICER1, Endoribonuclease Dicer | 1809-1818 | - | 1.448e-37 | 102/0/0/ | 194/0/1/ | Ribonuclease\_3 | Family | 1 | PDB, | 0.395 | Other/various |
| DIS3 | Q9Y2L1, DIS3, Exosome complex exonuclease RRP44 | 479-488 | - | 0.002239 | 8/0/0/ | 29/0/0/ | RNB | Domain | 0 | - | 0.3839 | Acute myeloid leukemia |
| DKK2 | Q9UBU2, DKK2, Dickkopf-related protein 2 | 222-230 | - | 0.009828 | 8/0/0/ | 20/0/0/ |  |  | 2 | PDB, Region\_of\_interest, | 0.4799 | Neuroblastoma, Colorectal cancer |
| DNAH3\_ENST00000261383 | Q8TD57, DNAH3, Dynein heavy chain 3, axonemal | 3336-3361 | - | 0.006801 | 7/0/0/ | 79/0/0/ |  |  | 0 | - | 0.3537 | Hepatocellular carcinoma (HCC), Renal cell carcinoma |
| DNM2 | P50570, DNM2, Dynamin-2 | 354-364 | - | 0.005773 | 7/0/0/ | 38/0/1/ | Dynamin\_M | Family | 1 | OMIM, | 0.1136 | Lymphoma |
| DNM2 | P50570, DNM2, Dynamin-2 | 653-658 | DNM2\_ENST00000389253|653-658 | 0.002594 | 8/0/0/ | 38/0/1/ | GED | Family | 0 | - | 0.1043 | Lymphoma |
| DNMT3A | Q9Y6K1, DNMT3A, DNA (cytosine-5)-methyltransferase 3A | 535-583 | - | 1.323e-05 | 40/2/11/ | 994/6/42/ |  |  | 4 | PDB, Region\_of\_interest, OMIM, | 0.17 | Acute myeloid leukemia |
| DNMT3A | Q9Y6K1, DNMT3A, DNA (cytosine-5)-methyltransferase 3A | 729-737 | DNMT3A\_ENST00000380746|540-547 | 1.854e-08 | 40/0/9/ | 994/6/42/ | DNA\_methylase | Domain | 1 | PDB, | 0.2547 | Acute myeloid leukemia, Lymphoma |
| DNMT3A | Q9Y6K1, DNMT3A, DNA (cytosine-5)-methyltransferase 3A | 751-762 | - | 0.006053 | 10/4/2/ | 994/6/42/ | DNA\_methylase | Domain | 1 | PDB, | 0.2441 | Lymphoma, Acute myeloid leukemia |
| DNMT3A | Q9Y6K1, DNMT3A, DNA (cytosine-5)-methyltransferase 3A | 882-882 | DNMT3A\_ENST00000380746|693-697 | 0 | 675/0/0/ | 994/6/42/ |  |  | 1 | PDB, | 0.0945 | Acute myeloid leukemia |
| DPY19L2 | Q6NUT2, DPY19L2, Probable C-mannosyltransferase DPY19L2 | 647-647 | - | 0.00445 | 7/0/0/ | 22/0/0/ | Dpy19 | Family | 0 | - | 0.2647 | Prostate cancer, Pancreatic cancer |
| DSCAML1 | Q8TD84, DSCAML1, Down syndrome cell adhesion molecule-like protein 1 | 766-823 | - | 0.001184 | 14/0/0/ | 67/0/0/ | I-set | Domain | 0 | - | 0.5353 | - |
| DYRK4 | -, -, - | 578-586 | - | 0.009202 | 6/0/0/ | 17/0/0/ | Pkinase | Domain | 0 | - | 0.2403 | Non-small cell lung cancer |
| EBPL | Q9BY08, EBPL, Emopamil-binding protein-like | 189-189 | - | 0.000673 | 8/0/0/ | 12/0/0/ | EBP | Family | 0 | - | 0.4319 | Endometrial cancer, Renal cell carcinoma, Pancreatic cancer |
| EEF1B2 | P24534, EEF1B2, Elongation factor 1-beta | 43-43 | - | 3.739e-07 | 13/0/0/ | 14/0/0/ |  |  | 0 | - | 0.3264 | Prostate cancer |
| EFCAB6 | Q5THR3, EFCAB6, EF-hand calcium-binding domain-containing protein 6 | 412-420 | - | 0.005867 | 3/0/5/ | 25/0/5/ | EF-hand\_8 | Domain | 0 | - | 0.2973 | Squamous cell carcinoma |
| EGFR | P00533, EGFR, Epidermal growth factor receptor | 694-810 | - | 0 | 770/172/11068/ | 3445/172/11068/ | Pkinase\_Tyr | Domain | 12 | PDB, Uniprot\_modification, NPB\_site, Scansite\_motif, Region\_of\_interest, Binding\_site, PhosphoELM\_modification, | 0.0643 | Squamous cell carcinoma, Non-small cell lung cancer |
| EGFR | P00533, EGFR, Epidermal growth factor receptor | 831-839 | - | 1.056e-06 | 73/0/0/ | 3445/172/11068/ | Pkinase\_Tyr | Domain | 2 | PDB, Active\_site, | 0.3636 | Non-small cell lung cancer |
| EGFR | P00533, EGFR, Epidermal growth factor receptor | 858-858 | - | 0 | 2227/0/0/ | 3445/172/11068/ | Pkinase\_Tyr | Domain | 1 | PDB, | 0.0526 | Non-small cell lung cancer |
| EGFR | P00533, EGFR, Epidermal growth factor receptor | 861-864 | - | 3.415e-20 | 102/0/0/ | 3445/172/11068/ | Pkinase\_Tyr | Domain | 1 | PDB, | 0.1536 | Non-small cell lung cancer |
| EIF1AX | P47813, EIF1AX, Eukaryotic translation initiation factor 1A, X-chromosomal | 2-15 | - | 1.457e-06 | 14/0/0/ | 15/0/0/ |  |  | 1 | PDB, | 0.3811 | Thyroid cancer, Melanoma |
| ENGASE | Q8NFI3, ENGASE, Cytosolic endo-beta-N-acetylglucosaminidase | 73-114 | - | 0.001923 | 4/0/6/ | 8/0/6/ |  |  | 0 | - | 0.3016 | Breast cancer |
| ENPP7 | Q6UWV6, ENPP7, Ectonucleotide pyrophosphatase/phosphodiesterase family member 7 | 373-376 | - | 0.007453 | 6/0/0/ | 13/0/0/ |  |  | 0 | - | 0.3441 | Prostate cancer |
| ENSG00000103472 | -, -, - | 368-375 | - | 1.676e-09 | 18/0/0/ | 20/0/0/ | RRN3 | Family | 0 | - | 0.1542 | Prostate cancer |
| ENSG00000164845 | -, -, - | 43-48 | - | 0.007576 | 5/0/0/ | 6/0/0/ | FAM86 | Family | 0 | - | 0.3689 | Glioma |
| ENSG00000174501 | Q5JPF3, ANKRD36C, Ankyrin repeat domain-containing protein 36C | 626-634 | - | 2.62e-12 | 31/0/0/ | 61/0/0/ |  |  | 0 | - | 0.2156 | Prostate cancer, Glioma |
| EP300 | Q09472, EP300, Histone acetyltransferase p300 | 1372-1514 | - | 1.332e-11 | 54/0/2/ | 155/2/93/ | HAT\_KAT11 | Domain | 10 | Binding\_site, Region\_of\_interest, PDB, Uniprot\_modification, Scansite\_motif, | 0.3964 | Squamous cell carcinoma |
| EP300 | Q09472, EP300, Histone acetyltransferase p300 | 2175-2221 | - | 1.567e-06 | 6/0/19/ | 155/2/93/ |  |  | 2 | Region\_of\_interest, | 0.2333 | Lymphoma, Acute myeloid leukemia |
| EP300 | Q09472, EP300, Histone acetyltransferase p300 | 2268-2268 | - | 0.0004883 | 1/0/10/ | 155/2/93/ |  |  | 0 | - | 0.0844 | Acute myeloid leukemia |
| EPHA10 | Q5JZY3, EPHA10, Ephrin type-A receptor 10 | 150-157 | - | 0.004813 | 7/0/0/ | 25/0/0/ | Ephrin\_lbd | Domain | 0 | - | 0.4841 | Stomach cancer |
| EPHA5 | P54756, EPHA5, Ephrin type-A receptor 5 | 1032-1037 | - | 0.00659 | 7/0/0/ | 65/0/0/ |  |  | 1 | Uniprot\_motif, | 0.2647 | Non-small cell lung cancer, Hepatocellular carcinoma (HCC) |
| ERBB2 | P04626, ERBB2, Receptor tyrosine-protein kinase erbB-2 | 309-310 | - | 9.658e-06 | 16/0/0/ | 139/85/6/ | Furin-like | Domain | 1 | PDB, | 0.5235 | Breast cancer |
| ERBB2 | P04626, ERBB2, Receptor tyrosine-protein kinase erbB-2 | 733-799 | - | 2.816e-57 | 57/85/6/ | 139/85/6/ | Pkinase\_Tyr | Domain | 4 | PhosphoELM\_modification, PDB, NPB\_site, Binding\_site, | 0.3937 | Non-small cell lung cancer |
| ERBB2 | P04626, ERBB2, Receptor tyrosine-protein kinase erbB-2 | 821-914 | - | 0.0008029 | 29/0/0/ | 139/85/6/ | Pkinase\_Tyr | Domain | 3 | Active\_site, PDB, PhosphoELM\_modification, | 0.6643 | - |
| ERBB3\_ENST00000267101 | P21860, ERBB3, Receptor tyrosine-protein kinase erbB-3 | 101-104 | ERBB3|101-104  ERBB3\_ENST00000411731|101-104 | 0.003045 | 8/0/0/ | 60/0/0/ | Recep\_L\_domain | Domain | 1 | PDB, | 0.3119 | Bile duct/gallbladder cancer |
| ESR1 | P03372, ESR1, Estrogen receptor | 303-303 | - | 3.24e-91 | 206/0/0/ | 275/1/2/ |  |  | 6 | Scansite\_motif, Region\_of\_interest, PDB, | 0 | Breast cancer |
| ESR1 | P03372, ESR1, Estrogen receptor | 534-538 | - | 1.327e-12 | 45/0/0/ | 275/1/2/ |  |  | 9 | PhosphoELM\_modification, Region\_of\_interest, PDB, Uniprot\_modification, | 0.0295 | Breast cancer |
| ETNK1 | Q9HBU6, ETNK1, Ethanolamine kinase 1 | 243-245 | - | 8.705e-11 | 23/0/0/ | 30/0/2/ | Choline\_kinase | Family | 0 | - | 0.2498 | Chronic myeloid leukemia, Other haematopoietic/lymphoid disorder |
| ETV6 | P41212, ETV6, Transcription factor ETV6 | 347-403 | - | 0.005574 | 14/0/0/ | 32/2/2/ | Ets | Domain | 3 | PDB, OMIM, | 0.3968 | Acute myeloid leukemia, Lymphoma |
| ETV6 | P41212, ETV6, Transcription factor ETV6 | 97-110 | - | 0.004085 | 5/2/2/ | 32/2/2/ | SAM\_PNT | Domain | 1 | PDB, | 0.3364 | Acute myeloid leukemia, Lymphoma |
| EZH2 | Q15910-2, EZH2, Isoform 2 of Histone-lysine N-methyltransferase EZH2 | 636-695 | EZH2\_ENST00000350995|582-676 | 2.017e-82 | 265/0/2/ | 350/4/2/ | SET | Family | 2 | OMIM, PDB, | 0.252 | Lymphoma |
| FAM174B | Q3ZCQ3, FAM174B, Membrane protein FAM174B | 69-70 | - | 0.00463 | 0/0/6/ | 2/0/6/ | DUF1180 | Family | 0 | - | 0.1763 | Breast cancer, Melanoma |
| FAM5C | Q76B58, BRINP3, BMP/retinoic acid-inducible neural-specific protein 3 | 116-119 | - | 0.006626 | 7/0/0/ | 67/0/0/ | MACPF | Domain | 2 | Scansite\_motif, | 0.3195 | Acute myeloid leukemia |
| FBXW7 | Q969H0, FBXW7, F-box/WD repeat-containing protein 7 | 11-15 | FBXW7\_ENST00000281708|15-15 | 0.001015 | 1/6/0/ | 396/6/5/ |  |  | 6 | Scansite\_motif, Ligand\_switch, ELM\_instance, | 0.2205 | Acute myeloid leukemia |
| FBXW7 | Q969H0, FBXW7, F-box/WD repeat-containing protein 7 | 420-423 | - | 0.003434 | 13/0/0/ | 396/6/5/ | WD40 | Repeat | 1 | PDB, | 0.3611 | Lymphoma |
| FBXW7 | Q969H0, FBXW7, F-box/WD repeat-containing protein 7 | 437-532 | FBXW7\_ENST00000281708|438-514  FBXW7\_ENST00000296555|319-396  FBXW7\_ENST00000534231|198-266  FBXW7\_NM\_018315\_2|358-439 | 4.915e-75 | 301/0/0/ | 396/6/5/ | WD40 | Repeat | 1 | PDB, | 0.454 | Lymphoma |
| FBXW7 | Q969H0, FBXW7, F-box/WD repeat-containing protein 7 | 579-582 | - | 9.583e-05 | 14/0/3/ | 396/6/5/ | WD40 | Repeat | 1 | PDB, | 0.1678 | Breast cancer, Colorectal cancer |
| FBXW7 | Q969H0, FBXW7, F-box/WD repeat-containing protein 7 | 689-689 | - | 0.0004413 | 14/0/0/ | 396/6/5/ |  |  | 1 | PDB, | 0.3688 | Lymphoma |
| FCAMR\_ENST00000324852 | A0A0B4J1S2, FCAMR, High affinity immunoglobulin alpha and immunoglobulin mu Fc receptor | 88-88 | FCAMR|88-88  FCAMR\_ENST00000367087|64-64 | 0.004525 | 6/0/0/ | 9/0/0/ |  |  | 0 | - | 0 | Ovarian cancer |
| FEM1A | Q9BSK4, FEM1A, Protein fem-1 homolog A | 620-620 | - | 0.002609 | 7/0/0/ | 13/0/0/ | Ank\_5 | Domain | 0 | - | 0.2988 | Pancreatic cancer, Glioma, Prostate cancer |
| FGFR1 | P11362, FGFR1, Fibroblast growth factor receptor 1 | 546-547 | FGFR1\_ENST00000335922|536-537  FGFR1\_ENST00000341462|546-546  FGFR1\_ENST00000447712|546-547  Q7Z2S2\_HUMAN|546-547 | 3.532e-06 | 16/0/0/ | 49/0/0/ | Pkinase\_Tyr | Domain | 1 | PDB, | 0.4755 | Glioblastoma, Medulloblastoma |
| FGFR1 | P11362, FGFR1, Fibroblast growth factor receptor 1 | 655-664 | - | 0.0004151 | 13/0/0/ | 49/0/0/ | Pkinase\_Tyr | Domain | 1 | PDB, | 0.4445 | Non-small cell lung cancer, Medulloblastoma |
| FGFR2 | P21802, FGFR2, Fibroblast growth factor receptor 2 | 248-253 | FGFR2\_ENST00000351936|252-253  FGFR2\_ENST00000357555|163-164  FGFR2\_ENST00000457416|252-253 | 8.489e-15 | 46/0/0/ | 158/0/6/ | I-set | Domain | 3 | OMIM, PDB, | 0.1353 | Endometrial cancer |
| FGFR2 | P21802, FGFR2, Fibroblast growth factor receptor 2 | 370-397 | - | 0.0001196 | 19/0/5/ | 158/0/6/ |  |  | 5 | OMIM, | 0.396 | Other/various, Endometrial cancer |
| FGFR2 | P21802, FGFR2, Fibroblast growth factor receptor 2 | 544-549 | FGFR2\_ENST00000351936|547-547  FGFR2\_ENST00000357555|460-460  FGFR2\_ENST00000369056|550-550  FGFR2\_ENST00000457416|550-550 | 7.68e-09 | 29/0/0/ | 158/0/6/ | Pkinase\_Tyr | Domain | 2 | OMIM, PDB, | 0.3271 | Endometrial cancer |
| FGFR2 | P21802, FGFR2, Fibroblast growth factor receptor 2 | 659-659 | - | 0.0073 | 7/0/0/ | 158/0/6/ | Pkinase\_Tyr | Domain | 1 | PDB, | 0.3195 | Endometrial cancer |
| FGFR3 | P22607, FGFR3, Fibroblast growth factor receptor 3 | 249-249 | FGFR3\_ENST00000340107|248-249 | 0 | 1301/0/1/ | 2374/3/2/ |  |  | 2 | PDB, OMIM, | 0.0693 | Bladder cancer |
| FGFR3 | P22607, FGFR3, Fibroblast growth factor receptor 3 | 370-380 | FGFR3\_ENST00000340107|372-375 | 3.087e-157 | 609/0/0/ | 2374/3/2/ |  |  | 6 | PDB, OMIM, | 0.1184 | Bladder cancer |
| FGFR3 | P22607, FGFR3, Fibroblast growth factor receptor 3 | 391-391 | - | 1.612e-08 | 36/0/0/ | 2374/3/2/ |  |  | 2 | OMIM, PDB, | 0.0941 | Bladder cancer |
| FGFR3 | P22607, FGFR3, Fibroblast growth factor receptor 3 | 650-650 | - | 1.017e-25 | 96/0/0/ | 2374/3/2/ | Pkinase\_Tyr | Domain | 2 | OMIM, PDB, | 0.2913 | Skin neoplasm, Bladder cancer |
| FGFR3 | P22607, FGFR3, Fibroblast growth factor receptor 3 | 697-697 | - | 1.035e-10 | 44/0/0/ | 2374/3/2/ | Pkinase\_Tyr | Domain | 1 | PDB, | 0 | Squamous cell carcinoma |
| FLJ42177 | Q5TCS8-5, AK9, Isoform 5 of Adenylate kinase 9 | 295-295 | - | 0.003906 | 0/0/8/ | 6/0/8/ |  |  | 1 | Scansite\_motif, | 0 | Breast cancer |
| FLT3 | P36888, FLT3, Receptor-type tyrosine-protein kinase FLT3 | 573-627 | - | 1.364e-87 | 18/175/25/ | 431/181/76/ | Pkinase\_Tyr | Domain | 14 | Uniprot\_modification, Region\_of\_interest, PhosphoELM\_modification, Scansite\_motif, NPB\_site, PDB, | 0.1448 | Acute myeloid leukemia |
| FLT3 | P36888, FLT3, Receptor-type tyrosine-protein kinase FLT3 | 831-842 | - | 1.7e-163 | 345/3/47/ | 431/181/76/ | Pkinase\_Tyr | Domain | 3 | PDB, Uniprot\_modification, PhosphoELM\_modification, | 0.1236 | Acute myeloid leukemia |
| FNBP4 | Q8N3X1, FNBP4, Formin-binding protein 4 | 58-59 | - | 2.143e-05 | 0/0/12/ | 12/0/13/ |  |  | 0 | - | 0.4322 | Melanoma |
| FOXA1 | P55317, FOXA1, Hepatocyte nuclear factor 3-alpha | 226-266 | - | 4.084e-07 | 23/0/0/ | 34/0/0/ | Forkhead | Domain | 1 | PDB, | 0.145 | Prostate cancer |
| FOXL2 | P58012, FOXL2, Forkhead box protein L2 | 134-134 | - | 6.813e-239 | 418/0/0/ | 424/0/0/ | Forkhead | Domain | 0 | - | 0.0047 | Ovarian cancer |
| FOXO1 | Q12778, FOXO1, Forkhead box protein O1 | 19-24 | FOXO1\_ENST00000379561|19-24 | 7.265e-05 | 11/0/0/ | 20/0/0/ |  |  | 6 | Scansite\_motif, PhosphoELM\_modification, Uniprot\_modification, | 0.1662 | Lymphoma |
| FRG1 | Q14331, FRG1, Protein FRG1 | 125-163 | - | 0.0004091 | 17/0/0/ | 29/0/2/ | FRG1 | Domain | 1 | PDB, | 0.4514 | Breast cancer |
| FRG1 | Q14331, FRG1, Protein FRG1 | 86-98 | - | 0.006463 | 7/0/2/ | 29/0/2/ | FRG1 | Domain | 1 | PDB, | 0.4644 | Lymphoma |
| FRK | P42685, FRK, Tyrosine-protein kinase FRK | 378-388 | - | 0.0002083 | 1/0/8/ | 10/0/8/ | Pkinase\_Tyr | Domain | 2 | Uniprot\_modification, PhosphoELM\_modification, | 0 | Hepatocellular carcinoma (HCC) |
| FTMT | Q8N4E7, FTMT, Ferritin, mitochondrial | 34-69 | - | 0.0003395 | 12/0/0/ | 15/0/0/ |  |  | 2 | Scansite\_motif, PDB, | 0.4705 | Pancreatic cancer |
| GATA1 | P15976, GATA1, Erythroid transcription factor | 1-2 | - | 5.756e-06 | 15/0/0/ | 39/1/194/ |  |  | 0 | - | 0.1864 | Acute myeloid leukemia, Myeloproliferative disease |
| GATA1 | P15976, GATA1, Erythroid transcription factor | 71-74 | - | 2.526e-05 | 10/0/9/ | 39/1/194/ |  |  | 1 | Uniprot\_modification, | 0.1823 | Acute myeloid leukemia, Myeloproliferative disease |
| GATA2 | P23769, GATA2, Endothelial transcription factor GATA-2 | 293-390 | - | 5.311e-24 | 78/2/9/ | 86/2/9/ | GATA | Domain | 7 | OMIM, PDB, Scansite\_motif, | 0.2079 | Acute myeloid leukemia |
| GATA3 | P23771-2, GATA3, Isoform 2 of Trans-acting T-cell-specific transcription factor GATA-3 | 276-276 | - | 0.005105 | 7/0/0/ | 28/1/2/ | GATA | Domain | 1 | PDB, | 0 | Lymphoma |
| GLTPD2 | A6NH11, GLTPD2, Glycolipid transfer protein domain-containing protein 2 | 115-115 | - | 0.007576 | 5/0/0/ | 6/0/0/ | GLTP | Domain | 0 | - | 0 | Renal cell carcinoma |
| GNA11 | P29992, GNA11, Guanine nucleotide-binding protein subunit alpha-11 | 209-209 | - | 5.172e-83 | 162/0/0/ | 179/0/0/ | G-alpha | Domain | 2 | NPB\_site, PDB, | 0.144 | Melanoma |
| GNAI2 | P04899, GNAI2, Guanine nucleotide-binding protein G(i) subunit alpha-2 | 179-186 | - | 0.0004635 | 9/0/0/ | 17/0/0/ | G-alpha | Domain | 2 | NPB\_site, Uniprot\_modification, | 0.3182 | Lymphoma |
| GNAQ | P50148, GNAQ, Guanine nucleotide-binding protein G(q) subunit alpha | 209-211 | - | 1.484e-136 | 265/0/0/ | 283/0/0/ | G-alpha | Domain | 2 | PDB, NPB\_site, | 0.2539 | Melanoma, Skin neoplasm |
| GNAS | P63092, GNAS, Guanine nucleotide-binding protein G(s) subunit alpha isoforms short | 201-201 | GNAS\_ENST00000371100|842-876 | 0 | 868/0/0/ | 925/0/0/ | G-alpha | Domain | 4 | NPB\_site, OMIM, PDB, Uniprot\_modification, | 0.5403 | Pancreatic cancer, Bone cancer, Other/various |
| GNAS | P63092, GNAS, Guanine nucleotide-binding protein G(s) subunit alpha isoforms short | 227-227 | - | 7.377e-09 | 34/0/0/ | 925/0/0/ | G-alpha | Domain | 2 | PDB, NPB\_site, | 0.3271 | Thyroid cancer, Other/various |
| GPR111 | Q8IZF7-2, GPR111, Isoform 2 of Probable G-protein coupled receptor 111 | 36-38 | GPR111\_ENST00000296862|104-106 | 0.0025 | 0/0/6/ | 3/0/6/ |  |  | 0 | - | 0 | Pancreatic cancer |
| GPR32 | O75388, GPR32, Probable G-protein coupled receptor 32 | 332-336 | - | 0.003615 | 7/0/0/ | 17/0/0/ |  |  | 0 | - | 0.3744 | Breast cancer, Pancreatic cancer, Ovarian cancer |
| GPR50\_ENST00000218316 | Q13585, GPR50, Melatonin-related receptor | 502-505 | - | 0.00125 | 1/0/8/ | 21/0/8/ |  |  | 0 | - | 0.2672 | Pancreatic cancer, Ovarian cancer |
| GPR98 | Q8WXG9, GPR98, G-protein coupled receptor 98 | 4495-4504 | - | 0.003522 | 8/0/0/ | 139/0/0/ |  |  | 0 | - | 0.3359 | Hepatocellular carcinoma (HCC), Pancreatic cancer |
| GPX1 | P07203, GPX1, Glutathione peroxidase 1 | 77-77 | - | 0.003968 | 5/0/0/ | 5/0/0/ | GSHPx | Family | 1 | PDB, | 0.3689 | Prostate cancer |
| GRM1 | Q13255, GRM1, Metabotropic glutamate receptor 1 | 681-688 | - | 0.006402 | 7/0/0/ | 56/0/0/ | 7tm\_3 | Family | 1 | PDB, | 0.5389 | - |
| GUCY1A2 | P33402, GUCY1A2, Guanylate cyclase soluble subunit alpha-2 | 652-723 | - | 8.362e-05 | 18/0/0/ | 35/0/0/ | Guanylate\_cyc | Domain | 0 | - | 0.5417 | - |
| GXYLT1 | Q4G148, GXYLT1, Glucoside xylosyltransferase 1 | 408-414 | Q8IXV1\_HUMAN|377-383 | 0.002015 | 8/0/0/ | 25/0/0/ |  |  | 0 | - | 0.1832 | Hepatocellular carcinoma (HCC), Prostate cancer |
| H2AFV | Q71UI9, H2AFV, Histone H2A.V | 119-119 | - | 0.002331 | 6/0/0/ | 7/0/0/ | Histone\_H2A\_C | Family | 1 | PDB, | 0.2403 | Pancreatic cancer |
| H3F3A | P84243, H3F3A, Histone H3.3 | 28-35 | - | 2.556e-136 | 283/0/0/ | 287/0/0/ | Histone | Domain | 23 | Uniprot\_modification, PDB, PhosphoELM\_modification, | 0.176 | Glioblastoma |
| H3F3B | P84243, H3F3A, Histone H3.3 | 35-37 | - | 0.007576 | 5/0/0/ | 6/0/0/ | Histone | Domain | 9 | PDB, Uniprot\_modification, | 0.1386 | Bone cancer |
| HCN1 | O60741, HCN1, Potassium/sodium hyperpolarization-activated cyclic nucleotide-gated channel 1 | 267-268 | - | 0.00624 | 7/0/0/ | 50/0/0/ | Ion\_trans | Family | 0 | - | 0.3195 | Pancreatic cancer |
| HDAC9 | Q9UKV0-7, HDAC9, Isoform 7 of Histone deacetylase 9 | 586-586 | HDAC9\_ENST00000262069|586-586 | 0.0001802 | 11/0/0/ | 33/0/0/ |  |  | 0 | - | 0 | Ovarian cancer |
| HERC2P3 | Q9BVR0, HERC2P3, Putative HERC2-like protein 3 | 795-815 | - | 0.002899 | 7/0/0/ | 14/0/0/ |  |  | 0 | - | 0.3744 | Prostate cancer, Glioma, Endometrial cancer |
| HERC2 | O95714, HERC2, E3 ubiquitin-protein ligase HERC2 | 1198-1230 | HERC2\_ENST00000261609|1198-1211 | 0.001177 | 12/0/0/ | 81/0/0/ | Cyt-b5 | Domain | 0 | - | 0.5801 | Renal cell carcinoma |
| HIST1H3B | P68431, HIST1H3A, Histone H3.1 | 28-28 | - | 1.038e-16 | 37/0/0/ | 50/0/0/ | Histone | Domain | 6 | PDB, Uniprot\_modification, | 0.2345 | Glioma, Glioblastoma |
| HNF1A | A0A0A0MQU7, HNF1A, Hepatocyte nuclear factor 1-alpha | 1-30 | - | 0.0001118 | 2/0/14/ | 88/7/30/ | HNF-1\_N | Family | 4 | OMIM, Region\_of\_interest, PDB, | 0 | Hepatocellular carcinoma (HCC) |
| HNF1A | A0A0A0MQU7, HNF1A, Hepatocyte nuclear factor 1-alpha | 159-168 | - | 0.009046 | 9/0/0/ | 88/7/30/ | HNF-1\_N | Family | 3 | OMIM, PDB, | 0.3609 | Hepatocellular carcinoma (HCC) |
| HNF1A | A0A0A0MQU7, HNF1A, Hepatocyte nuclear factor 1-alpha | 203-279 | - | 5.965e-21 | 66/3/16/ | 88/7/30/ | Homeobox | Domain | 16 | PDB, Uniprot\_motif, OMIM, Region\_of\_interest, PhosphoELM\_modification, Uniprot\_modification, | 0.0309 | Hepatocellular carcinoma (HCC) |
| HNRNPCL1 | O60812, HNRNPCL1, Heterogeneous nuclear ribonucleoprotein C-like 1 | 253-262 | - | 0.001935 | 7/0/0/ | 11/0/0/ |  |  | 0 | - | 0.4841 | Pancreatic cancer |
| HRAS | P01112, HRAS, GTPase HRas | 12-13 | HRAS\_ENST00000397594|12-18 | 0 | 782/1/0/ | 1253/2/0/ | MnmE\_helical | Family | 7 | OMIM, PDB, NPB\_site, | 0.5406 | Skin neoplasm, Bladder cancer, Squamous cell carcinoma |
| HRAS | P01112, HRAS, GTPase HRas | 61-61 | HRAS\_ENST00000397594|61-61 | 6.321e-140 | 439/0/0/ | 1253/2/0/ | MnmE\_helical | Family | 5 | NPB\_site, PDB, | 0.5813 | Thyroid cancer |
| ID3 | Q02535, ID3, DNA-binding protein inhibitor ID-3 | 44-76 | - | 6.137e-05 | 16/1/1/ | 18/1/1/ | HLH | Domain | 1 | PDB, | 0 | Lymphoma |
| IDH1 | O75874, IDH1, Isocitrate dehydrogenase [NADP] cytoplasmic | 132-132 | - | 0 | 5106/0/0/ | 5148/0/0/ | Iso\_dh | Domain | 2 | PDB, Binding\_site, | 0.3906 | Glioma, Brain cancer, Acute myeloid leukemia, Bone cancer, Glioblastoma |
| IDH2 | P48735, IDH2, Isocitrate dehydrogenase [NADP], mitochondrial | 140-140 | - | 1.113e-196 | 502/0/0/ | 820/0/0/ | Iso\_dh | Domain | 3 | Region\_of\_interest, OMIM, PDB, | 0.1218 | Acute myeloid leukemia |
| IDH2 | P48735, IDH2, Isocitrate dehydrogenase [NADP], mitochondrial | 171-172 | - | 1.287e-99 | 304/0/0/ | 820/0/0/ | Iso\_dh | Domain | 3 | Binding\_site, OMIM, PDB, | 0.4749 | Acute myeloid leukemia, Glioma |
| IGFBP2 | P18065, IGFBP2, Insulin-like growth factor-binding protein 2 | 176-195 | - | 0.002331 | 6/0/0/ | 7/0/0/ |  |  | 0 | - | 0.4962 | - |
| IKBKB | O14920, IKBKB, Inhibitor of nuclear factor kappa-B kinase subunit beta | 171-171 | - | 3.365e-05 | 10/0/0/ | 12/0/0/ | Pkinase | Domain | 1 | PDB, | 0 | Lymphoma |
| IKZF3 | Q9UKT9, IKZF3, Zinc finger protein Aiolos | 160-162 | - | 0.001638 | 8/0/0/ | 20/0/0/ | zf-C2H2 | Domain | 0 | - | 0 | Lymphoma |
| IL36G | Q9NZH8, IL36G, Interleukin-36 gamma | 114-122 | - | 0.003497 | 6/0/0/ | 8/0/0/ | IL1 | Domain | 1 | PDB, | 0.4962 | - |
| IL6ST | P40189, IL6ST, Interleukin-6 receptor subunit beta | 168-216 | - | 6.839e-100 | 7/3/347/ | 26/3/355/ | IL6Ra-bind | Domain | 1 | PDB, | 0.0053 | Hepatocellular carcinoma (HCC) |
| IL6ST | P40189, IL6ST, Interleukin-6 receptor subunit beta | 415-421 | - | 0.00125 | 1/0/8/ | 26/3/355/ |  |  | 1 | PDB, | 0.0966 | Hepatocellular carcinoma (HCC) |
| IL7R | P16871, IL7R, Interleukin-7 receptor subunit alpha | 237-254 | - | 1.712e-49 | 8/66/55/ | 29/66/55/ |  |  | 0 | - | 0.0251 | Lymphoma |
| IRF2 | P14316, IRF2, Interferon regulatory factor 2 | 171-183 | - | 0.00125 | 1/0/7/ | 7/0/7/ |  |  | 0 | - | 0.2698 | Lymphoma, Ovarian cancer |
| IRF6 | O14896, IRF6, Interferon regulatory factor 6 | 84-92 | - | 0.008429 | 6/0/0/ | 15/0/0/ | IRF | Domain | 4 | OMIM, | 0.3441 | Pancreatic cancer |
| ISX | Q2M1V0, ISX, Intestine-specific homeobox | 80-86 | - | 0.002609 | 7/0/0/ | 13/0/0/ | Homeobox | Domain | 1 | Scansite\_motif, | 0.2205 | Melanoma |
| JAK1 | P23458, JAK1, Tyrosine-protein kinase JAK1 | 604-660 | - | 5.36e-05 | 15/2/6/ | 81/2/6/ | Pkinase\_Tyr | Domain | 1 | PDB, | 0.2674 | Lymphoma |
| JAK1 | P23458, JAK1, Tyrosine-protein kinase JAK1 | 695-751 | - | 4.293e-05 | 23/0/0/ | 81/2/6/ | Pkinase\_Tyr | Domain | 1 | PDB, | 0.3008 | Lymphoma |
| JAK2 | O60674, JAK2, Tyrosine-protein kinase JAK2 | 533-556 | - | 4.328e-127 | 31/10/312/ | 35330/15/318/ | Pkinase\_Tyr | Domain | 1 | PDB, | 0.1032 | Myeloproliferative disease |
| JAK2 | O60674, JAK2, Tyrosine-protein kinase JAK2 | 617-617 | - | 0 | 35147/0/0/ | 35330/15/318/ | Pkinase\_Tyr | Domain | 2 | PDB, OMIM, | 0.0499 | Chronic myeloid leukemia, Myeloproliferative disease, Other haematopoietic/lymphoid disorder, Acute myeloid leukemia |
| JAK2 | O60674, JAK2, Tyrosine-protein kinase JAK2 | 681-686 | - | 0.001054 | 63/5/5/ | 35330/15/318/ | Pkinase\_Tyr | Domain | 1 | PDB, | 0 | Lymphoma |
| JAK3 | P52333, JAK3, Tyrosine-protein kinase JAK3 | 501-511 | JAK3\_ENST00000458235|507-511 | 1.314e-08 | 28/0/0/ | 136/1/0/ |  |  | 1 | Scansite\_motif, | 0.1954 | Lymphoma |
| JAK3 | P52333, JAK3, Tyrosine-protein kinase JAK3 | 572-573 | - | 9.71e-07 | 19/0/0/ | 136/1/0/ | Pkinase\_Tyr | Domain | 0 | - | 0.1596 | Lymphoma, Acute myeloid leukemia |
| JAK3 | P52333, JAK3, Tyrosine-protein kinase JAK3 | 656-664 | - | 2.775e-07 | 24/0/0/ | 136/1/0/ | Pkinase\_Tyr | Domain | 0 | - | 0.2458 | Other haematopoietic/lymphoid disorder, Lymphoma |
| KANK3 | Q6NY19-2, KANK3, Isoform 2 of KN motif and ankyrin repeat domain-containing protein 3 | 489-492 | - | 6.25e-05 | 1/0/12/ | 8/0/15/ |  |  | 0 | - | 0.3026 | Lymphoma, Breast cancer, Acute myeloid leukemia |
| KCNJ5 | P48544, KCNJ5, G protein-activated inward rectifier potassium channel 4 | 145-168 | - | 0 | 646/2/7/ | 663/2/7/ | IRK | Family | 3 | Uniprot\_motif, OMIM, | 0.0032 | Adrenal tumor |
| KCNN2 | Q9H2S1, KCNN2, Small conductance calcium-activated potassium channel protein 2 | 58-78 | - | 0.008333 | 0/2/4/ | 16/2/4/ |  |  | 0 | - | 0.2403 | Renal cell carcinoma |
| KCTD5 | Q9NXV2, KCTD5, BTB/POZ domain-containing protein KCTD5 | 200-202 | - | 0.003968 | 5/0/0/ | 5/0/0/ |  |  | 1 | PDB, | 0.1864 | Lymphoma, Pancreatic cancer |
| KIAA0355 | O15063, KIAA0355, Uncharacterized protein KIAA0355 | 164-168 | - | 0.003993 | 7/0/0/ | 19/0/5/ | DUF4745 | Family | 0 | - | 0 | Ovarian cancer |
| KIT | P10721, KIT, Mast/stem cell growth factor receptor Kit | 417-422 | - | 3.689e-42 | 5/11/112/ | 1771/207/2919/ | Ig\_3 | Domain | 1 | PDB, | 0.1128 | Acute myeloid leukemia |
| KIT | P10721, KIT, Mast/stem cell growth factor receptor Kit | 496-506 | - | 7.346e-61 | 8/149/31/ | 1771/207/2919/ | Ig\_3 | Domain | 1 | PDB, | 0.1965 | Gastrointestinal stromal tumor |
| KIT | P10721, KIT, Mast/stem cell growth factor receptor Kit | 552-592 | - | 0 | 433/43/2514/ | 1771/207/2919/ | Pkinase\_Tyr | Domain | 13 | Scansite\_motif, Uniprot\_modification, Region\_of\_interest, PhosphoELM\_modification, OMIM, | 0.1202 | Gastrointestinal stromal tumor, Melanoma |
| KIT | P10721, KIT, Mast/stem cell growth factor receptor Kit | 642-642 | - | 1.681e-10 | 40/0/0/ | 1771/207/2919/ | Pkinase\_Tyr | Domain | 0 | - | 0.2436 | Melanoma, Gastrointestinal stromal tumor |
| KIT | P10721, KIT, Mast/stem cell growth factor receptor Kit | 654-654 | - | 3.144e-05 | 21/0/0/ | 1771/207/2919/ | Pkinase\_Tyr | Domain | 0 | - | 0.053 | Gastrointestinal stromal tumor |
| KIT | P10721, KIT, Mast/stem cell growth factor receptor Kit | 715-716 | - | 0.007569 | 1/0/7/ | 1771/207/2919/ | Pkinase\_Tyr | Domain | 2 | Scansite\_motif, | 0 | Gastrointestinal stromal tumor |
| KIT | P10721, KIT, Mast/stem cell growth factor receptor Kit | 816-816 | - | 0 | 950/1/0/ | 1771/207/2919/ | Pkinase\_Tyr | Domain | 1 | PDB, | 0.2019 | Other haematopoietic/lymphoid disorder, Acute myeloid leukemia |
| KIT | P10721, KIT, Mast/stem cell growth factor receptor Kit | 820-825 | - | 1.2e-25 | 121/0/0/ | 1771/207/2919/ | Pkinase\_Tyr | Domain | 5 | Uniprot\_modification, PhosphoELM\_modification, PDB, | 0.4715 | Gastrointestinal stromal tumor |
| KLF4 | O43474-1, KLF4, Isoform 2 of Krueppel-like factor 4 | 409-413 | - | 3.259e-31 | 65/0/0/ | 73/0/0/ |  |  | 1 | PDB, | 0.0976 | Meningioma |
| KLHL24 | Q6TFL4, KLHL24, Kelch-like protein 24 | 521-527 | - | 0.009828 | 6/0/0/ | 19/0/0/ |  |  | 0 | - | 0.2403 | Pancreatic cancer |
| KLHL6 | Q8WZ60, KLHL6, Kelch-like protein 6 | 49-94 | - | 0.0004549 | 14/0/0/ | 30/0/0/ | BTB | Domain | 0 | - | 0.1136 | Lymphoma |
| KMT2A | Q03164, KMT2A, Histone-lysine N-methyltransferase 2A | 1469-1480 | - | 0.003074 | 3/0/7/ | 93/0/8/ | PHD | Domain | 0 | - | 0.2487 | Breast cancer, Bladder cancer |
| KMT2C | Q8NEZ4, KMT2C, Histone-lysine N-methyltransferase 2C | 288-368 | MLL3\_ENST00000355193|288-368 | 7.428e-06 | 25/0/0/ | 204/0/1/ | zf-HC5HC2H | Domain | 1 | PDB, | 0.5071 | Breast cancer |
| KMT2D | O14686-3, KMT2D, Isoform 3 of Histone-lysine N-methyltransferase 2D | 5119-5164 | - | 0.008885 | 10/0/1/ | 184/1/4/ | SET | Family | 2 | OMIM, PDB, | 0.4615 | Lymphoma |
| KNG1\_ENST00000265023 | P01042, KNG1, Kininogen-1 | 440-450 | - | 0.007453 | 6/0/0/ | 13/0/0/ |  |  | 0 | - | 0.1763 | Hepatocellular carcinoma (HCC), Pancreatic cancer |
| KRAS | P01116-2, KRAS, Isoform 2B of GTPase KRas | 12-12 | KRAS\_ENST00000256078|12-12 | 0 | 26738/6/0/ | 31929/24/9/ | MnmE\_helical | Family | 2 | PDB, OMIM, | 0.4088 | Stomach cancer, Endometrial cancer, Ovarian cancer, Other/various, Squamous cell carcinoma, Colorectal cancer, Non-small cell lung cancer, Lymphoma, Bile duct/gallbladder cancer, Thyroid cancer, Pancreatic cancer |
| KRAS | P01116-2, KRAS, Isoform 2B of GTPase KRas | 58-68 | - | 0.002172 | 469/4/6/ | 31929/24/9/ | MnmE\_helical | Family | 4 | PDB, OMIM, | 0.5873 | Colorectal cancer |
| KRT15 | P19012, KRT15, Keratin, type I cytoskeletal 15 | 202-205 | - | 0.0005164 | 9/0/0/ | 18/0/0/ | Filament | Family | 2 | Region\_of\_interest, | 0.5071 | Prostate cancer |
| KRT8 | P05787, KRT8, Keratin, type II cytoskeletal 8 | 31-31 | - | 0.0002288 | 9/0/0/ | 13/0/0/ | Keratin\_2\_head | Family | 1 | Region\_of\_interest, | 0.3952 | Prostate cancer |
| KRTAP4-11 | Q9BYQ6, KRTAP4-11, Keratin-associated protein 4-11 | 161-161 | - | 0.005271 | 7/0/0/ | 30/0/0/ |  |  | 4 | Region\_of\_interest, | 0.3537 | Prostate cancer, Glioma |
| KRTAP4-11 | Q9BYQ6, KRTAP4-11, Keratin-associated protein 4-11 | 86-93 | - | 0.006091 | 9/0/0/ | 30/0/0/ |  |  | 4 | Region\_of\_interest, | 0.4218 | Renal cell carcinoma |
| KRTAP4-5 | Q9BYR2, KRTAP4-5, Keratin-associated protein 4-5 | 69-79 | - | 0.001923 | 4/0/6/ | 8/0/6/ | Keratin\_B2\_2 | Family | 1 | Region\_of\_interest, | 0.2604 | Breast cancer |
| KRTAP4-7 | -, -, - | 57-57 | - | 8.06e-07 | 14/0/0/ | 18/0/0/ | Keratin\_B2\_2 | Family | 0 | - | 0.4633 | Breast cancer |
| KRTAP4-8 | Q9BYQ9, KRTAP4-8, Keratin-associated protein 4-8 | 95-95 | - | 5.261e-06 | 12/0/0/ | 15/0/0/ | Keratin\_B2\_2 | Family | 1 | Region\_of\_interest, | 0.4962 | Glioma |
| KRTAP4-9\_ENST00000377734 | -, -, - | 57-57 | - | 3.407e-06 | 14/0/0/ | 23/0/0/ | Keratin\_B2\_2 | Family | 0 | - | 0.4633 | Breast cancer |
| KRTAP4-9 | Q9BYQ8, KRTAP4-9, Keratin-associated protein 4-9 | 16-18 | - | 0.0004635 | 9/0/0/ | 17/0/0/ | Keratin\_B2 | Family | 0 | - | 0.4644 | Prostate cancer |
| KRTAP9-9 | Q9BYP9-3, KRTAP9-9, Isoform 3 of Keratin-associated protein 9-9 | 18-30 | - | 9.921e-06 | 0/5/8/ | 3/5/8/ | Keratin\_B2\_2 | Family | 0 | - | 0.2378 | Pancreatic cancer, Breast cancer |
| LILRA1 | O75019, LILRA1, Leukocyte immunoglobulin-like receptor subfamily A member 1 | 72-77 | - | 0.003993 | 7/0/0/ | 19/0/0/ | Ig\_2 | Domain | 0 | - | 0.3744 | Colorectal cancer, Bile duct/gallbladder cancer, Pancreatic cancer |
| LILRA2 | Q8N149-2, LILRA2, Isoform 2 of Leukocyte immunoglobulin-like receptor subfamily A member 2 | 179-204 | - | 0.002307 | 10/0/0/ | 25/0/0/ | Ig\_3 | Domain | 4 | PDB, Scansite\_motif, | 0.508 | Lymphoma |
| LILRB5 | O75023, LILRB5, Leukocyte immunoglobulin-like receptor subfamily B member 5 | 495-514 | - | 0.002899 | 7/0/0/ | 14/0/0/ |  |  | 3 | Scansite\_motif, Uniprot\_modification, | 0.3537 | Hepatocellular carcinoma (HCC), Prostate cancer |
| LOC442444 | Q5HY64, FAM47C, Putative protein FAM47C | 387-415 | - | 0.008429 | 6/0/0/ | 15/0/0/ | FAM47 | Family | 1 | Scansite\_motif, | 0.3682 | Stomach cancer, Hepatocellular carcinoma (HCC) |
| LOC51123 | Q9Y5V0, ZNF706, Zinc finger protein 706 | 3-8 | ZNF706|3-8 | 0.007576 | 5/0/0/ | 6/0/0/ | 4F5 | Family | 0 | - | 0.3689 | Glioma |
| LRP2 | P98164, LRP2, Low-density lipoprotein receptor-related protein 2 | 3043-3050 | - | 0.007118 | 7/0/0/ | 116/0/0/ | Ldl\_recept\_a | Repeat | 0 | - | 0.3537 | Glioblastoma, Colorectal cancer |
| MAP2K1 | Q02750, MAP2K1, Dual specificity mitogen-activated protein kinase kinase 1 | 42-67 | - | 2.695e-08 | 20/0/27/ | 60/0/49/ | Pkinase | Domain | 3 | ELM\_instance, OMIM, PDB, | 0.3216 | Lymphoma |
| MAP2K1 | Q02750, MAP2K1, Dual specificity mitogen-activated protein kinase kinase 1 | 93-130 | - | 2.358e-12 | 28/0/22/ | 60/0/49/ | Pkinase | Domain | 6 | PDB, Scansite\_motif, OMIM, Binding\_site, | 0.3511 | Lymphoma, Melanoma |
| MAP2K4 | P45985, MAP2K4, Dual specificity mitogen-activated protein kinase kinase 4 | 228-309 | - | 0.0008144 | 29/0/0/ | 57/1/0/ | Pkinase | Domain | 9 | PhosphoELM\_modification, Active\_site, PDB, Uniprot\_modification, | 0.5027 | Breast cancer |
| MAP3K10 | Q02779, MAP3K10, Mitogen-activated protein kinase kinase kinase 10 | 233-252 | - | 0.009202 | 6/0/0/ | 17/0/0/ | Pkinase\_Tyr | Domain | 0 | - | 0.4962 | - |
| MAPK1 | P28482, MAPK1, Mitogen-activated protein kinase 1 | 316-322 | - | 0.0001241 | 10/0/0/ | 16/0/0/ |  |  | 2 | Uniprot\_motif, PDB, | 0.3016 | Squamous cell carcinoma |
| MAX | P61244, MAX, Protein max | 60-63 | MAX\_ENST00000284165|60-63 | 0.003161 | 7/0/0/ | 15/0/6/ | HLH | Domain | 1 | PDB, | 0.4292 | Acute myeloid leukemia, Glioma |
| MED12\_ENST00000374080 | Q93074, MED12, Mediator of RNA polymerase II transcription subunit 12 | 1220-1229 | - | 0.0003291 | 11/0/0/ | 75/0/1/ |  |  | 0 | - | 0.1662 | Prostate cancer |
| MED12 | Q93074, MED12, Mediator of RNA polymerase II transcription subunit 12 | 25-52 | MED12\_ENST00000374080|36-45 | 0 | 579/36/856/ | 644/36/892/ |  |  | 0 | - | 0.1352 | Other soft tissue tumor |
| MED28 | Q9H204, MED28, Mediator of RNA polymerase II transcription subunit 28 | 3-8 | - | 0.003968 | 5/0/0/ | 5/0/0/ |  |  | 0 | - | 0.2921 | Hepatocellular carcinoma (HCC), Squamous cell carcinoma |
| MEF2A\_ENST00000354410 | Q02078-5, MEF2A, Isoform 5 of Myocyte-specific enhancer factor 2A | 99-110 | MEF2A|99-110 | 2.499e-05 | 11/0/0/ | 15/0/0/ | HJURP\_C | Domain | 1 | Scansite\_motif, | 0.3236 | Other soft tissue tumor |
| MEF2B | Q02080-2, MEF2B, Isoform 2 of Myocyte-specific enhancer factor 2B | 78-83 | MEF2BNB-MEF2B|77-83 | 0.0001594 | 11/0/0/ | 30/0/0/ |  |  | 1 | PDB, | 0 | Lymphoma |
| MEN1 | O00255-2, MEN1, Isoform 2 of Menin | 158-169 | - | 0.0003804 | 11/0/2/ | 75/1/169/ | Menin | Family | 8 | PDB, OMIM, | 0.4194 | Thyroid cancer, Pancreatic cancer |
| MEN1 | O00255-2, MEN1, Isoform 2 of Menin | 262-275 | - | 8.626e-05 | 2/0/18/ | 75/1/169/ | Menin | Family | 6 | OMIM, Region\_of\_interest, PDB, | 0.09 | Thyroid cancer |
| MEN1 | O00255-2, MEN1, Isoform 2 of Menin | 3-47 | - | 0.002262 | 10/0/69/ | 75/1/169/ | Menin | Family | 7 | PDB, OMIM, | 0.1006 | Thyroid cancer |
| METTL14 | Q9HCE5, METTL14, N6-adenosine-methyltransferase subunit METTL14 | 298-299 | - | 0.0008238 | 8/0/0/ | 13/0/0/ | MT-A70 | Family | 0 | - | 0.3359 | Endometrial cancer, Pancreatic cancer |
| MET | P08581-2, MET, Isoform 2 of Hepatocyte growth factor receptor | 1010-1010 | - | 1.081e-05 | 16/0/4/ | 182/0/188/ |  |  | 1 | PhosphoELM\_modification, | 0.3884 | Non-small cell lung cancer |
| MET | P08581-2, MET, Isoform 2 of Hepatocyte growth factor receptor | 1110-1120 | - | 0.005282 | 10/0/0/ | 182/0/188/ | Pkinase\_Tyr | Domain | 2 | NPB\_site, PDB, | 0.3399 | Renal cell carcinoma |
| MET | P08581-2, MET, Isoform 2 of Hepatocyte growth factor receptor | 1238-1271 | - | 9.891e-22 | 74/0/0/ | 182/0/188/ | Pkinase\_Tyr | Domain | 16 | PhosphoELM\_modification, PDB, OMIM, Uniprot\_modification, Region\_of\_interest, | 0.2286 | Squamous cell carcinoma |
| MFAP3L | O75121, MFAP3L, Microfibrillar-associated protein 3-like | 27-34 | - | 0.0025 | 0/0/16/ | 11/0/16/ |  |  | 0 | - | 0 | Ovarian cancer |
| MGAT4C | Q9UBM8, MGAT4C, Alpha-1,3-mannosyl-glycoprotein 4-beta-N-acetylglucosaminyltransferase C | 149-149 | - | 0.004158 | 7/0/0/ | 20/0/0/ | Glyco\_transf\_54 | Family | 0 | - | 0.4292 | Glioma, Hepatocellular carcinoma (HCC) |
| MGC33414 | Q8IZ20, ZNF683, Zinc finger protein 683 | 228-235 | ZNF683|243-250 | 6.25e-05 | 1/0/24/ | 13/0/24/ |  |  | 0 | - | 0.2157 | Ovarian cancer, Acute myeloid leukemia |
| MLH1 | P40692, MLH1, DNA mismatch repair protein Mlh1 | 381-385 | - | 1.787e-05 | 14/0/0/ | 44/0/1/ |  |  | 0 | - | 0.3366 | Ovarian cancer |
| MPL | P40238, MPL, Thrombopoietin receptor | 496-520 | MPL\_ENST00000413998|505-515 | 5.223e-164 | 358/3/8/ | 390/3/8/ |  |  | 2 | OMIM, | 0.0757 | Myeloproliferative disease |
| MRGPRX3 | Q96LB0, MRGPRX3, Mas-related G-protein coupled receptor member X3 | 34-41 | - | 0.008837 | 6/0/0/ | 16/0/0/ |  |  | 0 | - | 0.3682 | Glioblastoma, Other soft tissue tumor |
| MST1 | G3XAK1, MST1, Hepatocyte growth factor-like protein | 347-374 | - | 0.002414 | 10/0/0/ | 26/0/0/ | Kringle | Domain | 0 | - | 0.338 | Glioma |
| MTOR | P42345, MTOR, Serine/threonine-protein kinase mTOR | 1971-1977 | - | 0.0071 | 7/0/0/ | 113/0/1/ |  |  | 1 | PDB, | 0.2781 | Lymphoma, Renal cell carcinoma |
| MTOR | P42345, MTOR, Serine/threonine-protein kinase mTOR | 2215-2223 | - | 0.0071 | 7/0/0/ | 113/0/1/ | PI3\_PI4\_kinase | Family | 1 | PDB, | 0.3537 | Colorectal cancer, Renal cell carcinoma |
| MTOR | P42345, MTOR, Serine/threonine-protein kinase mTOR | 2406-2427 | - | 0.0006688 | 13/0/0/ | 113/0/1/ | PI3\_PI4\_kinase | Family | 1 | PDB, | 0.4222 | Renal cell carcinoma |
| MTOR | P42345, MTOR, Serine/threonine-protein kinase mTOR | 2500-2512 | - | 0.004943 | 10/0/0/ | 113/0/1/ |  |  | 2 | PDB, Scansite\_motif, | 0.4148 | Renal cell carcinoma |
| MUC6 | Q6W4X9, MUC6, Mucin-6 | 1873-1995 | MUC6\_ENST00000421673|1911-1995 | 3.663e-07 | 26/0/4/ | 87/0/16/ |  |  | 2 | Region\_of\_interest, Scansite\_motif, | 0.4114 | Prostate cancer |
| MYCN | P04198, MYCN, N-myc proto-oncogene protein | 44-44 | - | 0.001724 | 8/0/0/ | 21/0/0/ | Myc\_N | Family | 0 | - | 0.3359 | Pancreatic cancer, Neuroblastoma |
| MYC\_ENST00000377970 | P01106-2, MYC, Isoform 2 of Myc proto-oncogene protein | 153-159 | MYC|138-144 | 0.0091 | 9/0/0/ | 91/0/38/ | Myc\_N | Family | 0 | - | 0 | Lymphoma |
| MYC\_ENST00000377970 | P01106-2, MYC, Isoform 2 of Myc proto-oncogene protein | 3-97 | MYC|2-82 | 1.296e-05 | 47/0/34/ | 91/0/38/ | Myc\_N | Family | 14 | Scansite\_motif, ELM\_instance, | 0.0622 | Lymphoma |
| MYD88 | A0A0A0MS70, MYD88, Myeloid differentiation primary response protein MyD88 | 265-265 | - | 0 | 1554/0/0/ | 1608/0/9/ | TIR | Family | 1 | PDB, | 0 | Lymphoma |
| MYOD1 | P15172, MYOD1, Myoblast determination protein 1 | 122-122 | - | 5.877e-13 | 25/0/0/ | 28/0/0/ | HLH | Domain | 4 | Scansite\_motif, PDB, | 0 | Other soft tissue tumor |
| NAB2 | Q15742, NAB2, NGFI-A-binding protein 2 | 208-217 | - | 0.006865 | 6/0/0/ | 12/0/1/ |  |  | 0 | - | 0.3441 | Glioma |
| NALCN | Q8IZF0, NALCN, Sodium leak channel non-selective protein | 90-100 | NALCN\_ENST00000376196|90-100 | 0.0006952 | 10/0/0/ | 71/0/0/ | Ion\_trans | Family | 0 | - | 0.09 | Pancreatic cancer |
| NCKAP5L\_ENST00000335999 | Q9HCH0, NCKAP5L, Nck-associated protein 5-like | 1171-1201 | - | 0.008837 | 6/0/0/ | 16/0/0/ | NCKAP5 | Family | 0 | - | 0.1787 | Hepatocellular carcinoma (HCC), Unknown |
| NF1 | P21359, NF1, Neurofibromin | 1444-1447 | - | 0.003616 | 8/0/0/ | 185/0/117/ | RasGAP | Family | 3 | PDB, OMIM, | 0.5279 | Lymphoma |
| NF1 | P21359, NF1, Neurofibromin | 176-197 | - | 0.00929 | 4/0/9/ | 185/0/117/ |  |  | 3 | Scansite\_motif, OMIM, | 0.2198 | Other soft tissue tumor |
| NF1 | P21359, NF1, Neurofibromin | 765-809 | - | 3.14e-05 | 6/0/41/ | 185/0/117/ |  |  | 4 | OMIM, | 0.2771 | Other soft tissue tumor, Adrenal tumor |
| NF1 | P21359, NF1, Neurofibromin | 845-853 | - | 0.00929 | 4/0/9/ | 185/0/117/ |  |  | 2 | OMIM, | 0.2595 | Other soft tissue tumor |
| NF2 | P35240, NF2, Merlin | 13-156 | - | 8.085e-06 | 16/0/374/ | 52/0/553/ | FERM\_M | Domain | 7 | Scansite\_motif, OMIM, PDB, | 0.1341 | Other soft tissue tumor |
| NF2 | P35240, NF2, Merlin | 205-219 | - | 0.001497 | 7/0/3/ | 52/0/553/ | FERM\_M | Domain | 1 | PDB, | 0.2487 | Meningioma, Other soft tissue tumor |
| NFE2L2 | Q16236, NFE2L2, Nuclear factor erythroid 2-related factor 2 | 23-36 | - | 2.434e-21 | 59/1/7/ | 126/2/13/ |  |  | 2 | ELM\_instance, PDB, | 0.4625 | Squamous cell carcinoma |
| NFE2L2 | Q16236, NFE2L2, Nuclear factor erythroid 2-related factor 2 | 75-83 | - | 2.157e-22 | 59/1/6/ | 126/2/13/ |  |  | 2 | PDB, ELM\_instance, | 0.3703 | Squamous cell carcinoma |
| NFXL1 | Q6ZNB6, NFXL1, NF-X1-type zinc finger protein NFXL1 | 815-815 | - | 0.00431 | 7/0/0/ | 21/0/0/ |  |  | 0 | - | 0 | Ovarian cancer |
| NOTCH1\_ENST00000277541 | P46531, NOTCH1, Neurogenic locus notch homolog protein 1 | 465-484 | - | 0.0009757 | 15/0/0/ | 222/1/15/ | EGF | Domain | 1 | PDB, | 0.4478 | Squamous cell carcinoma, Glioma |
| NOTCH1 | P46531, NOTCH1, Neurogenic locus notch homolog protein 1 | 1547-1770 | NOTCH1\_ENST00000277541|1574-1609  NOTCH1\_ENST00000277541|1676-1680  NOTCH1\_ENST00000277541|1718-1721 | 2.262e-136 | 346/92/208/ | 542/100/352/ | NODP | Family | 1 | PDB, | 0.0471 | Lymphoma |
| NOTCH1 | P46531, NOTCH1, Neurogenic locus notch homolog protein 1 | 342-413 | NOTCH1\_ENST00000277541|332-408 | 0.0003448 | 26/0/11/ | 542/100/352/ | EGF\_CA | Domain | 1 | PDB, | 0.3418 | Squamous cell carcinoma |
| NOTCH2 | Q04721, NOTCH2, Neurogenic locus notch homolog protein 2 | 21-21 | - | 0.003308 | 8/0/0/ | 88/0/0/ |  |  | 0 | - | 0.3359 | Breast cancer, Bladder cancer |
| NPM1 | P06748, NPM1, Nucleophosmin | 280-293 | - | 3.746e-05 | 5/3/3/ | 7/4/3/ | NPM1-C | Domain | 3 | Region\_of\_interest, Uniprot\_modification, PDB, | 0 | Acute myeloid leukemia |
| NRAS | P01111, NRAS, GTPase NRas | 12-13 | - | 0 | 1433/0/0/ | 3710/1/0/ | MnmE\_helical | Family | 4 | OMIM, PDB, NPB\_site, | 0.5505 | Lymphoma, Melanoma, Colorectal cancer, Acute myeloid leukemia |
| NRAS | P01111, NRAS, GTPase NRas | 61-61 | - | 0 | 2225/0/0/ | 3710/1/0/ | MnmE\_helical | Family | 3 | NPB\_site, PDB, OMIM, | 0.5131 | Lymphoma, Skin neoplasm, Melanoma, Thyroid cancer, Acute myeloid leukemia, Colorectal cancer |
| NT5C2 | P49902, NT5C2, Cytosolic purine 5'-nucleotidase | 238-238 | - | 4.991e-05 | 13/0/0/ | 50/0/0/ | 5\_nucleotid | Family | 1 | PDB, | 0 | Lymphoma |
| NT5C2 | P49902, NT5C2, Cytosolic purine 5'-nucleotidase | 359-367 | NT5C2\_ENST00000423468|331-338 | 4.712e-07 | 21/0/0/ | 50/0/0/ | 5\_nucleotid | Family | 1 | PDB, | 0 | Lymphoma |
| NTM | Q9P121, NTM, Neurotrimin | 162-184 | NTM\_ENST00000374791|166-172  NTM\_ENST00000427481|157-175  NTM\_ENST00000539799|166-184 | 0.003678 | 11/0/0/ | 23/0/0/ | I-set | Domain | 3 | Scansite\_motif, | 0.3934 | Pancreatic cancer |
| NTRK3 | Q16288-3, NTRK3, Isoform 3 of NT-3 growth factor receptor | 306-307 | - | 0.006706 | 7/0/0/ | 72/0/0/ |  |  | 1 | PDB, | 0.2647 | Pancreatic cancer, Non-small cell lung cancer |
| NTRK3 | Q16288-3, NTRK3, Isoform 3 of NT-3 growth factor receptor | 731-732 | - | 0.003181 | 8/0/0/ | 72/0/0/ | Pkinase\_Tyr | Domain | 1 | PDB, | 0.4618 | Colorectal cancer |
| OBSCN | -, -, - | 3738-3741 | OBSCN\_ENST00000284548|3684-3687  OBSCN\_ENST00000359599|3967-3970  OBSCN\_ENST00000570156|3868-3871 | 0.007239 | 7/0/0/ | 141/0/0/ | I-set | Domain | 1 | PDB, | 0.1657 | Hepatocellular carcinoma (HCC), Ovarian cancer |
| OLIG3 | Q7RTU3, OLIG3, Oligodendrocyte transcription factor 3 | 93-118 | - | 0.003968 | 5/0/0/ | 5/0/0/ | HLH | Domain | 0 | - | 0.2921 | Colorectal cancer, Pancreatic cancer |
| OR10H4 | Q8NGA5, OR10H4, Olfactory receptor 10H4 | 167-168 | - | 0.006192 | 6/0/0/ | 11/0/0/ | 7tm\_4 | Family | 0 | - | 0.1248 | Pancreatic cancer |
| OR4C3 | Q8NH37, OR4C3, Olfactory receptor 4C3 | 52-59 | - | 0.00125 | 1/0/6/ | 7/0/6/ | 7tm\_4 | Family | 0 | - | 0.1136 | Pancreatic cancer |
| OR4F17 | Q8NGA8, OR4F17, Olfactory receptor 4F17 | 104-113 | - | 0.002331 | 6/0/0/ | 7/0/0/ | 7tm\_4 | Family | 0 | - | 0.1248 | Breast cancer |
| OR52M1 | Q8NGK5, OR52M1, Olfactory receptor 52M1 | 145-180 | - | 0.00193 | 11/0/0/ | 17/0/0/ | 7tm\_4 | Family | 0 | - | 0.3211 | Pancreatic cancer |
| OR5P2 | Q8WZ92, OR5P2, Olfactory receptor 5P2 | 35-44 | - | 0.003361 | 2/4/0/ | 9/4/0/ | 7tm\_4 | Family | 0 | - | 0.4322 | Breast cancer |
| OR5W2 | Q8NH69, OR5W2, Olfactory receptor 5W2 | 258-267 | - | 0.001099 | 8/0/0/ | 15/0/0/ | 7tm\_4 | Family | 0 | - | 0.4618 | Pancreatic cancer |
| OTOP1 | Q7RTM1, OTOP1, Otopetrin-1 | 104-106 | - | 0.000125 | 0/0/9/ | 25/0/9/ |  |  | 0 | - | 0.3042 | Lymphoma, Bladder cancer, Melanoma |
| OTUD4 | Q01804-3, OTUD4, Isoform 3 of OTU domain-containing protein 4 | 909-909 | OTUD4\_ENST00000447906|974-974 | 0.0003358 | 10/0/0/ | 26/0/1/ |  |  | 0 | - | 0.2851 | Prostate cancer, Thyroid cancer |
| PARP4 | Q9UKK3, PARP4, Poly [ADP-ribose] polymerase 4 | 1039-1063 | - | 0.005549 | 7/0/0/ | 34/0/1/ |  |  | 0 | - | 0.3537 | Prostate cancer, Breast cancer |
| PAX5 | Q02548, PAX5, Paired box protein Pax-5 | 12-80 | - | 8.489e-11 | 44/0/0/ | 57/1/5/ | PAX | Domain | 1 | PDB, | 0.03 | Lymphoma |
| PBRM1 | Q86U86-5, PBRM1, Isoform 5 of Protein polybromo-1 | 874-880 | PBRM1\_ENST00000296302|874-880  PBRM1\_ENST00000356770|842-848 | 0.009969 | 4/0/2/ | 70/0/22/ |  |  | 1 | PDB, | 0.1763 | Renal cell carcinoma, Pancreatic cancer |
| PCBP2 | Q15366-2, PCBP2, Isoform 2 of Poly(rC)-binding protein 2 | 301-315 | - | 0.002288 | 7/0/0/ | 12/0/0/ | KH\_1 | Domain | 1 | PDB, | 0.3744 | Bladder cancer, Hepatocellular carcinoma (HCC), Glioma |
| PCDH18 | Q9HCL0, PCDH18, Protocadherin-18 | 582-585 | - | 0.002417 | 8/0/0/ | 33/0/0/ |  |  | 0 | - | 0.4618 | Pancreatic cancer |
| PCF11 | O94913, PCF11, Pre-mRNA cleavage complex 2 protein Pcf11 | 683-701 | PCF11\_ENST00000298281|584-602 | 0.000451 | 10/0/0/ | 34/0/0/ |  |  | 0 | - | 0.3399 | Renal cell carcinoma |
| PCMTD1 | Q96MG8, PCMTD1, Protein-L-isoaspartate O-methyltransferase domain-containing protein 1 | 247-292 | PCMTD1\_ENST00000544451|196-205 | 0.0003604 | 17/0/0/ | 28/0/0/ |  |  | 0 | - | 0.4724 | Pancreatic cancer |
| PDE11A | Q9HCR9, PDE11A, Dual 3',5'-cyclic-AMP and -GMP phosphodiesterase 11A | 912-922 | PDE11A\_ENST00000358450|669-672 | 0.001984 | 1/5/0/ | 21/5/0/ |  |  | 0 | - | 0.3682 | Squamous cell carcinoma, Glioma |
| PDE7A\_ENST00000401827 | Q13946, PDE7A, High affinity cAMP-specific 3',5'-cyclic phosphodiesterase 7A | 390-390 | PDE7A|364-364 | 0.006865 | 6/0/0/ | 12/0/0/ | PDEase\_I | Domain | 2 | PDB, Region\_of\_interest, | 0 | Ovarian cancer |
| PDGFRA | P16234, PDGFRA, Platelet-derived growth factor receptor alpha | 554-571 | - | 5.289e-71 | 34/2/290/ | 354/6/481/ |  |  | 0 | - | 0.2609 | Other/various |
| PDGFRA | P16234, PDGFRA, Platelet-derived growth factor receptor alpha | 659-665 | - | 1.356e-05 | 22/0/0/ | 354/6/481/ | Pkinase\_Tyr | Domain | 0 | - | 0.3037 | Gastrointestinal stromal tumor |
| PDGFRA | P16234, PDGFRA, Platelet-derived growth factor receptor alpha | 841-849 | - | 5.894e-134 | 204/0/187/ | 354/6/481/ | Pkinase\_Tyr | Domain | 3 | PhosphoELM\_modification, Uniprot\_modification, OMIM, | 0.122 | Gastrointestinal stromal tumor |
| PEG3 | Q9GZU2, PEG3, Paternally-expressed gene 3 protein | 301-303 | ZIM2\_ENST00000326441|301-303 | 0.006659 | 7/0/0/ | 69/0/0/ |  |  | 0 | - | 0.4085 | Pancreatic cancer |
| PER3 | P56645, PER3, Period circadian protein homolog 3 | 989-998 | - | 0.009531 | 6/0/0/ | 18/0/0/ |  |  | 2 | Region\_of\_interest, Scansite\_motif, | 0.3682 | Hepatocellular carcinoma (HCC), Medulloblastoma |
| PHF6 | Q8IWS0, PHF6, PHD finger protein 6 | 269-340 | - | 0.0001956 | 28/0/0/ | 50/0/0/ | zf-HC5HC2H | Domain | 2 | PDB, Region\_of\_interest, | 0.298 | Lymphoma, Acute myeloid leukemia |
| PIK3CA | P42336, PIK3CA, Phosphatidylinositol 4,5-bisphosphate 3-kinase catalytic subunit alpha isoform | 102-118 | PIK3CA\_ENST00000263967|102-118 | 1.767e-16 | 93/1/43/ | 5069/2/113/ | PI3K\_p85B | Family | 3 | PDB, OMIM, | 0.6142 | - |
| PIK3CA | P42336, PIK3CA, Phosphatidylinositol 4,5-bisphosphate 3-kinase catalytic subunit alpha isoform | 1043-1044 | PIK3CA\_ENST00000263967|1040-1049 | 2.728e-24 | 112/0/0/ | 5069/2/113/ |  |  | 2 | PDB, OMIM, | 0.553 | Colorectal cancer |
| PIK3CA | P42336, PIK3CA, Phosphatidylinositol 4,5-bisphosphate 3-kinase catalytic subunit alpha isoform | 1047-1047 | - | 0 | 1881/0/0/ | 5069/2/113/ |  |  | 2 | OMIM, PDB, | 0.4339 | Colorectal cancer, Breast cancer, Endometrial cancer |
| PIK3CA | P42336, PIK3CA, Phosphatidylinositol 4,5-bisphosphate 3-kinase catalytic subunit alpha isoform | 345-345 | PIK3CA\_ENST00000263967|344-350 | 7.246e-13 | 58/0/0/ | 5069/2/113/ |  |  | 1 | PDB, | 0.2577 | Breast cancer |
| PIK3CA | P42336, PIK3CA, Phosphatidylinositol 4,5-bisphosphate 3-kinase catalytic subunit alpha isoform | 420-420 | PIK3CA\_ENST00000263967|418-422 | 3.189e-09 | 43/0/1/ | 5069/2/113/ | PI3K\_C2 | Domain | 2 | OMIM, PDB, | 0.4047 | Colorectal cancer, Breast cancer |
| PIK3CA | P42336, PIK3CA, Phosphatidylinositol 4,5-bisphosphate 3-kinase catalytic subunit alpha isoform | 447-456 | PIK3CA\_ENST00000263967|449-455 | 1.562e-08 | 16/0/41/ | 5069/2/113/ | PI3K\_C2 | Domain | 2 | PDB, OMIM, | 0.3992 | Breast cancer |
| PIK3CA | P42336, PIK3CA, Phosphatidylinositol 4,5-bisphosphate 3-kinase catalytic subunit alpha isoform | 538-545 | PIK3CA\_ENST00000263967|542-546 | 0 | 2073/0/0/ | 5069/2/113/ | PI3Ka | Family | 3 | PDB, OMIM, | 0.5659 | Bladder cancer, Endometrial cancer, Breast cancer, Colorectal cancer, Squamous cell carcinoma |
| PIK3CA | P42336, PIK3CA, Phosphatidylinositol 4,5-bisphosphate 3-kinase catalytic subunit alpha isoform | 9-22 | - | 0.000125 | 8/0/26/ | 5069/2/113/ |  |  | 1 | PDB, | 0.4083 | Endometrial cancer, Glioblastoma |
| PIK3R1 | P27986, PIK3R1, Phosphatidylinositol 3-kinase regulatory subunit alpha | 376-380 | - | 0.003469 | 6/0/2/ | 66/17/362/ | SH2 | Domain | 1 | PDB, | 0.1832 | Glioblastoma, Glioma |
| PIK3R1 | P27986, PIK3R1, Phosphatidylinositol 3-kinase regulatory subunit alpha | 446-475 | PIK3R1\_ENST00000320694|146-165  PIK3R1\_ENST00000336483|176-200 | 2.369e-22 | 9/11/137/ | 66/17/362/ | PI3K\_P85\_iSH2 | Domain | 4 | PhosphoELM\_modification, PDB, Uniprot\_modification, | 0.2297 | Endometrial cancer |
| PIK3R1 | P27986, PIK3R1, Phosphatidylinositol 3-kinase regulatory subunit alpha | 559-582 | PIK3R1\_ENST00000320694|259-281  PIK3R1\_ENST00000336483|290-311 | 7.999e-35 | 25/5/104/ | 66/17/362/ | PI3K\_P85\_iSH2 | Domain | 4 | PhosphoELM\_modification, Uniprot\_modification, PDB, | 0.4788 | Breast cancer |
| PIM1 | P11309-2, PIM1, Isoform 2 of Serine/threonine-protein kinase pim-1 | 23-28 | - | 0.002099 | 11/0/0/ | 61/0/0/ |  |  | 1 | Uniprot\_modification, | 0 | Lymphoma |
| PLCG1 | P19174-2, PLCG1, Isoform 2 of 1-phosphatidylinositol 4,5-bisphosphate phosphodiesterase gamma-1 | 345-345 | - | 2.032e-09 | 23/0/0/ | 43/0/0/ | PI-PLC-X | Family | 1 | Scansite\_motif, | 0 | Lymphoma |
| PNISR | Q8TF01, PNISR, Arginine/serine-rich protein PNISR | 598-631 | - | 0.004525 | 6/0/0/ | 9/0/0/ |  |  | 12 | Scansite\_motif, PhosphoELM\_modification, | 0.4322 | Hepatocellular carcinoma (HCC) |
| POT1 | Q9NUX5, POT1, Protection of telomeres protein 1 | 36-44 | - | 0.003695 | 10/0/0/ | 45/0/0/ | POT1 | Domain | 2 | Region\_of\_interest, PDB, | 0.177 | Lymphoma |
| POTE2\_HUMAN | Q6S8J3-2, POTEE, Isoform 2 of POTE ankyrin domain family member E | 75-82 | - | 0.007971 | 6/0/0/ | 14/0/0/ |  |  | 0 | - | 0.4322 | Thyroid cancer |
| POTEC | B2RU33, POTEC, POTE ankyrin domain family member C | 477-511 | - | 4.504e-07 | 21/0/0/ | 32/0/0/ |  |  | 1 | Scansite\_motif, | 0.523 | Prostate cancer |
| PPP2R1A | P30153, PPP2R1A, Serine/threonine-protein phosphatase 2A 65 kDa regulatory subunit A alpha isoform | 179-184 | - | 2.476e-25 | 68/0/0/ | 121/0/0/ | HEAT\_2 | Family | 6 | PDB, Region\_of\_interest, OMIM, | 0.3065 | Ovarian cancer, Endometrial cancer |
| PPP2R1A | P30153, PPP2R1A, Serine/threonine-protein phosphatase 2A 65 kDa regulatory subunit A alpha isoform | 249-260 | - | 1.923e-09 | 33/0/0/ | 121/0/0/ | HEAT\_2 | Family | 5 | PDB, Scansite\_motif, OMIM, Region\_of\_interest, | 0.2211 | Endometrial cancer |
| PPP6C | O00743, PPP6C, Serine/threonine-protein phosphatase 6 catalytic subunit | 259-270 | PPP6C\_ENST00000451402|296-307 | 0.0001283 | 13/0/0/ | 24/0/0/ |  |  | 0 | - | 0 | Melanoma |
| PPP6R1\_ENST00000412770 | Q9UPN7, PPP6R1, Serine/threonine-protein phosphatase 6 regulatory subunit 1 | 497-529 | - | 0.0008238 | 8/0/0/ | 13/0/0/ | SAPS | Family | 2 | Uniprot\_modification, | 0.1557 | Pancreatic cancer, Renal cell carcinoma |
| PRAMEF11 | A0A087WW85, PRAMEF11, PRAME family member 11 | 82-84 | - | 0.0002397 | 10/0/0/ | 21/0/0/ |  |  | 0 | - | 0.4312 | Pancreatic cancer |
| PRKACA | P17612, PRKACA, cAMP-dependent protein kinase catalytic subunit alpha | 197-206 | - | 0.009202 | 6/0/0/ | 17/0/0/ | Pkinase | Domain | 5 | OMIM, PDB, Uniprot\_modification, PhosphoELM\_modification, | 0.2403 | Adrenal tumor |
| PRKCE | Q02156, PRKCE, Protein kinase C epsilon type | 135-162 | - | 0.009828 | 8/0/0/ | 20/0/0/ |  |  | 1 | PDB, | 0.4618 | Colorectal cancer |
| PRKCG\_ENST00000540413 | P05129, PRKCG, Protein kinase C gamma type | 203-214 | - | 0.005014 | 7/0/0/ | 27/0/0/ | C2 | Domain | 1 | PDB, | 0.3195 | Pancreatic cancer |
| PRKRIR | O43422, PRKRIR, 52 kDa repressor of the inhibitor of the protein kinase | 78-81 | - | 0.009828 | 6/0/0/ | 19/0/0/ | THAP | Domain | 0 | - | 0.3682 | Bile duct/gallbladder cancer, Glioblastoma |
| PRR12 | -, -, - | 414-440 | - | 0.009828 | 6/0/0/ | 19/0/0/ |  |  | 0 | - | 0.4322 | Hepatocellular carcinoma (HCC) |
| PRUNE2\_ENST00000376718 | Q8WUY3, PRUNE2, Protein prune homolog 2 | 2714-2718 | - | 0.0025 | 0/0/10/ | 42/0/10/ |  |  | 0 | - | 0.192 | Acute myeloid leukemia, Other soft tissue tumor |
| PSG2 | P11465, PSG2, Pregnancy-specific beta-1-glycoprotein 2 | 250-264 | - | 0.001935 | 7/0/0/ | 11/0/0/ | Ig\_2 | Domain | 0 | - | 0.4292 | Hepatocellular carcinoma (HCC), Non-small cell lung cancer |
| PTCH1 | Q13635, PTCH1, Protein patched homolog 1 | 1139-1198 | - | 0.001493 | 9/3/11/ | 140/8/69/ | Patched | Family | 0 | - | 0.3244 | Basal cell carcinoma, Skin neoplasm |
| PTCH1 | Q13635, PTCH1, Protein patched homolog 1 | 561-571 | - | 0.0009019 | 8/0/3/ | 140/8/69/ | Patched | Family | 0 | - | 0.2538 | Basal cell carcinoma, Squamous cell carcinoma |
| PTCH1 | Q13635, PTCH1, Protein patched homolog 1 | 681-691 | - | 0.009664 | 9/0/0/ | 140/8/69/ | Patched | Family | 0 | - | 0.3182 | Basal cell carcinoma |
| PTCH1 | Q13635, PTCH1, Protein patched homolog 1 | 923-927 | - | 0.00692 | 2/0/4/ | 140/8/69/ |  |  | 0 | - | 0.1763 | Medulloblastoma, Basal cell carcinoma |
| PTEN | P60484, PTEN, Phosphatidylinositol 3,4,5-trisphosphate 3-phosphatase and dual-specificity protein phosphatase PTEN | 123-142 | - | 5.864e-54 | 274/2/30/ | 907/8/272/ | DSPc | Domain | 9 | OMIM, PDB, Active\_site, | 0.4231 | Endometrial cancer |
| PTEN | P60484, PTEN, Phosphatidylinositol 3,4,5-trisphosphate 3-phosphatase and dual-specificity protein phosphatase PTEN | 150-177 | - | 1.896e-13 | 156/0/5/ | 907/8/272/ | DSPc | Domain | 4 | PDB, OMIM, | 0.5352 | Glioblastoma |
| PTEN | P60484, PTEN, Phosphatidylinositol 3,4,5-trisphosphate 3-phosphatase and dual-specificity protein phosphatase PTEN | 193-204 | - | 0.001953 | 8/0/9/ | 907/8/272/ | PTEN\_C2 | Family | 1 | PDB, | 0.4173 | Glioblastoma |
| PTEN | P60484, PTEN, Phosphatidylinositol 3,4,5-trisphosphate 3-phosphatase and dual-specificity protein phosphatase PTEN | 222-252 | - | 0.0003515 | 43/4/9/ | 907/8/272/ | PTEN\_C2 | Family | 10 | OMIM, Scansite\_motif, PhosphoELM\_modification, PDB, | 0.6333 | - |
| PTPN11 | Q06124-2, PTPN11, Isoform 2 of Tyrosine-protein phosphatase non-receptor type 11 | 502-510 | - | 1.81e-11 | 56/0/0/ | 477/0/0/ | Y\_phosphatase | Domain | 8 | Binding\_site, OMIM, PDB, | 0.4527 | Other haematopoietic/lymphoid disorder, Acute myeloid leukemia |
| PTPN11 | Q06124-2, PTPN11, Isoform 2 of Tyrosine-protein phosphatase non-receptor type 11 | 52-78 | - | 1.273e-151 | 395/0/0/ | 477/0/0/ | SH2 | Domain | 17 | PhosphoELM\_modification, Uniprot\_modification, OMIM, PDB, Scansite\_motif, | 0.3359 | Other haematopoietic/lymphoid disorder |
| Q3ZCN4\_HUMAN | -, -, - | 125-139 | - | 0.0003221 | 10/0/0/ | 12/0/0/ |  |  | 3 | Scansite\_motif, | 0.3783 | Renal cell carcinoma, Prostate cancer |
| RAC1 | P63000, RAC1, Ras-related C3 botulinum toxin substrate 1 | 29-29 | RAC1\_ENST00000356142|29-29 | 4.136e-31 | 63/0/0/ | 73/0/0/ | Ras | Domain | 1 | PDB, | 0.0226 | Melanoma |
| RAD54L2 | Q9Y4B4, RAD54L2, Helicase ARIP4 | 1331-1334 | - | 0.0025 | 0/0/8/ | 16/0/8/ |  |  | 1 | Uniprot\_motif, | 0 | Pancreatic cancer |
| RAF1 | P04049, RAF1, RAF proto-oncogene serine/threonine-protein kinase | 257-261 | - | 0.001724 | 8/0/0/ | 21/0/0/ |  |  | 23 | PhosphoELM\_modification, PDB, Modification\_switch, Uniprot\_modification, OMIM, Ligand\_switch, ELM\_instance, Scansite\_motif, | 0.4799 | Ovarian cancer, Colorectal cancer |
| RAP1B | P61224, RAP1B, Ras-related protein Rap-1b | 9-14 | - | 0.007576 | 5/0/0/ | 6/0/0/ | Ras | Domain | 2 | PDB, NPB\_site, | 0.3689 | Other/various |
| RB1 | P06400, RB1, Retinoblastoma-associated protein | 698-708 | - | 0.005181 | 5/0/9/ | 62/2/97/ | RB\_B | Domain | 4 | Region\_of\_interest, PDB, OMIM, | 0.2921 | Ovarian cancer, Small cell lung cancer |
| RBM23 | Q86U06, RBM23, Probable RNA-binding protein 23 | 393-393 | - | 0.0002914 | 0/7/0/ | 7/7/0/ | RBM39linker | Domain | 0 | - | 0.3195 | Breast cancer |
| RBM4 | Q9BWF3, RBM4, RNA-binding protein 4 | 238-240 | - | 0.0025 | 0/0/6/ | 1/0/6/ |  |  | 1 | Region\_of\_interest, | 0 | Lymphoma |
| RET | P07949, RET, Proto-oncogene tyrosine-protein kinase receptor Ret | 627-640 | - | 4.087e-45 | 39/0/130/ | 420/0/178/ |  |  | 4 | OMIM, | 0.1038 | Thyroid cancer |
| RET | P07949, RET, Proto-oncogene tyrosine-protein kinase receptor Ret | 911-923 | - | 9.082e-119 | 299/0/0/ | 420/0/178/ | Pkinase\_Tyr | Domain | 4 | PDB, OMIM, | 0.085 | Thyroid cancer |
| RFC4 | P35249, RFC4, Replication factor C subunit 4 | 300-327 | - | 0.0005682 | 2/0/7/ | 6/0/7/ | Rep\_fac\_C | Domain | 1 | Scansite\_motif, | 0.3364 | Breast cancer, Colorectal cancer |
| RGPD3 | A6NKT7, RGPD3, RanBP2-like and GRIP domain-containing protein 3 | 812-823 | RGPD3\_ENST00000304514|812-816 | 0.0003985 | 10/0/0/ | 30/0/0/ |  |  | 0 | - | 0.4312 | Endometrial cancer |
| RGPD8 | O14715, RGPD8, RANBP2-like and GRIP domain-containing protein 8 | 1760-1760 | RGPD8\_ENST00000302558|1760-1760 | 2.2e-06 | 14/0/0/ | 21/0/0/ |  |  | 0 | - | 0.23 | Prostate cancer, Glioma |
| RHOA | P61586, RHOA, Transforming protein RhoA | 17-23 | - | 9.314e-44 | 119/0/1/ | 161/0/1/ | Ras | Domain | 2 | NPB\_site, PDB, | 0.0457 | Lymphoma |
| RIF1 | Q5UIP0, RIF1, Telomere-associated protein RIF1 | 1747-1754 | - | 0.0025 | 0/0/16/ | 40/0/16/ |  |  | 0 | - | 0 | Small cell lung cancer |
| RNF43 | Q68DV7, RNF43, E3 ubiquitin-protein ligase RNF43 | 118-186 | - | 0.000114 | 18/0/0/ | 39/0/0/ |  |  | 2 | PDB, Scansite\_motif, | 0.4511 | Pancreatic cancer |
| RP11-82O18.1\_ENST00000360151 | E9PKD4, NPIPA5, Nuclear pore complex-interacting protein family member A5 | 290-290 | RP11-82O18.1|290-290 | 7.77e-05 | 8/0/0/ | 8/0/0/ | NPIP | Family | 0 | - | 0.2493 | Prostate cancer, Glioma |
| RP1L1 | A6NKC6, RP1L1, Retinitis pigmentosa 1-like 1 protein | 1305-1361 | - | 1.654e-09 | 17/4/7/ | 60/4/8/ |  |  | 0 | - | 0.5398 | - |
| RPL10 | P27635, RPL10, 60S ribosomal protein L10 | 92-98 | - | 7.628e-05 | 10/0/0/ | 14/0/0/ | Ribosomal\_L16 | Family | 1 | PDB, | 0.09 | Lymphoma |
| RPSAP58\_ENST00000496398 | A6NE09, RPSAP58, 40S ribosomal protein SA | 111-111 | - | 0.003497 | 6/0/0/ | 8/0/0/ | Ribosomal\_S2 | Family | 1 | PDB, | 0.2801 | Prostate cancer, Renal cell carcinoma |
| RQCD1 | Q92600, RQCD1, Cell differentiation protein RCD1 homolog | 82-88 | - | 0.0006993 | 7/0/0/ | 8/0/0/ | Rcd1 | Family | 1 | PDB, | 0.5389 | - |
| RUNX1T1 | Q06455-4, RUNX1T1, Isoform 4 of Protein CBFA2T1 | 419-434 | - | 0.007594 | 9/0/0/ | 46/0/0/ |  |  | 1 | PDB, | 0.3364 | Ovarian cancer, Colorectal cancer |
| RUNX1 | Q01196-8, RUNX1, Isoform AML-1G of Runt-related transcription factor 1 | 85-254 | - | 9.325e-36 | 184/14/44/ | 206/16/44/ | Runt | Domain | 12 | OMIM, PDB, Scansite\_motif, Uniprot\_modification, Binding\_site, Region\_of\_interest, | 0.3261 | Acute myeloid leukemia |
| RYR2 | Q92736, RYR2, Ryanodine receptor 2 | 1482-1490 | - | 0.00733 | 7/0/0/ | 168/0/0/ | SPRY | Family | 1 | Region\_of\_interest, | 0.4292 | Lymphoma, Pancreatic cancer |
| RYR2 | Q92736, RYR2, Ryanodine receptor 2 | 4059-4098 | RYR2\_ENST00000360064|4065-4092 | 0.009841 | 9/0/0/ | 168/0/0/ | EF-hand\_8 | Domain | 1 | OMIM, | 0.4805 | - |
| S100A7 | P31151, S100A7, Protein S100-A7 | 85-92 | - | 0.007576 | 5/0/0/ | 6/0/0/ |  |  | 1 | PDB, | 0.1864 | Melanoma, Thyroid cancer |
| SACM1L | Q9NTJ5, SACM1L, Phosphatidylinositide phosphatase SAC1 | 488-493 | - | 0.005418 | 6/0/0/ | 10/0/0/ |  |  | 1 | Scansite\_motif, | 0.1248 | Pancreatic cancer |
| SEMA3D | O95025, SEMA3D, Semaphorin-3D | 638-646 | - | 0.002076 | 8/0/0/ | 26/0/0/ |  |  | 0 | - | 0.4618 | Pancreatic cancer |
| SETBP1 | Q9Y6X0, SETBP1, SET-binding protein | 1325-1327 | - | 0.0025 | 0/0/6/ | 145/0/6/ |  |  | 0 | - | 0 | Pancreatic cancer |
| SETBP1 | Q9Y6X0, SETBP1, SET-binding protein | 863-874 | - | 3.048e-50 | 114/0/0/ | 145/0/6/ |  |  | 5 | OMIM, | 0.2766 | Acute myeloid leukemia, Chronic myeloid leukemia |
| SETD2\_ENST00000409792 | Q9BYW2, SETD2, Histone-lysine N-methyltransferase SETD2 | 1615-1668 | SETD2|1112-1183 | 0.0003298 | 16/0/0/ | 76/0/6/ | SET | Family | 4 | PDB, Region\_of\_interest, Binding\_site, | 0.3453 | Renal cell carcinoma |
| SETD2 | Q9BYW2, SETD2, Histone-lysine N-methyltransferase SETD2 | 1257-1259 | - | 0.0025 | 0/0/6/ | 74/0/9/ |  |  | 0 | - | 0 | Renal cell carcinoma |
| SF3A1 | Q15459, SF3A1, Splicing factor 3A subunit 1 | 74-75 | - | 0.008837 | 6/0/0/ | 16/0/0/ | Surp | Family | 1 | PDB, | 0.1763 | Acute myeloid leukemia, Lymphoma |
| SF3B1 | O75533, SF3B1, Splicing factor 3B subunit 1 | 618-630 | - | 3.433e-39 | 157/0/0/ | 915/0/13/ |  |  | 0 | - | 0.3883 | Melanoma, Acute myeloid leukemia |
| SF3B1 | O75533, SF3B1, Splicing factor 3B subunit 1 | 649-711 | - | 1.382e-211 | 643/0/6/ | 915/0/13/ |  |  | 0 | - | 0.3161 | Lymphoma, Acute myeloid leukemia |
| SF3B1 | O75533, SF3B1, Splicing factor 3B subunit 1 | 739-747 | - | 1.134e-12 | 59/0/0/ | 915/0/13/ |  |  | 0 | - | 0.1388 | Lymphoma |
| SF3B1 | O75533, SF3B1, Splicing factor 3B subunit 1 | 780-785 | - | 0.005363 | 8/0/4/ | 915/0/13/ |  |  | 0 | - | 0.3171 | Lymphoma, Acute myeloid leukemia |
| SGK1 | O00141, SGK1, Serine/threonine-protein kinase Sgk1 | 17-77 | SGK1\_ENST00000367857|6-62  SGK1\_ENST00000367858|121-172  SGK1\_ENST00000413996|40-86  SGK1\_ENST00000528577|54-100 | 0.0005003 | 21/0/0/ | 44/0/1/ |  |  | 3 | Scansite\_motif, Region\_of\_interest, Uniprot\_modification, | 0.0871 | Lymphoma |
| SH2B3 | Q9UQQ2, SH2B3, SH2B adapter protein 3 | 208-234 | - | 8.159e-05 | 17/0/0/ | 45/0/0/ | PH | Domain | 0 | - | 0.2238 | Other haematopoietic/lymphoid disorder, Myeloproliferative disease |
| SHANK1 | Q9Y566, SHANK1, SH3 and multiple ankyrin repeat domains protein 1 | 2102-2111 | - | 0.006006 | 3/0/3/ | 39/2/3/ | SAM\_1 | Domain | 0 | - | 0.1763 | Acute myeloid leukemia, Melanoma |
| SIRPA | P78324, SIRPA, Tyrosine-protein phosphatase non-receptor type substrate 1 | 227-233 | - | 0.005418 | 6/0/0/ | 10/0/1/ | C1-set | Domain | 1 | PDB, | 0.3682 | Prostate cancer, Glioma |
| SLC35E3 | Q7Z769, SLC35E3, Solute carrier family 35 member E3 | 65-76 | - | 0.001082 | 6/0/0/ | 6/0/1/ | TPT | Family | 0 | - | 0 | Renal cell carcinoma |
| SMAD3 | P84022, SMAD3, Mothers against decapentaplegic homolog 3 | 422-425 | - | 0.005014 | 7/0/0/ | 27/0/0/ |  |  | 7 | PDB, PhosphoELM\_modification, Uniprot\_modification, | 0.4085 | Pancreatic cancer |
| SMAD4 | Q13485, SMAD4, Mothers against decapentaplegic homolog 4 | 350-386 | - | 2.907e-25 | 117/1/7/ | 300/2/29/ | MH2 | Family | 4 | PDB, OMIM, | 0.4595 | Colorectal cancer, Pancreatic cancer |
| SMAD4 | Q13485, SMAD4, Mothers against decapentaplegic homolog 4 | 523-541 | - | 1.278e-06 | 38/1/2/ | 300/2/29/ | MH2 | Family | 1 | PDB, | 0.2984 | Colorectal cancer, Pancreatic cancer |
| SMARCA4 | P51532, SMARCA4, Transcription activator BRG1 | 1127-1243 | SMARCA4\_ENST00000358026|1135-1243 | 2.551e-08 | 43/0/1/ | 121/0/5/ | Helicase\_C | Family | 1 | OMIM, | 0.6972 | - |
| SMARCA4 | P51532, SMARCA4, Transcription activator BRG1 | 906-920 | - | 0.0003454 | 14/0/0/ | 121/0/5/ | SNF2\_N | Family | 0 | - | 0.2865 | Pancreatic cancer, Medulloblastoma |
| SMARCA4 | P51532, SMARCA4, Transcription activator BRG1 | 972-974 | SMARCA4\_ENST00000358026|966-981 | 0.007146 | 7/0/0/ | 121/0/5/ | SNF2\_N | Family | 0 | - | 0.2205 | Lymphoma |
| SMARCB1 | Q12824, SMARCB1, SWI/SNF-related matrix-associated actin-dependent regulator of chromatin subfamily B member 1 | 184-193 | - | 0.009009 | 4/0/9/ | 44/1/10/ | SNF5 | Family | 12 | Region\_of\_interest, | 0.23 | Brain cancer |
| SMARCB1 | Q12824, SMARCB1, SWI/SNF-related matrix-associated actin-dependent regulator of chromatin subfamily B member 1 | 368-377 | - | 1.291e-05 | 17/0/0/ | 44/1/10/ | SNF5 | Family | 1 | OMIM, | 0.5224 | Meningioma, Lymphoma |
| SMC1A | Q14683, SMC1A, Structural maintenance of chromosomes protein 1A | 771-819 | - | 0.003224 | 12/0/0/ | 40/0/0/ | SMC\_N | Family | 4 | OMIM, | 0.4962 | Acute myeloid leukemia |
| SMO | Q99835, SMO, Smoothened homolog | 408-412 | - | 2.898e-09 | 25/0/0/ | 77/0/0/ | Frizzled | Family | 1 | PDB, | 0.2806 | Bone cancer, Meningioma |
| SMO | Q99835, SMO, Smoothened homolog | 533-535 | - | 1.436e-05 | 15/0/0/ | 77/0/0/ | Frizzled | Family | 1 | PDB, | 0.2384 | Basal cell carcinoma |
| SOCS1 | O15524, SOCS1, Suppressor of cytokine signaling 1 | 130-136 | - | 0.001888 | 1/0/11/ | 24/1/118/ | SH2 | Domain | 0 | - | 0 | Lymphoma |
| SOCS1 | O15524, SOCS1, Suppressor of cytokine signaling 1 | 181-191 | - | 0.001888 | 1/0/16/ | 24/1/118/ | SOCS\_box | Domain | 1 | Region\_of\_interest, | 0 | Lymphoma |
| SPOP | O43791, SPOP, Speckle-type POZ protein | 75-140 | - | 1.396e-22 | 79/0/1/ | 91/0/1/ | MATH | Domain | 4 | PDB, Scansite\_motif, Region\_of\_interest, | 0.1502 | Prostate cancer |
| SPTA1 | P02549, SPTA1, Spectrin alpha chain, erythrocytic 1 | 1798-1802 | - | 0.008333 | 0/0/5/ | 131/0/5/ | Spectrin | Domain | 0 | - | 0 | Breast cancer |
| SRSF2 | Q01130, SRSF2, Serine/arginine-rich splicing factor 2 | 94-102 | - | 1.037e-193 | 299/3/310/ | 314/4/310/ |  |  | 1 | PhosphoELM\_modification, | 0.2194 | Chronic myeloid leukemia, Acute myeloid leukemia |
| STAG2 | Q8N3U4, STAG2, Cohesin subunit SA-2 | 184-224 | STAG2\_ENST00000218089|209-219 | 0.001189 | 8/1/6/ | 62/1/11/ | STAG | Family | 1 | PDB, | 0.4376 | Bladder cancer |
| STAG2 | Q8N3U4, STAG2, Cohesin subunit SA-2 | 733-740 | - | 0.006098 | 3/0/5/ | 62/1/11/ |  |  | 2 | PhosphoELM\_modification, PDB, | 0.1043 | Bladder cancer |
| STAT3 | P40763, STAT3, Signal transducer and activator of transcription 3 | 640-663 | - | 6.129e-48 | 119/3/0/ | 151/3/3/ | SH2 | Domain | 4 | PDB, OMIM, | 0.0804 | Lymphoma |
| STAT5B | P51692, STAT5B, Signal transducer and activator of transcription 5B | 642-648 | - | 1.058e-06 | 17/0/0/ | 43/0/0/ | SH2 | Domain | 2 | OMIM, PDB, | 0.062 | Lymphoma |
| STAT6 | P42226, STAT6, Signal transducer and activator of transcription 6 | 414-420 | - | 1.539e-05 | 14/0/0/ | 40/0/0/ | STAT\_bind | Domain | 0 | - | 0.0713 | Lymphoma |
| STK11 | Q15831, STK11, Serine/threonine-protein kinase STK11 | 194-199 | - | 0.0005944 | 13/0/0/ | 84/0/9/ | Pkinase | Domain | 2 | PDB, OMIM, | 0.4222 | Non-small cell lung cancer |
| STK11 | Q15831, STK11, Serine/threonine-protein kinase STK11 | 277-281 | - | 0.0005944 | 13/0/0/ | 84/0/9/ | Pkinase | Domain | 1 | PDB, | 0.3675 | Non-small cell lung cancer, Stomach cancer |
| SUSD2 | Q9UGT4, SUSD2, Sushi domain-containing protein 2 | 70-91 | - | 0.008837 | 6/0/0/ | 16/0/0/ |  |  | 1 | Scansite\_motif, | 0.3682 | Prostate cancer, Neuroblastoma |
| SgK069 | P0C263, SBK2, Serine/threonine-protein kinase SBK2 | 125-129 | SBK2|125-129 | 0.002609 | 7/0/0/ | 13/0/0/ | Pkinase | Domain | 0 | - | 0.1657 | Ovarian cancer, Hepatocellular carcinoma (HCC) |
| TBC1D2B | Q9UPU7-2, TBC1D2B, Isoform 2 of TBC1 domain family member 2B | 903-903 | - | 0.006192 | 6/0/0/ | 11/0/0/ |  |  | 0 | - | 0.3441 | Prostate cancer |
| TCEB1 | Q15369, TCEB1, Transcription elongation factor B polypeptide 1 | 79-79 | - | 0.0002057 | 8/0/0/ | 9/0/0/ | Skp1\_POZ | Domain | 1 | PDB, | 0 | Renal cell carcinoma |
| TET1\_ENST00000373644 | Q8NFU7, TET1, Methylcytosine dioxygenase TET1 | 95-95 | - | 0.0007055 | 10/0/0/ | 74/0/1/ |  |  | 0 | - | 0.09 | Acute myeloid leukemia |
| TET2 | Q6N021, TET2, Methylcytosine dioxygenase TET2 | 1148-1427 | - | 2.576e-41 | 229/0/17/ | 439/1/36/ | Tet\_JBP | Domain | 6 | Binding\_site, Region\_of\_interest, PDB, | 0.4089 | Acute myeloid leukemia |
| TET2 | Q6N021, TET2, Methylcytosine dioxygenase TET2 | 1714-1723 | - | 0.003459 | 13/0/0/ | 439/1/36/ | Tet\_JBP | Domain | 0 | - | 0.4445 | Myeloproliferative disease, Acute myeloid leukemia |
| TET2 | Q6N021, TET2, Methylcytosine dioxygenase TET2 | 1861-1907 | - | 2.777e-18 | 75/0/10/ | 439/1/36/ | Tet\_JBP | Domain | 2 | Region\_of\_interest, | 0.3537 | Chronic myeloid leukemia, Acute myeloid leukemia |
| TET2 | Q6N021, TET2, Methylcytosine dioxygenase TET2 | 22-25 | - | 0.0001215 | 16/0/0/ | 439/1/36/ |  |  | 0 | - | 0.0647 | Acute myeloid leukemia |
| TFAP4 | Q01664, TFAP4, Transcription factor AP-4 | 47-58 | - | 0.001131 | 7/0/0/ | 9/0/0/ | HLH | Domain | 1 | Scansite\_motif, | 0.2205 | Lymphoma |
| TGFBR2 | P37173, TGFBR2, TGF-beta receptor type-2 | 446-448 | - | 0.002956 | 8/0/0/ | 54/0/2/ | Pkinase\_Tyr | Domain | 1 | OMIM, | 0.1557 | Squamous cell carcinoma, Pancreatic cancer |
| TGFBR2 | P37173, TGFBR2, TGF-beta receptor type-2 | 521-530 | - | 0.008034 | 9/0/0/ | 54/0/2/ | Pkinase\_Tyr | Domain | 3 | OMIM, | 0.4644 | Pancreatic cancer |
| TINAG | Q9UJW2, TINAG, Tubulointerstitial nephritis antigen | 191-191 | TINAG\_ENST00000370864|173-173 | 0.00431 | 7/0/0/ | 21/0/0/ |  |  | 2 | Scansite\_motif, | 0.4292 | Lymphoma, Pancreatic cancer |
| TMEM225 | Q6GV28, TMEM225, Transmembrane protein 225 | 180-221 | - | 0.001664 | 10/0/0/ | 12/0/0/ |  |  | 0 | - | 0.5993 | - |
| TNFRSF14 | Q92956, TNFRSF14, Tumor necrosis factor receptor superfamily member 14 | 1-26 | - | 0.007378 | 12/0/0/ | 29/3/0/ |  |  | 1 | Scansite\_motif, | 0 | Lymphoma |
| TNIK | Q9UKE5, TNIK, TRAF2 and NCK-interacting protein kinase | 1029-1046 | - | 0.005485 | 7/0/0/ | 33/0/0/ |  |  | 2 | Region\_of\_interest, Scansite\_motif, | 0.2781 | Pancreatic cancer, Lymphoma |
| TP53 | P04637, TP53, Cellular tumor antigen p53 | 125-309 | TP53\_ENST00000269305|82-287  TP53\_ENST00000413465|82-258  TP53\_ENST00000414315|40-47  TP53\_ENST00000414315|58-63  TP53\_ENST00000420246|105-287  TP53\_ENST00000455263|108-287  TP53\_ENST00000545858|33-50  TP53\_ENST00000545858|58-70  TP53\_ENST00000545858|79-86  TP53\_ENST00000545858|97-102  TP53\_ENST00000545858|136-155 | 0 | 12064/60/1222/ | 12555/64/1388/ | P53 | Domain | 73 | Scansite\_motif, Region\_of\_interest, OMIM, PDB, Uniprot\_modification, PhosphoELM\_modification, Uniprot\_motif, ELM\_instance, | 0.7799 | Ovarian cancer, Small cell lung cancer, Esophageal cancer, Acute myeloid leukemia, Colorectal cancer, Basal cell carcinoma, Bladder cancer, Other soft tissue tumor, Bile duct/gallbladder cancer, Stomach cancer, Endometrial cancer, Prostate cancer, Pancreatic cancer, Other/various, Non-small cell lung cancer, Hepatocellular carcinoma (HCC), Glioblastoma, Squamous cell carcinoma, Lymphoma, Breast cancer, Glioma |
| TP53 | P04637, TP53, Cellular tumor antigen p53 | 335-346 | - | 0.0004167 | 65/0/10/ | 12555/64/1388/ | P53\_tetramer | Motif | 10 | ELM\_instance, Uniprot\_motif, OMIM, Region\_of\_interest, Ligand\_switch, PDB, | 0.7288 | - |
| TRAF7 | Q6Q0C0, TRAF7, E3 ubiquitin-protein ligase TRAF7 | 384-394 | - | 0.001538 | 7/0/3/ | 62/1/5/ | WD40 | Repeat | 0 | - | 0.09 | Meningioma |
| TRAF7 | Q6Q0C0, TRAF7, E3 ubiquitin-protein ligase TRAF7 | 520-521 | - | 0.0001356 | 12/0/0/ | 62/1/5/ | WD40 | Repeat | 0 | - | 0 | Meningioma |
| TRAF7 | Q6Q0C0, TRAF7, E3 ubiquitin-protein ligase TRAF7 | 536-536 | - | 0.003071 | 8/0/0/ | 62/1/5/ | WD40 | Repeat | 0 | - | 0 | Meningioma |
| TRAF7 | Q6Q0C0, TRAF7, E3 ubiquitin-protein ligase TRAF7 | 637-641 | - | 0.00143 | 9/0/0/ | 62/1/5/ | WD40 | Repeat | 0 | - | 0.0966 | Meningioma |
| TRIM49C | P0CI26, TRIM49C, Tripartite motif-containing protein 49C | 298-298 | TRIM49C\_ENST00000448984|298-298 | 0.005418 | 6/0/0/ | 10/0/0/ |  |  | 0 | - | 0.3682 | Colorectal cancer, Breast cancer |
| TRIO | O75962, TRIO, Triple functional domain protein | 156-156 | - | 0.001082 | 0/6/0/ | 49/6/0/ | CRAL\_TRIO | Domain | 0 | - | 0.1248 | Glioma |
| TRRAP | Q9Y4A5-2, TRRAP, Isoform 2 of Transformation/transcription domain-associated protein | 722-722 | TRRAP\_ENST00000359863|722-722 | 0.007001 | 7/0/0/ | 99/0/0/ |  |  | 0 | - | 0.2205 | Melanoma |
| TSC2 | P49815, TSC2, Tuberin | 1743-1751 | - | 7.813e-09 | 1/0/36/ | 46/0/42/ |  |  | 3 | OMIM, | 0.2059 | Renal cell carcinoma, Squamous cell carcinoma |
| TSHR | A0A0A0MTJ0, TSHR, Thyrotropin receptor | 281-281 | - | 0.001803 | 9/0/0/ | 229/0/13/ |  |  | 1 | OMIM, | 0 | Thyroid cancer |
| TSHR | A0A0A0MTJ0, TSHR, Thyrotropin receptor | 453-457 | - | 5.749e-09 | 30/0/0/ | 229/0/13/ | 7tm\_1 | Family | 1 | OMIM, | 0.0405 | Thyroid cancer |
| TSHR | A0A0A0MTJ0, TSHR, Thyrotropin receptor | 486-486 | - | 0.0008833 | 10/0/0/ | 229/0/13/ | 7tm\_1 | Family | 1 | OMIM, | 0 | Thyroid cancer |
| TSHR | A0A0A0MTJ0, TSHR, Thyrotropin receptor | 613-639 | - | 4.341e-40 | 125/0/12/ | 229/0/13/ | 7tm\_1 | Family | 5 | OMIM, | 0.012 | Thyroid cancer |
| TUBA3C | Q13748, TUBA3C, Tubulin alpha-3C/D chain | 98-138 | - | 0.009515 | 12/0/0/ | 38/0/0/ | Tubulin | Domain | 1 | PDB, | 0.5161 | Breast cancer |
| U2AF1 | Q01081, U2AF1, Splicing factor U2AF 35 kDa subunit | 156-159 | U2AF1\_ENST00000380276|156-159 | 3.088e-19 | 58/2/0/ | 145/2/0/ | zf-CCCH | Family | 0 | - | 0.2641 | Acute myeloid leukemia |
| U2AF1 | Q01081, U2AF1, Splicing factor U2AF 35 kDa subunit | 34-35 | U2AF1\_ENST00000380276|28-34 | 2.898e-28 | 77/0/0/ | 145/2/0/ | zf-CCCH | Family | 0 | - | 0.3501 | Acute myeloid leukemia |
| UBBP4\_ENST00000584755 | J3QLP7, UBBP4, Protein UBBP4 | 50-50 | UBBP4|50-50  UBBP4\_ENST00000578713|50-50 | 0.004917 | 7/0/0/ | 26/0/0/ | ubiquitin | Domain | 1 | PDB, | 0.3195 | Glioma |
| UBBP4\_ENST00000584755 | J3QLP7, UBBP4, Protein UBBP4 | 73-73 | UBBP4|73-73  UBBP4\_ENST00000578713|73-73 | 0.002076 | 8/0/0/ | 26/0/0/ | ubiquitin | Domain | 1 | PDB, | 0.3839 | Glioma |
| URGCP | Q8TCY9-4, URGCP, Isoform 4 of Up-regulator of cell proliferation | 392-415 | - | 0.009202 | 6/0/0/ | 17/0/0/ |  |  | 1 | Scansite\_motif, | 0.4322 | Prostate cancer |
| USP8 | P40818, USP8, Ubiquitin carboxyl-terminal hydrolase 8 | 713-722 | - | 0.0004558 | 9/0/1/ | 28/0/1/ |  |  | 4 | Scansite\_motif, Uniprot\_modification, | 0.3235 | Lymphoma, Other soft tissue tumor |
| USP9X | Q93008-1, USP9X, Isoform 2 of Probable ubiquitin carboxyl-terminal hydrolase FAF-X | 2345-2345 | USP9X\_ENST00000324545|2352-2352 | 0.0002482 | 11/0/0/ | 46/0/0/ |  |  | 0 | - | 0 | Ovarian cancer |
| VHL | P40337, VHL, Von Hippel-Lindau disease tumor suppressor | 145-193 | - | 6.93e-20 | 253/2/63/ | 754/8/265/ | VHL | Domain | 28 | PDB, OMIM, Region\_of\_interest, | 0.1327 | Renal cell carcinoma |
| VHL | P40337, VHL, Von Hippel-Lindau disease tumor suppressor | 54-136 | - | 9.711e-36 | 450/6/197/ | 754/8/265/ | VHL | Domain | 39 | OMIM, PDB, Region\_of\_interest, | 0.128 | Renal cell carcinoma |
| WASH3P | C4AMC7, WASH3P, Putative WAS protein family homolog 3 | 368-410 | - | 1.05e-14 | 34/0/0/ | 36/0/0/ |  |  | 3 | Region\_of\_interest, | 0.5367 | Renal cell carcinoma |
| WDR37 | Q9Y2I8, WDR37, WD repeat-containing protein 37 | 303-303 | - | 0.008429 | 6/0/0/ | 15/0/0/ |  |  | 0 | - | 0 | Ovarian cancer |
| WHSC1 | O96028, WHSC1, Histone-lysine N-methyltransferase NSD2 | 1099-1099 | WHSC1\_ENST00000382888|447-447 | 0.0001802 | 11/0/0/ | 33/0/0/ | SET | Family | 0 | - | 0.0844 | Lymphoma |
| WISP1 | O95388, WISP1, WNT1-inducible-signaling pathway protein 1 | 161-171 | - | 0.006192 | 6/0/0/ | 11/0/0/ | VWC | Family | 1 | Scansite\_motif, | 0.2403 | Pancreatic cancer |
| WT1 | P19544, WT1, Wilms tumor protein | 293-317 | WT1\_ENST00000332351|345-385  WT1\_ENST00000379079|133-183 | 3.391e-05 | 19/4/1/ | 107/4/190/ | WT1 | Family | 3 | OMIM, PDB, Scansite\_motif, | 0.3002 | Lymphoma, Acute myeloid leukemia |
| WT1 | P19544, WT1, Wilms tumor protein | 384-397 | - | 6.253e-13 | 41/0/87/ | 107/4/190/ |  |  | 9 | OMIM, PDB, Region\_of\_interest, Scansite\_motif, | 0.1973 | Acute myeloid leukemia |
| XPO1 | O14980, XPO1, Exportin-1 | 565-574 | - | 8.302e-15 | 39/0/0/ | 63/0/0/ |  |  | 2 | PDB, Region\_of\_interest, | 0.1238 | Lymphoma |
| YSK4 | H7C041, MAP3K19, Mitogen-activated protein kinase kinase kinase 19 (Fragment) | 500-512 | - | 0.008837 | 6/0/0/ | 16/0/0/ | Pkinase | Domain | 0 | - | 0.3042 | Breast cancer, Stomach cancer, Bladder cancer |
| ZDHHC11\_ENST00000424784 | Q9H8X9, ZDHHC11, Probable palmitoyltransferase ZDHHC11 | 298-303 | ZDHHC11|298-303 | 0.008429 | 6/0/0/ | 15/0/0/ |  |  | 0 | - | 0.2403 | Prostate cancer |
| ZNF408 | Q9H9D4, ZNF408, Zinc finger protein 408 | 194-197 | - | 0.000125 | 0/0/12/ | 11/0/12/ |  |  | 0 | - | 0.3042 | Lymphoma, Ovarian cancer, Pancreatic cancer |
| ZNF536 | O15090, ZNF536, Zinc finger protein 536 | 607-638 | - | 0.008292 | 9/0/0/ | 60/0/0/ |  |  | 0 | - | 0.4644 | Lymphoma |
| ZNF709\_ENST00000397732 | Q8N972, ZNF709, Zinc finger protein 709 | 412-413 | - | 0.007453 | 6/0/0/ | 13/0/0/ |  |  | 0 | - | 0.192 | Prostate cancer, Glioma |
| ZNF75D | P51815, ZNF75D, Zinc finger protein 75D | 5-18 | - | 0.009202 | 6/0/0/ | 17/0/0/ |  |  | 0 | - | 0.3682 | Acute myeloid leukemia, Bladder cancer |
| ZNF799\_ENST00000430385 | Q96GE5, ZNF799, Zinc finger protein 799 | 589-589 | ZNF799|376-376 | 0.003993 | 7/0/0/ | 19/0/0/ | zf-C2H2\_6 | Domain | 0 | - | 0.2205 | Prostate cancer |
| ZNF814 | B7Z6K7, ZNF814, Putative uncharacterized zinc finger protein 814 | 337-337 | - | 6.451e-11 | 27/0/0/ | 51/0/0/ |  |  | 0 | - | 0.4235 | Pancreatic cancer, Squamous cell carcinoma |
| ZNF814 | B7Z6K7, ZNF814, Putative uncharacterized zinc finger protein 814 | 404-404 | - | 5.092e-05 | 13/0/0/ | 51/0/0/ |  |  | 0 | - | 0.4096 | Glioma |
| ZNF83 | P51522, ZNF83, Zinc finger protein 83 | 290-290 | - | 0.006192 | 6/0/0/ | 11/0/0/ | zf-C2H2 | Domain | 0 | - | 0.1248 | Bladder cancer |
| ZNF844 | Q08AG5, ZNF844, Zinc finger protein 844 | 503-508 | - | 0.00431 | 7/0/0/ | 21/0/0/ |  |  | 0 | - | 0.2647 | Prostate cancer, Renal cell carcinoma |
| ZNF91 | Q05481, ZNF91, Zinc finger protein 91 | 305-335 | - | 0.006585 | 9/0/0/ | 34/0/0/ | zf-C2H2 | Domain | 0 | - | 0.2595 | Glioma, Prostate cancer |

| Protein Name | Other names | Region | Isoforms | P value | Mutations in region | Total mutations | Pfam domain name | Pfam domain type | Number of annotations | Types of annotations | Heterogeineity index | Dominant cancer tissue type |
| --- | --- | --- | --- | --- | --- | --- | --- | --- | --- | --- | --- | --- |
|  |  |  |  |  |  |  |  |  |  |  |  |  |
