# Supplementary figures and images for: Systematic analysis of somatic mutations driving cancer: uncovering functional protein regions in disease development
Source: Biol Direct. 2016 May 5;11:23. doi: 10.1186/s13062-016-0125-6 (PMC4858844; doi:10.1186/s13062-016-0125-6)

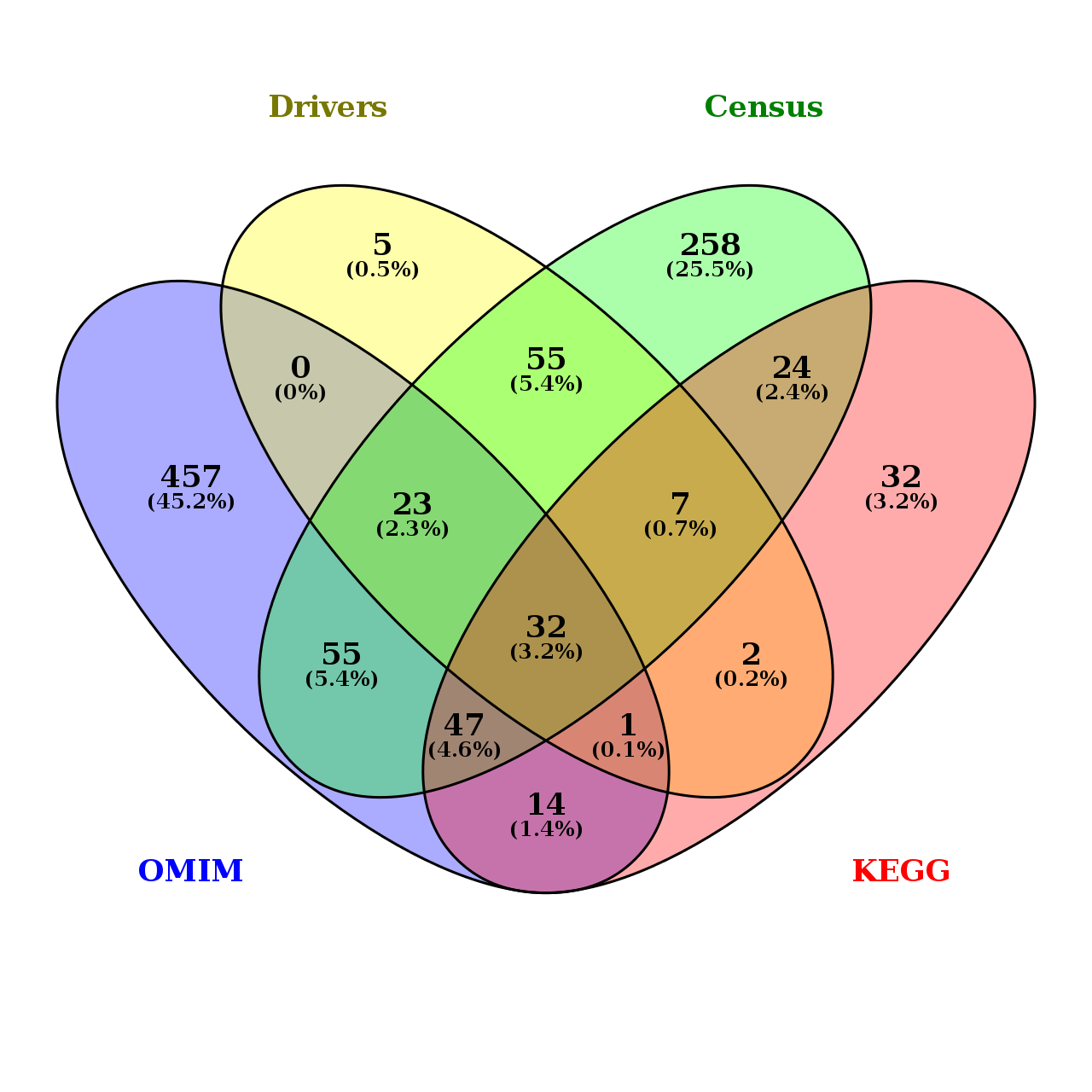

Supplement: Additional file 4: — Cancer driver gene datasets. (PNG 131 kb) [file 13062_2016_125_MOESM4_ESM.png]
